# Potent and selective indole-based inhibitors targeting disease-transmitting mosquitoes

**DOI:** 10.1039/d5md00797f

**Published:** 2025-11-19

**Authors:** R. Rajeshwari, V. Duvauchelle, C. Lindgren, K. Stangner, S. Knutsson, N. Forsgren, F. Ekström, L. Kamau, A. Linusson

**Affiliations:** a Department of Chemistry, Umeå University Sweden anna.linusson@umu.se; b Swedish Defense Research Agency Umeå Sweden; c Centre of Biotechnology Research and Development, Kenya Medical Research Institute Nairobi Kenya

## Abstract

Vector control with insecticides is an important preventive measure against mosquito-borne infectious diseases, such as malaria and dengue. The intensive usage of few insecticides has resulted in emerging resistance in mosquitoes, and unwanted off-target toxic effects. Therefore, there is great interest in alternative active ingredients. Here, we explore indole-based compounds as selective inhibitors against acetylcholinesterase 1 (AChE1) from the disease-transmitting mosquitoes *Anopheles gambiae* (*An. gambiae*, *Ag*AChE1) and *Aedes aegypti* (*Ae. aegypti*, *Ae*AChE1) as potential candidates for future insecticides used in vector control. Three sets of compounds were designed to explore their structure–activity relationship, and investigate their potentials regarding potency and selectivity. 26 indole-based compounds were synthesized and biochemically evaluated for inhibition against *Ag*AChE1, *Ae*AChE1, and human AChE (*h*AChE). The compounds were shown to be potent inhibitors against AChE1, and selective for AChE1 over *h*AChE. *N*-Methylation of the indole moiety clearly increased the inhibition potency, and a bulkier benzyl moiety improved the selectivity. X-ray crystallography shows that the inhibitors bind at the bottom of the active site gorge of mouse AChE (*m*AChE), while molecular dynamics simulations revealed different binding poses in *m*AChE and *Ag*AChE1. Four potent and selective inhibitors were subjected to *in vivo* mosquito testing. Topical application showed strong insecticidal effects on *An. gambiae* and *Ae. aegypti*, highlighting this compound class as an interesting alternative for future insecticide research.

## Abstract 2

Four indole-based compounds demonstrated strong insecticidal effects against mosquitoes by acetylcholinesterase (AChE) inhibition. A selective profile over human AChE suggests a promising alternative in the development of future insecticides.

## Graphical abstract

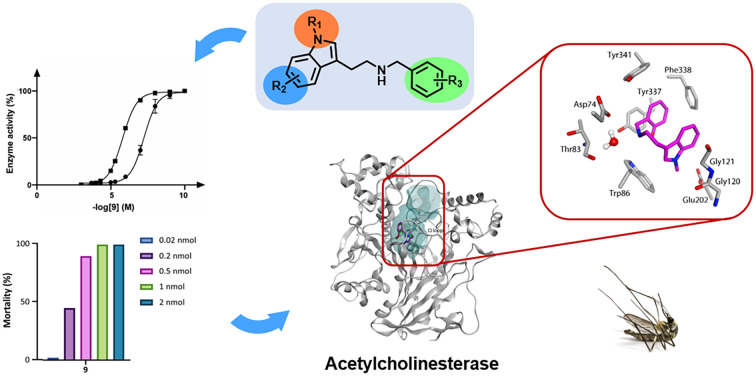

## Introduction

*Anopheles gambiae* (*An. gambiae*) and *Aedes aegypti* (*Ae. aegypti*) are disease-transmitting mosquitoes, so called vectors, which spread diseases such as malaria, dengue, chikungunya, yellow fever and Zika. Vector control by the use of insecticides is an important preventive measure against mosquito-borne infectious diseases. These insecticides belong mainly to four chemical classes, organophosphates, carbamates, hydrocarbons, and pyrethroids. In malaria-endemic countries consistent implementation of insecticide-treated mosquito nets with pyrethroids have resulted in significant public health impact.^1–3^ Unfortunately, the widespread usage of insecticides has also led to the development and spread of insecticide-resistant mosquito populations, for example metabolic detoxification in mosquitoes and target site structural mutations,^4,5^ resulting in a need for new insecticides.

**Fig. 1:**
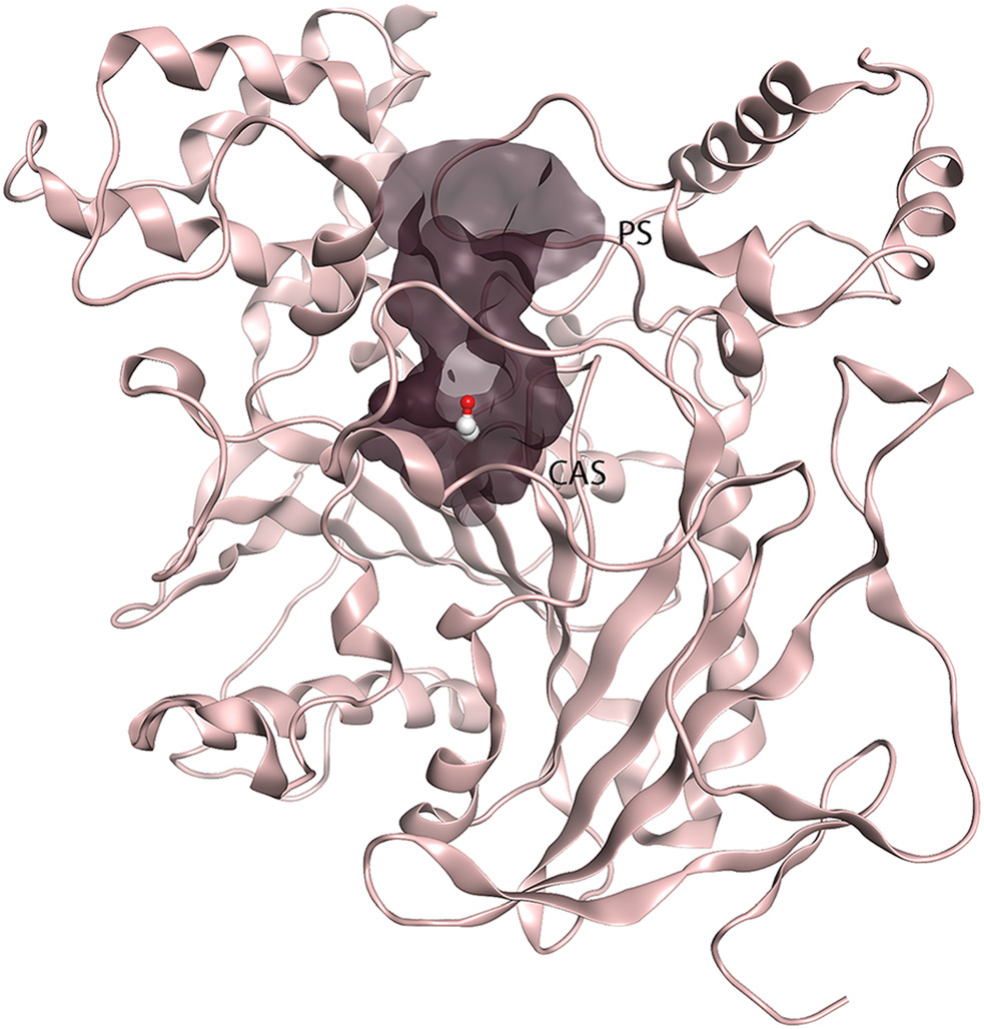
The 3D structure of AChE1 of *An. gambiae* (*Ag*AChE1), with the active site gorge displayed as a surface in grey and the conserved catalytic serine highlighted in ball and stick (PDB: 5X61). The peripheral (PS) and catalytic sites (CAS) are marked.

Acetylcholinesterase (AChE) is the insecticidal target of organophosphates and carbamates, while hydrocarbons and pyrethroids target voltage-gated channels; both targets are present in the mosquitoes' nervous system. The insecticidal activity of organophosphates and carbamates is achieved through covalent modification (phosphorylation or carbamoylation) of the conserved catalytic serine residue at the bottom of the deep active site gorge of AChE (Fig. 1). The active sites of AChEs of different organisms are highly conserved. Hence, the currently used organophosphates and carbamates are nonspecific and inhibit AChEs from different organisms, including human (*h*AChE),^6,7^ leading to off-target toxicity.^8^ AChE is an essential enzyme that terminates cholinergic nerve signaling by hydrolyzing the neurotransmitter acetylcholine (ACh).^9^ Inhibition of AChE leads to continuous nerve signaling due to accumulation of ACh in the synaptic cleft, and eventually to paralysis and death of the organism. The active site gorge is lined with aromatic amino acid residues, and consists of the peripheral site (PS) at the entrance of the gorge and the catalytic site (CAS) at the bottom (Fig. 1).^10^ In mosquitoes and many other insects, AChE is encoded by two genes called *ace-1* and *ace-2*,^11,12^ in contrast to vertebrates that only have one gene. The *ace-1* encoded AChE1 is the main catalytically active enzyme in mosquitoes.^12^

*Ag*AChE1 and mammalian AChEs, such as *h*AChE and mouse (*m*AChE), share highly similar overall structures, including the conserved catalytic triad in the active site gorge. However, notable differences exist in the loops that line the entrance to the active site gorge.^13^ These loops vary in length, conformation, and residue composition between mosquito and mammalian AChEs, creating differences in the shape and accessibility of the gorge. These variations may influence ligand binding and offer opportunities for the design of selective inhibitors. To meet the need for new insecticides while minimizing off-target toxicity, selective inhibition of mosquito AChE1 over hAChE using noncovalent inhibitors has emerged as an attractive strategy.^14^ In recent years, significant efforts have been made to discover potent noncovalent mosquito AChE1 inhibitors with *in vivo* insecticidal activity.^15–18^ Here, we have designed and synthesized indole-based compounds to target AChE1 from *An. gambiae* and *Ae. aegypti* (*Ag*AChE1 and *Aa*AChE1). The synthesized compounds were evaluated *in vitro* through an activity-based assay to investigate potency and selectivity. The interaction patterns of the indole-based inhibitors in complex with *m*AChE were explored by X-ray crystallography and molecular dynamics (MD) simulations. Finally, a few inhibitors were subjected to *in vivo* testing to establish their insecticidal activity against the mosquito species *An. gambiae* and *Ae. aegypti*.

## Results and discussion

### Identification of indoles as biologically active scaffolds and inhibitors against AChE1

**Fig. 2:**
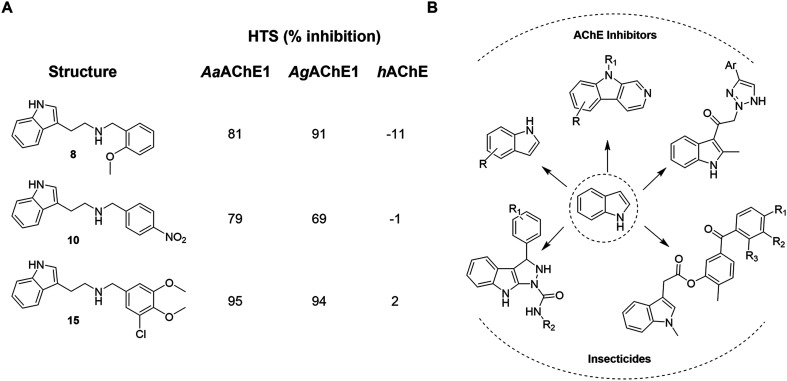
(A) Chemical structures and HTS inhibition data of the indole-based hit compounds. (B) Previously reported indole-based AChE inhibitors (top)^25,26^ and insecticides (bottom).^27,29^

In a previously reported high-throughput screening (HTS) campaign against recombinant AChE1 performed in our laboratory,^14^ the indole-based compounds 8, 10, and 15 were identified as hits (Fig. 2A). The hit compounds significantly reduced the enzymatic activity of *Aa*AChE1 and *Ag*AChE1 at the tested concentration of 50 μM, while not showing any inhibitory activity against *h*AChE. The indole moiety is known as a versatile heterocyclic fragment in medicinal chemistry and to confer antitubercular,^19,20^ antibacterial,^21,22^ antiviral^23^ and anticancer activities.^24^ Interestingly, indole derivatives have also been reported as potential drug candidates against central nervous system disorders and as AChE inhibitors (Fig. 2B).^25,26^ Indole-based compounds have also previously been reported as insecticides, although without any knowledge regarding mechanism of action (Fig. 2B).^27,28^ The hit compounds were evaluated *in silico* and, to some extent, experimentally for toxicity endpoints and lead-likeness, and were considered suitable for further development (Tables S1 and S2 and Fig. S1).

### Design strategy and synthesis of three sets of indole-based inhibitors

**Fig. 3:**
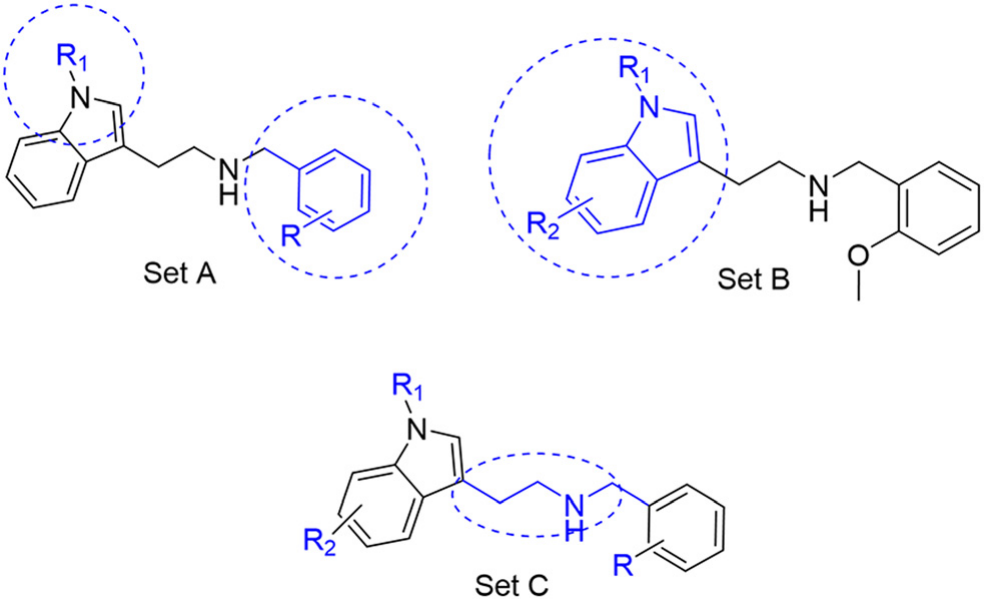
Design strategy of the three sets of molecules (A–C) based on the hit compounds. The explored parts of each set are indicated in blue.

Three sets A–C were designed based on the hit compounds from the HTS (Fig. 3). The modifications were chosen to balance electronic and steric effects while maintaining synthetic feasibility. Set A comprised of 12 compounds (6–17) including the hit compounds 8, 10, and 15, and were designed to investigate the effect of *N*-methylation (N-Me) of the indole moiety, and the effect of varying the benzyl moiety (Table 1). Set B comprised of nine compounds (18–26) in which the methoxybenzyl moiety was kept constant while the substituents of the indole (both N–H and N-Me indoles) were varied (Table 2). Set C was designed to investigate changes of the aliphatic linker through methylation of the secondary amines (29–30), and cyclization of the linker chain compounds (31–33) with the intention of introducing rigidity within the structure (Table 3).

**Table 1:** Chemical structures and IC_50_ values of N–H and *N*-methylated indoles in set A

ID	Structure	*Ag*AChE1	*Aa*AChE1	*h*AChE	S.R^b^
		IC_50_ μM^a^	IC_50_ μM^a^	IC_50_ μM^a^	
6	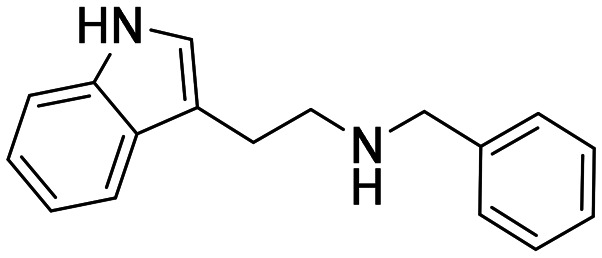	38 (33–44)	34 (27–41)	>500	>13
7	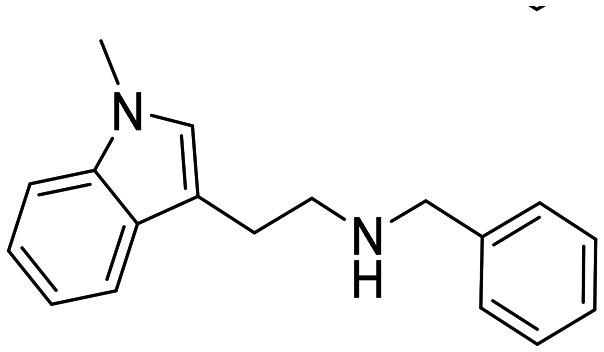	11 (4.1–123)	n.d.	115 (94–145)	10
8	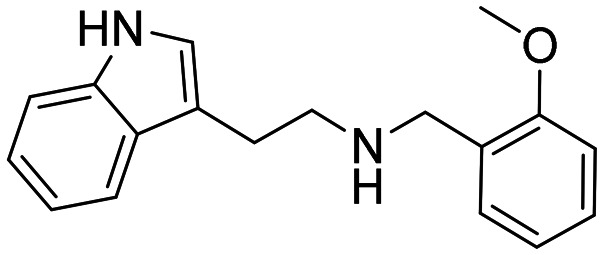	1.6 (1.3–2.0)	1.1 (0.9–1.4)	70 (39–330)	44
9	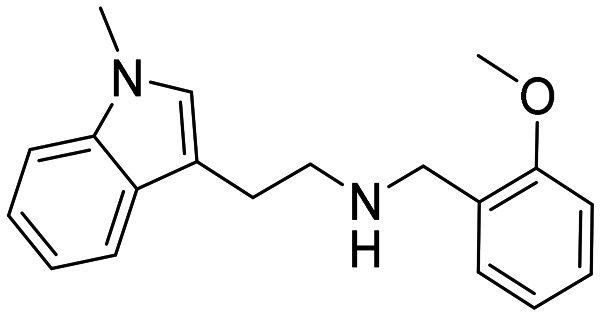	0.06 (0.044–0.070)	n.d.	1.6 (1.5–1.7)	27
10	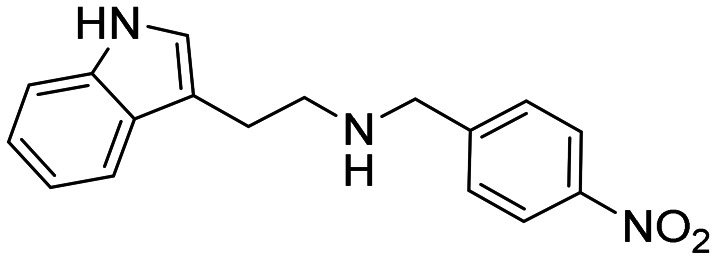	2.2 (1.8–2.6)	1.7 (1.4–2.1)	85 (46–321)	39
11	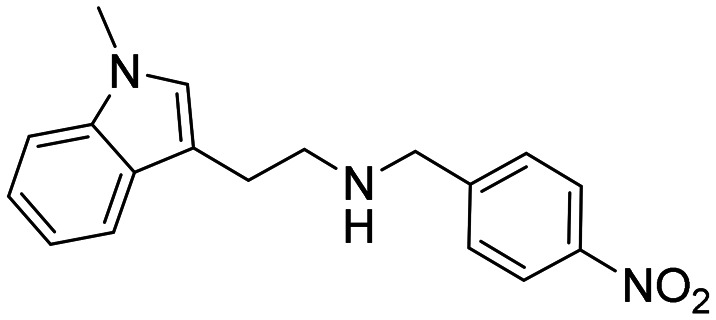	0.2 (0.15–0.23)	n.d.	6.6 (5.4–8.0)	33
12	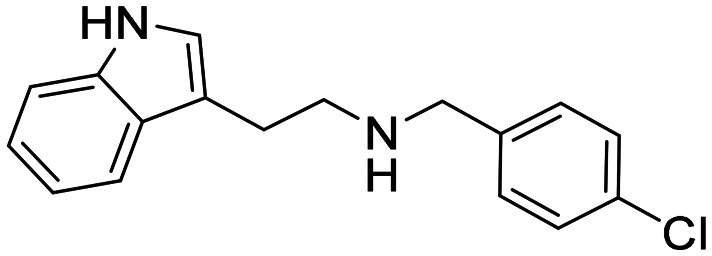	20 (11–43)	n.d.	>200	13
13	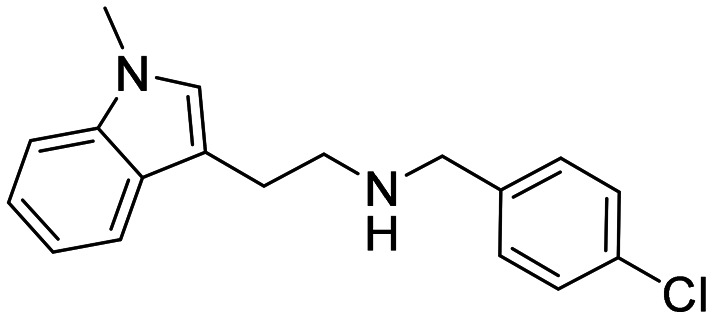	1.5 (1.0–2.2)	n.d	74 (60–95)	49
14	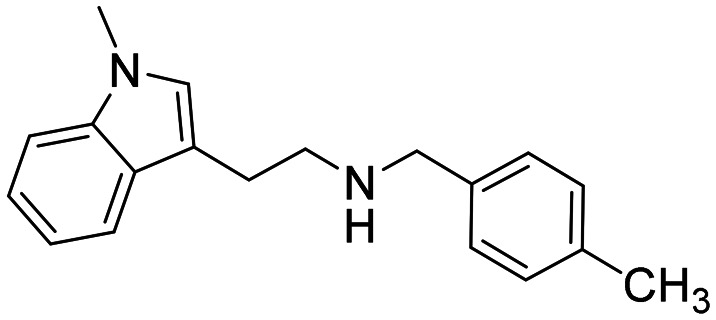	13 (6.3–36)	n.d.	124 (98–169)	10
15	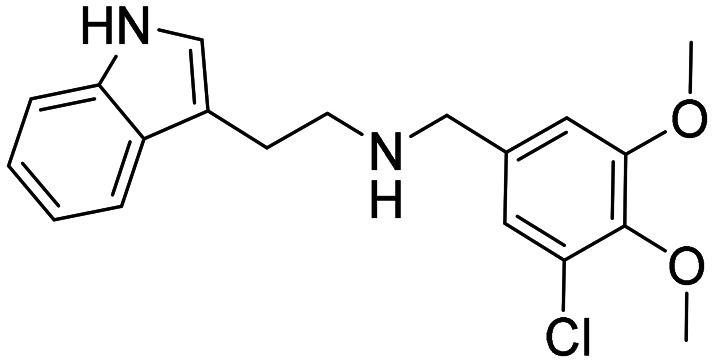	0.72 (0.67–0.78)	0.71 (0.62–0.81)	284 (166–903)	394
16	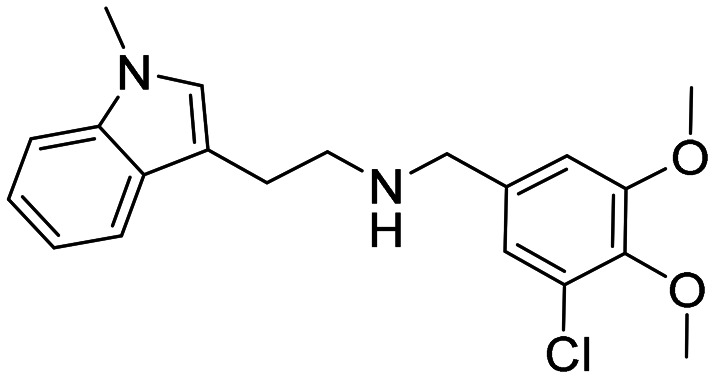	0.04 (0.031–0.05)	n.d.	14 (12–16)	350
17	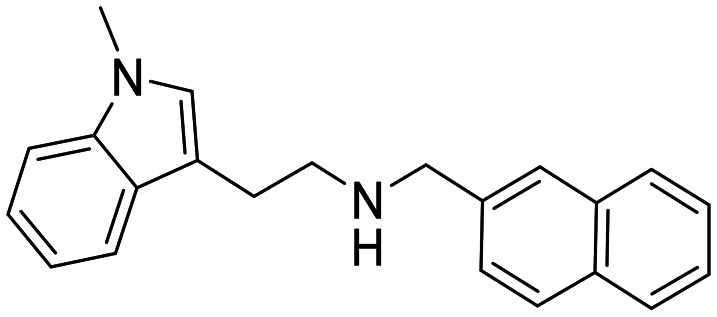	0.51 (0.40–0.65)	n.d.	14 (9.6–21)	27

a Compounds tested as HCl salts unless specified, values given in parentheses = 95% confidence interval; n.d. refers to not determined.

b S.R. = selectivity ratios were calculated by taking the compound's IC_50_ for *h*AChE and dividing by its values for *Ag*AChE1.

**Table 2:** Chemical structures and IC_50_ values of substituted indoles in set B

ID	Structure	*Ag*AChE1	*h*AChE	S.R.^b^
		IC_50_ μM^a^	IC_50_ μM^a^	
18	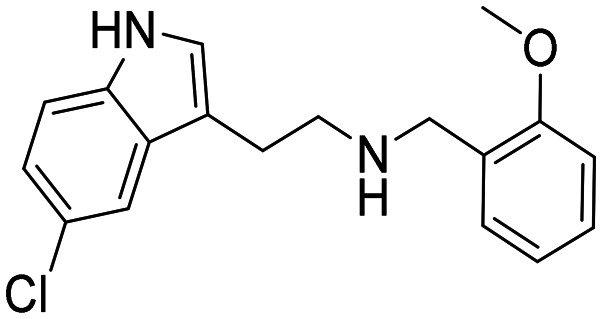	0.4 (0.21–0.60)	62 (54–71)	155
19	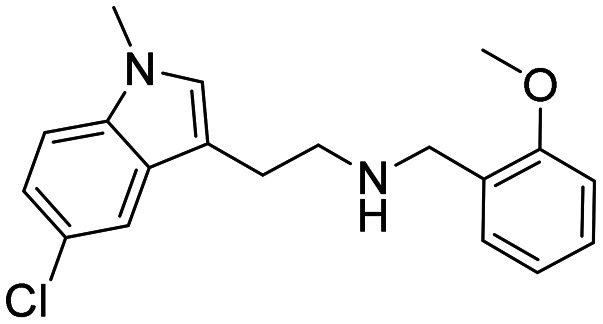	0.07 (0.04–0.1)	4.3 (2.7–6.7)	61
20	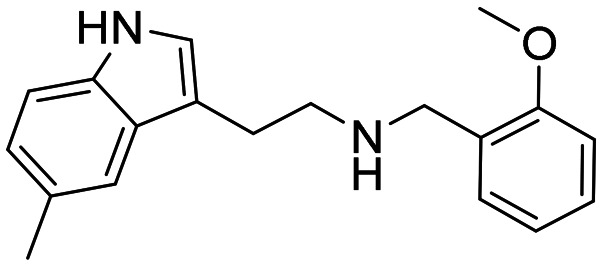	2.7 (2.3–3.1)	93 (80–111)	34
21	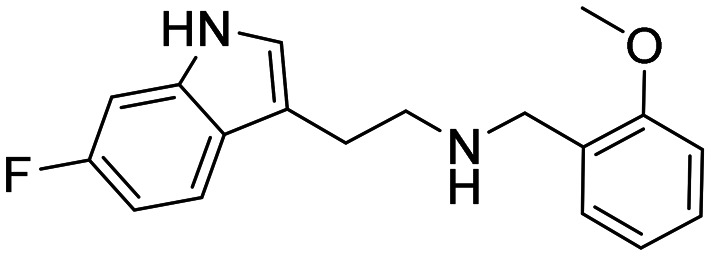	1.6 (1.3–2.0)	53 (46–61)	33
22	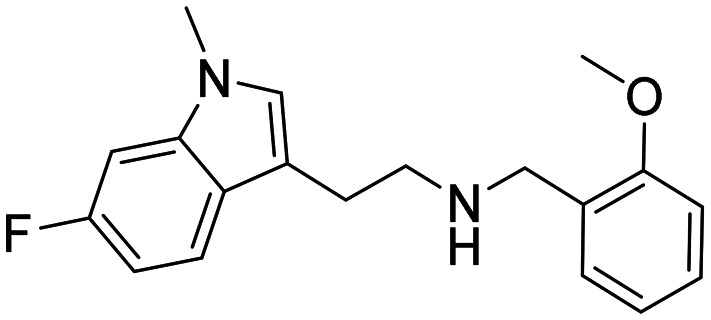	0.2 (0.1–2.8)	3.5 (2.9–4.2)	17
23	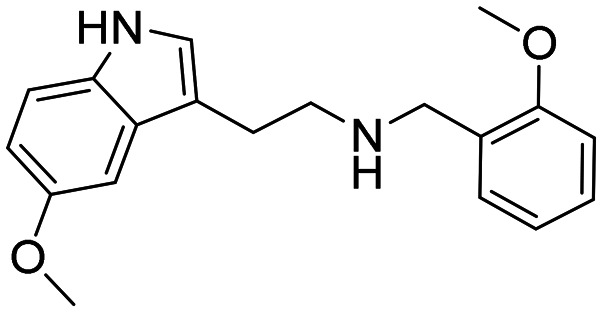	48 (24–110)	156 (128–216)	3
24	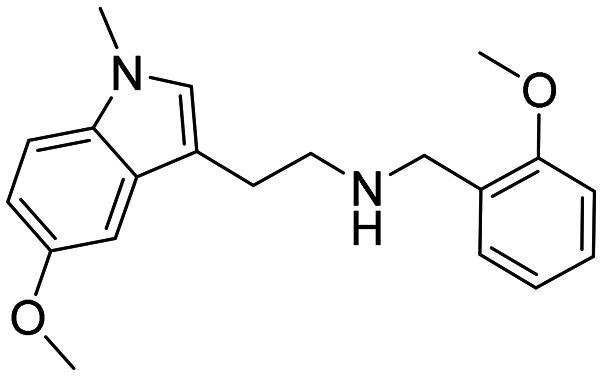	3.2 (1.0–11)	18 (13–27)	6
25	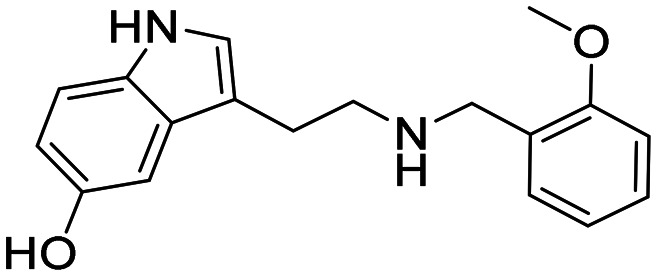	16 (11–23)	93 (52–527)	6
26	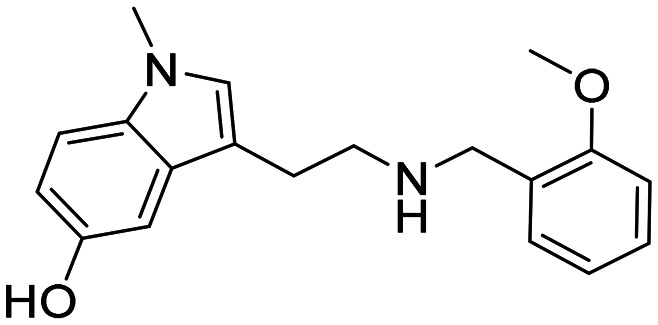	2.1 (1.1–3.9)	17 (10–34)	8

a Compounds tested as HCl salts unless specified, values given in parentheses = 95% confidence interval.

b S.R. = selectivity ratios were calculated by taking the compound's IC_50_ for *h*AChE and dividing by its values for *Ag*AChE1.

**Table 3:** Chemical structures and IC_50_ values of compounds with modified linker in set C

ID	Structure	*Ag*AChE1	*h*AChE	S.R.^b^
		IC_50_ μM^a^	IC_50_ μM^a^	
29	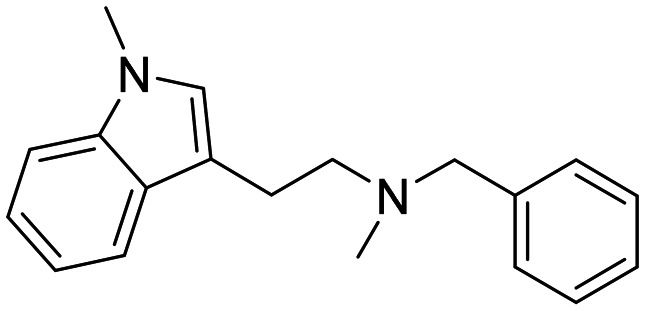	74 (55–112)	>500	>7
30	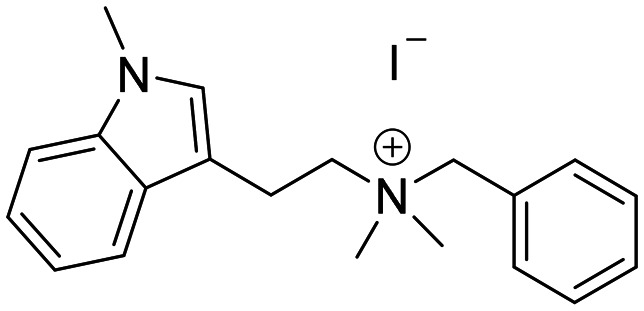	5.5 (3.3–6.7)	5.0 (2.0–22)	0.9
31	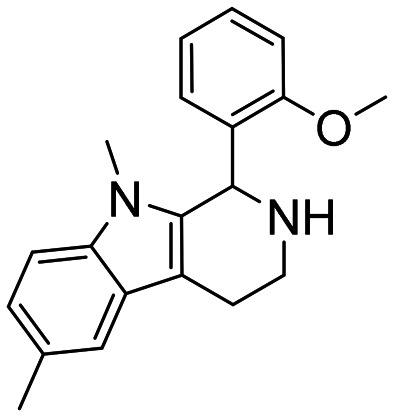	26 (20–33)	>500	>19
32	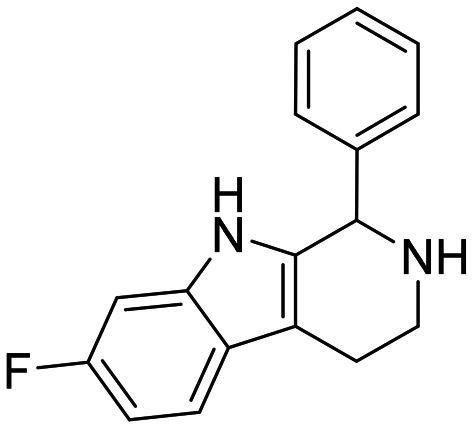	>500	>500	—
33	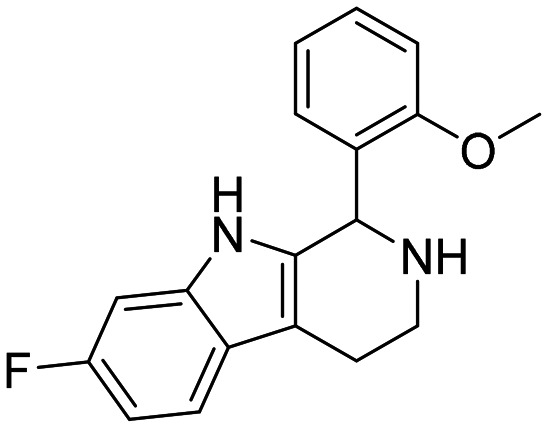	47 (33–78)	>500	>11

a Compounds tested as HCl salts unless specified, values given in parentheses = 95% confidence interval.

b S.R. = selectivity ratios were calculated by taking the compound's IC_50_ for *h*AChE and dividing by its values for *Ag*AChE1.

**Scheme 1:**
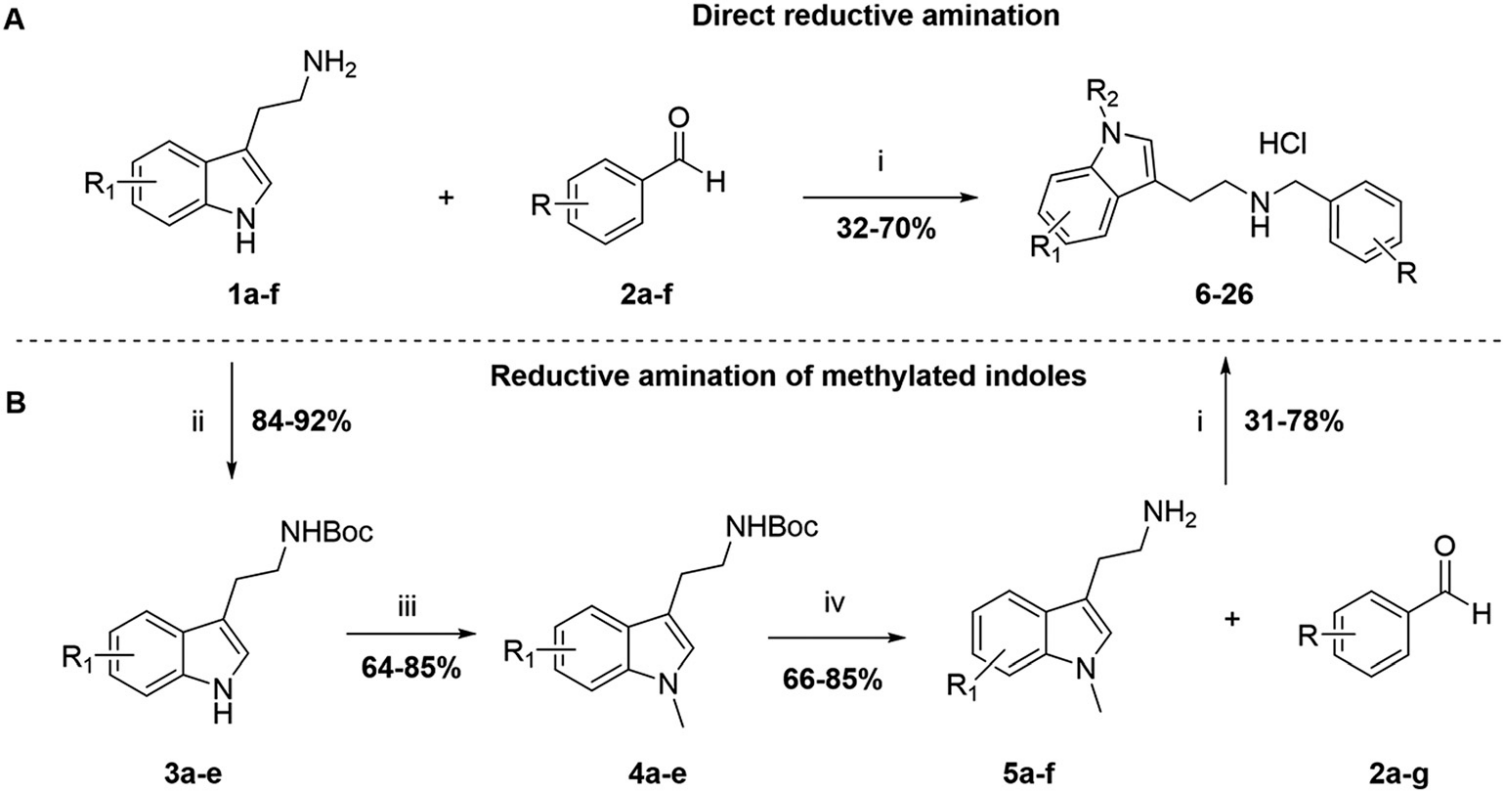
Synthesis of the indole-based compounds 6–26 in sets A and B (Tables 1 and 2). Reagents and conditions: i) EtOH or MeOH, rt or reflux, 12 h; NaBH_4_, 0 °C – rt, 5 h; 2 M HCl in ether; ii) Boc-anhydride, DCM, 0 °C – rt, 12 h. iii) NaH, DMF 0 °C – rt; CH_3_I, rt, 12 h. iv) TFA, DCM 0 °C – rt, 5 h. tryptamines 1a–f and benzaldehydes 2a–f can be found in Fig. S2 and S3.

The derivatives in sets A and B were synthesized following two different pathways (Scheme 1). On the one hand, N–H indole derivatives 6, 8, 10, 12, 15, 18, 20, 21, 23, and 25 (Tables 1 and 2) were obtained through a one-pot direct reductive amination between the commercially available tryptamines 1a–f and benzaldehydes 2a–f (Fig. S2 and S3) in ethanol at room temperature or under reflux conditions, and further reduced by NaBH_4_ or NaCNBH_3_ to afford N–H indole analogues in a 32–70% yield after purification over column chromatography (Scheme 1A). On the other hand, analogues 7, 9, 11, 13, 14, 16, 17, 19, 22, 24, and 26 (Tables 1 and 2) involved the synthesis of the *N*-methylated indole derivatives before performing the reductive amination. The chemical route involved the transitory Boc protection of the primary amine of commercially available tryptamines 1a–f, affording intermediates 3a–e in a 84–92% yield after purification by column chromatography. The *N*-methylation was then performed in the presence of NaH and MeI to provide 4a–e in a 64–85% yield after purification over column chromatography, followed by the Boc-group cleavage in the presence of TFA in DCM to afford 5a–e intermediates with a 66–85% yield without further purification (Scheme 1B). The intermediate 5f was synthesized by a different method, as presented in Scheme S1. The N-alkylated derivatives 5a–f were subjected to a one-pot reductive amination with benzaldehydes 2a–g using NaBH_4_, followed directly by the formation of the corresponding ammonium chloride salts (7, 9, 11, 13, 14, 16, 17, 19, 22, 24, and 26) in the presence of 2 M HCl in diethyl ether. All the final compounds were recrystallized to ≥95% purity from IPA providing a yield of 31–78%.

**Scheme 2:**
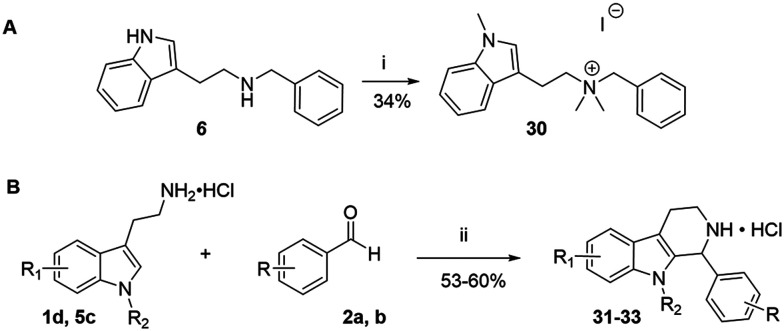
Synthesis of compounds 30 (A) and 31–33 (B) in set C. Reagents and conditions: i) NaH, DMF 0 °C – rt; CH_3_I, rt, 12 h; ii) EtOH, reflux, 12 h; 2 M HCl in ether.

The compounds in set C were designed with modifications in the linker (Table 3), which resulted in different synthetic pathways in order to obtain the five analogues. The dimethylated compound 30 was accessed in one step from compound 6 in the presence of NaH and CH_3_I to form the *N*,*N*-dimethyl ammonium iodide salt in a 34% yield (Scheme 2A). The synthesis of the monomethylated analogue 29 is shown in Scheme S2. The three cyclic compounds 31–33 were synthesized as racemic mixtures through a one-step Pictet–Spengler reaction of hydrochloric salts of tryptamine derivatives 1d and 5c with the aldehydes 2a and 2b (Scheme 2B). All products of set C, were recrystallized to ≥95% purity from IPA after salt formation with a yield of 34–60%.

### Biochemical evaluation of the indole-based compounds

**Fig. 4:**
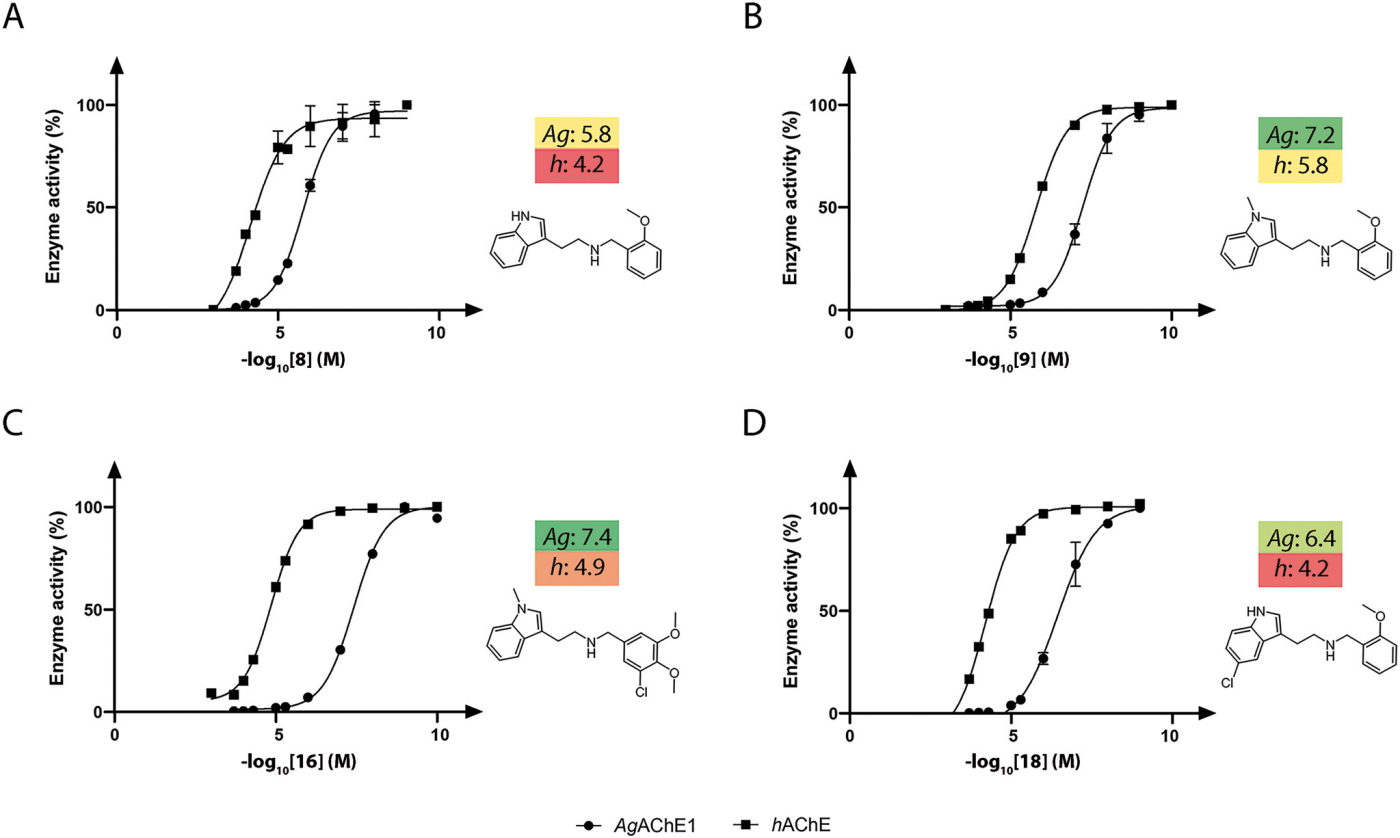
Dose–response curves showing the inhibitory potency of compounds 8 (A), 9 (B), 16 (C), and 18 (D) against *Ag*AChE1 (dots) and *h*AChE (squares). The pIC_50_ values are given beside the dose–response curves and are color coded, with dark green, yellow, and dark red indicating the strongest, medium, and weakest inhibitors, respectively.

The synthesized compounds in sets A–C were investigated for their activity against recombinant *Ag*AChE1 and *h*AChE by determination of their half-maximal inhibitory concentrations (IC_50_) using the Ellman assay (Tables 1–3, Fig. S4). Four analogs were also investigated against *Aa*AChE1 (Table 1), which showed a similar inhibition potency with a Pearson correlation coefficient (*R*^2^) of 0.99 based on the pIC_50_ values. Previous studies have shown that also inhibitors from other chemical classes had similar *in vitro* inhibition profiles against *Ag*AChE1 and *Aa*AChE*.*^14–17^ The indole-based compounds were observed to be potent inhibitors against *Ag*AChE1, while still having a wide range of IC_50_ values, from 0.04 μM (16) up to >500 μM (36). Out of the 26 compounds of all three sets, three (9, 16, and 19) compounds had good inhibition potency with IC_50_ values between 40 and 70 nM. In addition, five more compounds had IC_50_ values in the sub-micromolar range, and only seven inhibitors had IC_50_ values ≥20 μM. In the case of *h*AChE, the compounds were less active; only three compounds had IC_50_ values below 5.0 μM, and 17 compounds had IC_50_ values ≥20 μM. From a selectivity point of view, six compounds were potent and displayed selectivity against *Ag*AChE1 over *h*AChE with selectivity ratios (SR) between 40 and 394 (Tables 1 and 2). Among these potent and selective inhibitors, 8, 15, 16, and 18 from sets A and B are displayed in Fig. 4.

### Structure–activity relationship of inhibition of AChE by the indole-based compounds

A comparison of the IC_50_ values against *Ag*AChE1 for the five pairs of N–H and N-Me indole analogues in set A showed that all compounds increased in potency upon *N*-methylation of the indole moiety (Table 1), ranging from a 3-fold to almost a 30-fold increase in potency. The increase in potency upon *N*-methylation was dependent on the benzyl group attached. The unsubstituted 7 gained the least and the ortho methoxy substituted 9 increased potency the most, in comparison to their N–H analogues (7*vs.*6, and 9*vs.*8). Expansion of the pairwise analysis (N–H *vs.* N-Me) to the analogues in set B showed that also the substituted indole analogues increased their potency against *Ag*AChE1 upon *N*-methylation of the indole. The methoxy substituted indole derivative increased in potency the most (15-fold) from an IC_50_ value of 48 μM for the N–H analogue 23 to 3.2 μM for the N-Me inhibitor 24 (Table 1). The increase in inhibition potency upon *N*-methylation of the indole derivatives was not *Ag*AChE1 specific, since also the IC_50_ values against *h*AChE decreased to a similar extent as for the mosquito enzyme (Tables 1 and 2, Fig. 4A and B).

SAR analysis of the modifications of the benzyl moieties in set A showed that different substituents were tolerated with good inhibition potency against *Ag*AChE1. For example, the *ortho* methoxy- and *para* nitro substituted benzyl analogues 9 and 11 had both submicromolar IC_50_ values. The unsubstituted phenyl ring or non-polar substituents at the *para* position of the ring appeared to be unfavorable for inhibition of *Ag*AChE1. Interestingly, the inhibitors that contained the bulkiest benzyl moiety, the 3-chloro-4,5-dimethoxybenzyl analogues 15 (N–H) and 16 (N-Me), showed remarkable selectivity for *Ag*AChE1 over *h*AChE with selectivity ratios (S.R.) of 394 and 350, respectively (Fig. 4C).

In general, the substitutions at the 5 and 6 positions of the indole moiety of compounds in set B resulted in maintained or decreased inhibition activity against *Ag*AChE1, where the 5-chloro- and 6-fluoro indole analogues gave the best results with low IC_50_ values (Table 2). Still, the 5-chloro N–H indole derivative 18 resulted in improved potency (IC_50_ = 0.4 μM) compared to the unsubstituted analogue 8 (IC_50_ = 1.6 μM). For the indole part of the molecule, polar substituents such as hydroxyl or methoxy appeared to be less favorable for inhibition activity. Again, an interesting observation was made regarding selectivity for *Ag*AChE1 over *h*AChE. The introduced 5-chloro substituent in the indole moiety did not only improve inhibition potency against *Ag*AChE1 (*cf.*18*vs.*8), but also increased the selectivity for *Ag*AChE1 over *h*AChE, with a S.R. of 155 compared to 44 (Fig. 4A and D). A similar trend was also seen for the N-Me analogues 19 (5-Cl) and 9 (5-H), with a S.R. of 61 and 27, respectively.

The ring closure through bond formation between the benzylic carbon and the carbon at position 2 of the indole yielded conformationally restricted analogues of compounds in set B (set C, Table 3). This modification drastically decreased the inhibitory activity, as seen when comparing the cyclized 6-F analogue 33 with the linear 6-F analogue 21 (IC_50_ values of 47 μM *vs.* 1.6 μM). This observation was further strengthened when comparing 31 with 20; despite being *N*-methylated on the indole, the cyclic 31 showed a 10-fold weaker potency than its N–H linear analogue 20. Monomethylation of the secondary amine of the linker (29) resulted in a moderate loss of inhibitory potency compared to analogue 7. Converting 7 (IC_50_ = 11 μM) to the dimethylated quaternary ammonium analogue 30 resulted in a comparable IC_50_ value of 5.5 μM against *Ag*AChE1. This modification led to a complete loss of selectivity for *Ag*AChE1 over *h*AChE, as the introduction of the permanently charged cation in 30 resulted in an IC_50_ value of 5.0 μM against *h*AChE.

### Structure-based analysis of inhibitors in complex with *m*AChE and *Ag*AChE1

**Fig. 5:**
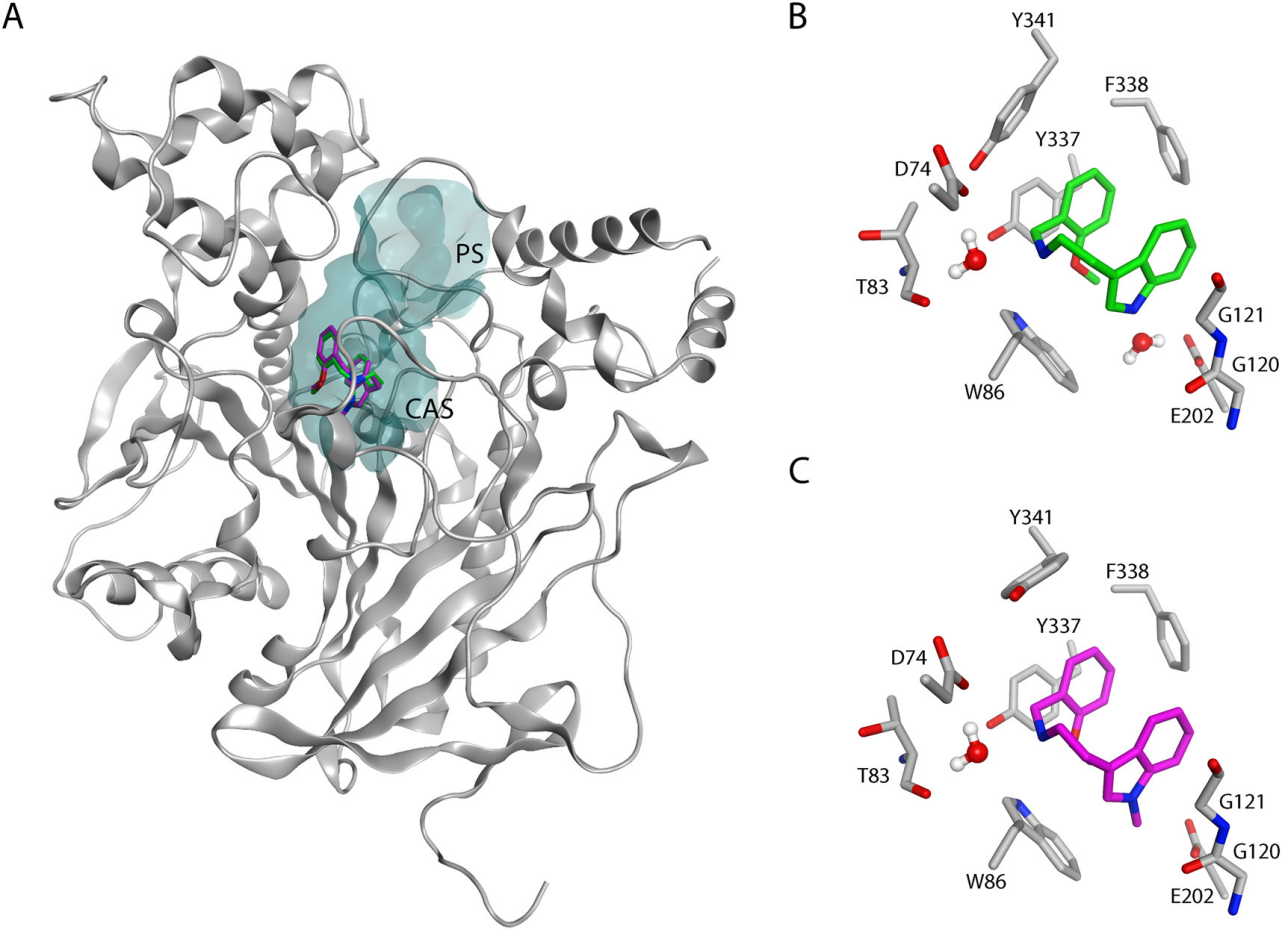
Binding poses of the 2-methoxybenzyl analogues 8 (N–H, green) and 9 (N-Me, magenta) in *m*AChE based on the crystal structures *m*AChE·8 and *m*AChE·9 (PDB: 9SND and 9SNJ). (A) An overview showing the similar binding poses of 8 and 9 located at the bottom of the active site gorge. (B) The binding pose of 8 (N–H, green) with near amino acid residues and two interacting water molecules. (C) The binding pose of 9 (N-Me, magenta) with near amino acid residues and one interacting water molecule. The active site gorge is displayed in grey. Hydrogen atoms have been manually added to the oxygen of water for illustrative purposes.

Using X-ray crystallography, the two 2-methoxybenzyl analogues 8 (N–H) and 9 (N-Me) were structurally determined in complex with *m*AChE (*m*AChE·8 and *m*AChE·9; PDB: 9SND and 9SNJ). The data of the two complexes were of good quality, with resolutions extending to 2.4 Å and 2.3 Å for *m*AChE·8 and *m*AChE·9, respectively (Table S3). The 3D-structures reveal that both compounds bind at the bottom of the gorge, close to the indole of Trp86_m_, with highly similar binding poses (Fig. 5A). The inhibitors have an internal parallel displaced stacking interaction between the phenyl and indole rings with an arene–arene distance of approximately 4 Å, which resulted in a folded compact binding pose. This binding pose is not possible to obtain for the cyclized compounds in set C (31–33), which may explain the substantial decrease in potency of these analogues. Furthermore, the two compounds with mono- and dimethylated secondary amines in the linker (29–30) have presumably different binding poses compared to the compounds in sets A and B, since also these two would not be able to achieve such a compact binding pose without substantial bond strain.

The inhibitors' binding poses have a high shape complementarity with the CAS of *m*AChE, where several amino acid residue–inhibitor contacts are observed (Fig. 5B and C). The 2-methoxybenzyl moieties of 8 and 9 have parallel displaced arene–arene interactions with Tyr337_m_, and edge-to-face arene–arene interactions with Phe338_m_. Furthermore, the indole moieties of 8 and 9 form face arene contacts with the mainchains of Gly120_m_ and Gly121_m_. Although *m*AChE·8 and *m*AChE·9 are structurally very similar, there are distinct differences when studying the water molecules in the CAS. *m*AChE·8 has two water molecules that interact with 8 (Fig. 5B), while *m*AChE·9 only have one inhibitor-interacting water molecule (Fig. 5C). The water molecule in common by the two complexes has hydrogen bonding distances to the secondary amine in the linker and two amino acid residues, Thr83_m_ and Asp74_m_. The unique water molecule in *m*AChE·8 can form a putative hydrogen bond with N–H in the indole and bridge an interaction to Glu202_m_. The *N*-methylation of the indole of 9 displaces the water molecule in the *m*AChE·9 crystal structure compared to *m*AChE·8. This difference allows the N-Me group of 9 to interact with Trp86_m_, which is missing in *m*AChE·8. The additional interaction together with the displacement of the water molecule may account for the substantial increased inhibitory activity of the N-Me indole-based inhibitors compared to their N–H analogues.

*Ag*AChE1 has been shown to have a different shape of the gorge compared to *m*AChE, partly due to structurally different placement of the α-helix lining the gorge.^13^ Tyr337_m_ and Phe338_m_ are located in this α-helix, wherefore the interaction patterns of the inhibitors in complex with *Ag*AChE1 may differ compared to the determined crystal structures. We therefore performed MD simulations of *m*AChE·9 and *Ag*AChE1·9 to elucidate the selectivity profile of the indole-based inhibitor 9.

**Fig. 6:**
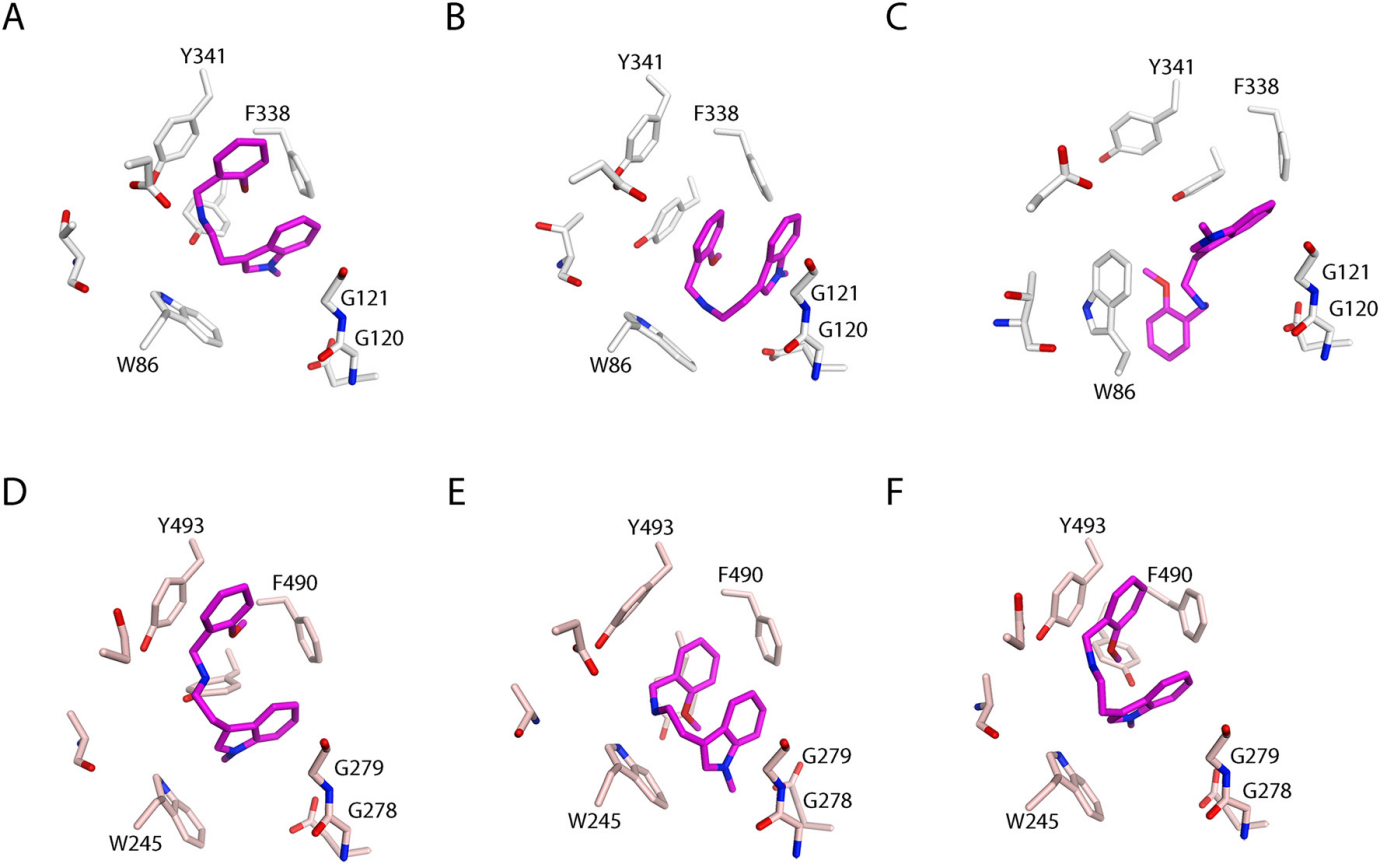
Representative binding poses of 9 in complex with *m*AChE (A–C) and *Ag*AChE1 (D–F) selected based on cluster analysis of the inhibitors' conformations during the MD-simulations. (A–C) The centroid inhibitor conformations of the three largest clusters of *m*AChE·9 with populations of 40%, 26%, and 20% of the analyzed trajectory. (D–F) The centroid inhibitor conformations of the three largest clusters of *Ag*AChE·9 with populations of 35%, 25%, and 24% of the analyzed trajectory. Amino acid residues identified as important for interactions with 9 are highlighted. The tyrosine residue in the center of the residues is Y337/Y489.

Five 100 ns MD simulations were performed for *m*AChE·9 and a prepared model of *Ag*AChE1·9, respectively, with varying initial velocities. According to root-mean-square deviation (RMSD) values, the simulations obtained convergence after 50 ns (Fig. S5). Thus, analysis was performed for the concatenated 50–100 ns simulations. A cluster analysis was performed for the inhibitor conformations over the simulation time, resulting in four and five representative binding modes for *m*AChE·9 and *Ag*AChE1·9, respectively (Fig. S6–S9). The three largest clusters accounted for 86% and 84% of the analyzed trajectory for *m*AChE·9 and *Ag*AChE1·9, respectively, and their centroid binding poses are shown in Fig. 6.

For *m*AChE·9, the compact binding pose at the bottom of the gorge observed for 9 in the X-ray structure was maintained throughout the main part of the simulation (Fig. 6A and B). However, the arene–arene interactions between the methoxy benzyl moiety were occasionally formed to Tyr341_m_ rather than Tyr337_m_ or Phe338_m_ (Fig. 6A). Furthermore, Trp86_m_ was flexible over the simulation time (RMSF = 2.1 Å), and sporadic interactions were formed with the indole of 9 (Fig. 6A–C). For *Ag*AChE1·9, a similar inhibitor conformation was observed over time with some significant differences in the interaction patterns (Fig. 6D–F and S10). Trp245_Ag_ was less flexible compared to Trp86_m_ (RMSF = 0.92 Å) resulting in a more prominent interaction with the indole of 9. Further, the arene–arene interaction between the methoxy benzyl moiety and Tyr493_Ag_ was more populated compared to *m*AChE (Tyr341_m_). The face arene contacts between the indole moiety of 9 and the mainchain of Gly278A_g_/Gly120_m_ and Gly279A_g_/Gly121_m_ were observed in both *m*AChE and *Ag*AChE1, although 9 was positioned closer to these residues in complex with *Ag*AChE1, possibly indicating a more favorable interaction. Overall, 9 had closer contacts to amino acid residues in the CAS of *Ag*AChE1 during the MD-simulations, compared to the simulations of *m*AChE·9.

The occupancy of water molecules within the hydrogen bonding distance to the atoms of 9 (heavy atom distance of <3 Å) was monitored over the simulations of *m*AChE·9 and *Ag*AChE1·9 (Table S4). The analysis revealed low populations of water molecules close to the methoxy benzyl- or indole moieties for both complexes, less than 0.2 waters on average, indicating that the aromatic moieties of the inhibitor are highly shielded in the CAS. The positively charged nitrogen in the linker on the other hand had a higher occupancy of water molecules within 3 Å. Here, *m*AChE·9 displayed a higher water occupancy compared to *Ag*AChE1·9 (0.9 *vs.* 0.4), which may be due to the less tight binding mode of the former complex allowing for closer water contacts.

### Insecticidal effects of the indole-based inhibitors

**Fig. 7:**
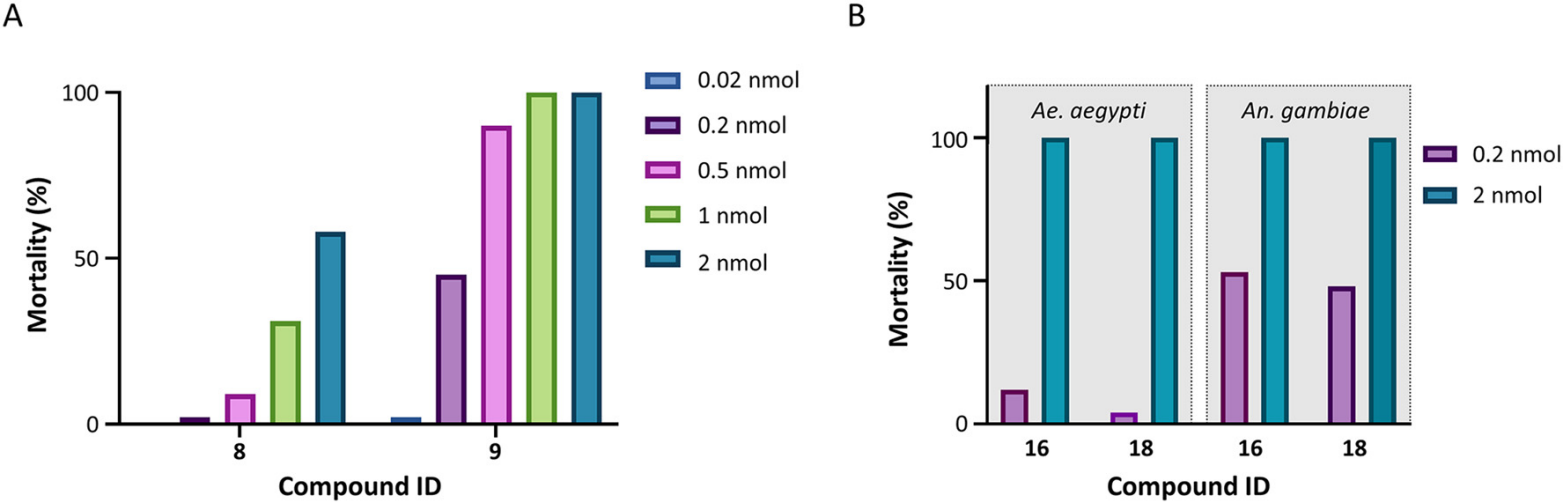
Insecticidal effects of the indole-compounds against mosquitoes using topical application. (A) The insecticidal effect of 8 (N–H) and 9 (N-Me) against mosquitoes of the species *Ae. aegypti* at five doses (0.02, 0.2, 0.5, 1, and 2 nmol per mosquito). (B) The insecticidal effect of 16 and 18 against both mosquito species *Ae. aegypti* and *An. gambiae* at doses of 0.2 and 2 nmol per mosquito.

The insecticidal effect of selected indole-based compounds was investigated against female mosquitoes of the species *Ae. aegypti* and *An. gambiae* (Fig. 4 and 7, Tables S5–S8). The molecular pair of N–H and N-Me indoles with the 2-methoxy-substituted benzyl (8 and 9) was selected to investigate potential *in vivo* differences of the *N*-methylation of the indole moiety, and tested at five doses against *Ae. aegypti* (0.02, 0.2, 0.5, 1, and 2 nmol per mosquito, Fig. 7A). The N-Me indole analogue 9 had an almost 30-fold better *in vitro* inhibitory potency compared to the non-methylated 8 (IC_50_ of 60 nM *vs.* 1600 nM), and a S.R. of 27. Further, two additional compounds were selected; the highly selective inhibitor 3-chloro-4,5-dimethoxybenzyl analogue 16 (N-Me, S.R. = 350), with an IC_50_ value of 40 nM against *Ag*AChE1, and the selective 5-chloro indole analogue 18 (N–H, S.R. = 155) with a 10-fold lower inhibitory potency. Compounds 16 and 18 were tested at two doses against both *Ae. aegypti* and *An. gambiae* (0.2 and 2 nmol per mosquito, Fig. 7B).

The inhibitors 8 and 9 showed both a clear dose-dependent mortality effect against *Ae. aegypti* (Fig. 7A), with approximately a 5-fold stronger insecticidal effect with 9 compared to 8. The *in vivo* results of 16 and 18 against *Ae. aegypti* showed that both compounds had 100% mortality at the highest dose, but less effect with the dose of 0.2 nmol per mosquito (Fig. 7B), resulting in an intermediate insecticidal effect between 9 and 8. The topical application of 16 and 18 on *An. gambiae* resulted in a higher insecticidal effect compared to *Ae. aegypti*, which has been observed before.^15,30,31^ Both compounds showed approximately 50% mortality at the lower dose of 0.2 nmol per mosquito. The observed insecticidal effects were significantly better than previously tested noncovalent AChE1 inhibitors,^15–17^ where compound 9 had an approximate LD_50_ of 59 ng per mosquito against *Ae. aegypti* compared to the best insecticidal activity of 4-thiazolidinones of an approximate LD_50_ of 300 ng per mosquito.^17^ The results further suggest that there is not a clear relation between *in vitro* potency against *Ag*AChE1 and *in vivo* effect. The nanomolar *in vitro* potency was expected to result in an even stronger insecticidal effect; the currently used insecticides propoxur and bendiocarb have reported LD_50_ values of 5.4 ng and 1.8 ng per mosquito.^32^ This discrepancy has been reported before,^15–17^ and may be accounted to the physicochemical properties of the inhibitors. For example, the number of hydrogen bond donors has been proposed to be critical, and should be kept low for a successful insecticide.^33,34^ Thus, the secondary amine of the linker may contribute to the lower *in vivo* insecticidal activity than expected.

## Conclusion

There is a need for new active ingredients to be used as insecticides in vector control of disease-transmitting mosquitoes. Here, indole-based hit compounds from a previous HTS were confirmed as potent and selective inhibitors against AChE1s from the vectors *An. gambiae* and *Ae. aegypti* that spread infectious diseases like malaria and dengue. Three sets of molecules were designed and synthesized to explore the three parts of the molecule: (A) the benzyl-, (B) the indole- and (C) the linker moieties. Methylation of the indole moiety increased the inhibitory potency for all investigated compounds. The most potent compounds 9, 16 and 19 had IC_50_ values of 40–70 nM against *Ag*AChE1 and were selective over *h*AChE. The bulkiest benzyl derivatives 15 and 16 proved to be highly selective for *Ag*AChE1 over *h*AChE with a S.R. of 394 and 350. Compound 9 had a strong *in vivo* insecticidal activity with an approximate LD_50_ of 59 ng per mosquito of species *Ae. aegypti*, which is a 5-fold improvement of previous noncovalent AChE1 inhibitors although still less potent than currently used insecticides. Crystal structures of *m*AChE·8 and *m*AChE·9 showed that the inhibitors form a compact folded binding pose in the lower part of the active site gorge, which could explain the drastic loss of activity for the cyclic derivatives in set C. MD simulations revealed that 9 had closer amino acid residue contacts in *Ag*AChE1 compared to *m*AChE, in particular between the tryptophan residue in the CAS and the indole ring of 9. The binding pose analysis opens up for further medicinal chemistry optimization of the indole inhibitors to improve their *in vivo* insecticidal activity.

## Experimental section

### General aspects of the synthesis of indole-based compounds

All commercially available reagents and solvents were purchased from Enamines, Sigma-Aldrich, Fluorochem, and Fisher Scientific with ≥95% purity, and used without further purification. TLC aluminium sheets coated with silica gel were purchased from Merck. The DMF was dried in a solvent drying system (Glass Contour Solvent Systems, SG Water USA), and stored in sealed RBF, containing 4 Å molecular sieves activated at 180 °C in an oven for more than 48 h before use. All the reactions were carried out under an inert atmosphere in the presence of N_2_ gas. The reaction progress rates were monitored by TLC spot visualization by UV detection (254 nm) or by staining with ninhydrin solution, and with a LC–MS (6130 Quadrupole (Agilent Technologies, USA) mass spectrometer connected to an Agilent 1260 Infinity LC system) analyzer. Synthesized compounds were purified with flash column chromatography (eluents given in brackets) were performed on normal phase silica gel (Merck, 60 Å, 40–63 μm), and on a Biotage Isolera One automated flash chromatography system using Biotage® Sfär Silica, Duo 60 μm silica gel disposable cartridges. Some hydrochloric salt compounds were purified using the crystallization technique with IPA. High-resolution mass spectrometry (HRMS) data were obtained on an Agilent Technologies 6230 TOF LC/MS in ESI mode. NMR spectra were acquired on a Bruker DRX 400 or 600 MHz instrument at 298 K unless otherwise stated. The *δ* values were referenced to the residual solvent signals of CDCl_3_ (7.26 ppm), DMSO-*d*_6_ (2.50 ppm), or CD_3_OD (3.31 ppm) as internal standards for ^1^H, and CDCl_3_ (77.16 ppm), DMSO-*d*_6_ (39.52 ppm), or CD_3_OD (49.00 ppm) for ^13^C. The following abbreviations were used to assign the NMR peaks; s = singlet, d = doublet, t = triplet, q = quartet, bs = broad singlet, dd = doublet of doublets, dt = double of triplets, m = multiplet. Target compounds were ≥95% pure according to ^1^H/^13^C NMR data and LC–MS UV traces.

### Synthesis of building blocks

#### General procedure for the synthesis of 3a–e

The corresponding indoles 1a–f (1 eq.) were dissolved in DCM (2.4 ml mmol^−1^), then TEA (1.5 eq.) and di-*tert*-butyl dicarbonate (1–1.1 eq.) were added at rt and the mixture was stirred for 12 h. After completion of the reaction, the reaction was quenched by the addition of water (50 ml), and extracted with 2 × 150 ml DCM. The combined organic layers were washed with an aqueous saturated NaHCO_3_ solution, and brine. The organic phase was finally dried over Na_2_SO_4_, filtered, and concentrated to obtain the crude compound without further purification.

##### *tert*-Butyl(2-(1*H*-indol-3-yl)ethyl)carbamate (3a)

2-(1*H*-Indol-3-yl)ethan-1-amine 1a (2.0 g, 12.4 mmol) was dissolved in DCM (2.4 ml mmol^−1^) followed by the addition of TEA (2.6 ml, 18.7 mmol) at rt. Di-*tert*-butyl dicarbonate (3.0 g, 13.7 mmol) was then added, and the reaction followed the general procedure. The desired product 3a was obtained as a brown powder (3.0 g, 92% yield). ^1^H NMR (400 MHz, CDCl_3_) *δ* 8.01 (bs, 1H), 7.61 (d, 1H, *J* = 8.8 Hz), 7.38 (d, 1H, *J* = 8.0 Hz), 7.23–7.18 (m, 1H), 7.15–7.10 (m, 1H), 7.00 (s, 1H), 4.62 (bs, 1H), 3.46 (t, 2H, *J* = 7.0 Hz), 2.96 (t, 2H, *J* = 6.8 Hz), 1.43 (s, 9H); ^13^C NMR (100 MHz, CDCl_3_) *δ* 156.1, 136.5, 122.3 (2C), 122.1, 119.6 (2C), 119.0, 111.3, 77.3, 41.0, 28.6 (3C), 25.4.

##### *tert*-Butyl(2-(5-chloro-1*H*-indol-3-yl)ethyl)carbamate (3b)

2-(5-Chloro-1*H*-indol-3-yl)ethan-1-amine 1b (1.2 g, 5.25 mmol) was dissolved in DCM (2.4 ml mmol^−1^) followed by the addition of TEA (1.1 ml, 7.88 mmol) at rt. Di-*tert*-butyl dicarbonate (1.26 g, 5.78 mmol) was then added, and the reaction followed the general procedure. The desired product 3b was obtained as a brown powder (1.4 g, 90% yield). ^1^H NMR (400 MHz, CDCl_3_) *δ* 8.10 (bs, 1H), 7.53–7.52 (m, 1H), 7.24 (d, 1H, *J* = 2.8 Hz), 7.12 (d, 1H, *J* = 8.8 Hz), 7.03 (s, 1H), 4.63 (bs, 1H), 3.40 (t, 2H, *J* = 6.8 Hz), 2.88 (t, 2H, *J* = 6.8 Hz), 1.41 (s, 9H); ^13^C NMR (100 MHz, CDCl_3_) *δ* 156.3, 134.8, 128.7125.4, 123.6, 122.6, 118.5, 113.3, 112.3, 77.4, 41.2, 28.6 (3C), 25.9.

##### *tert*-Butyl(2-(5-methyl-1*H*-indol-3-yl)ethyl)carbamate (3c)

2-(5-Methyl-1*H*-indol-3-yl)ethan-1-amine 1c (0.3 g, 1.42 mmol) was dissolved in DCM (2.4 ml mmol^−1^) followed by the addition of TEA (0.29 ml, 2.13 mmol) at rt. Di-*tert*-butyl dicarbonate (0.34 g, 1.56 mmol) was then added, and the reaction followed the general procedure. The desired product 3c was obtained as a light brown solid (0.35 g, 90% yield). ^1^H NMR (400 MHz, CDCl_3_) *δ* 7.93 (bs, 1H), 7.39 (s, 1H), 7.27–7.25 (m, 1H), 7.04–7.01 (m, 1H), 7.00 (s, 1H), 4.61 (bs, 1H), 3.45 (t, 2H, *J* = 7.2 Hz), 2.93 (t, 2H, *J* = 6.6 Hz), 2.46 (s, 3H), 1.43 (s, 9H); ^13^C (150 MHz, CDCl_3_) *δ* 156.1, 134.9, 128.8, 127.8, 123.9, 122.3, 118.6, 112.8, 111.0, 79.2, 41.1, 28.6 (3 C), 25.9, 21.6.

##### *tert*-Butyl(2-(6-fluoro-1*H*-indol-3-yl)ethyl)carbamate (3d)

2-(6-Fluoro-1*H*-indol-3-yl)ethan-1-amine 1d (0.2 g, 1.20 mmol) was dissolved in DCM (2.4 ml mmol^−1^) followed by the addition of TEA (0.25 ml, 1.80 mmol) at rt. Di-*tert*-butyl dicarbonate (0.26 g, 1.20 mmol) was then added, and the reaction followed the general procedure. The desired product 3d was obtained as a brown solid (0.25 mg, 75% yield). ^1^H NMR (400 MHz, CDCl_3_) *δ* 8.15 (bs, 1H), 7.51–7.47 (m, 1H), 7.06–7.02 (m, 1H), 7.00 (s, 1H), 6.91–6.85 (m, 1H), 4.62 (bs, 1H), 3.44 (t, 2H, *J* = 6.8 Hz), 2.92 (t, 2H, *J* = 7.0 Hz), 1.44 (s, 9H); ^13^C NMR (100 MHz, CDCl_3_) *δ* 160.5 (d, *J*_1C–F_ = 227 Hz), 156.2, 136.4 (d, *J*_3C–F_ = 12 Hz), 124.1, 122.3 (d, *J*_4C–F_ = 3.5 Hz), 119.6 (d, *J*_3C–F_ = 10 Hz), 113.4, 108.3 (d, *J*_2C–F_ = 26 Hz), 95.6 (d, *J*_2C–F_ = 26 Hz), 79.7, 41.2, 28.6 (3C), 26.0.

##### *tert*-Butyl(2-(5-methoxy-1*H*-indol-3-yl)ethyl)carbamate (3e)

2-(5-Methoxy-1*H*-indol-3-yl)ethan-1-amine 1e (1.0 g, 5.25 mmol) was dissolved in DCM (2.4 ml mmol^−1^) followed by the addition of TEA (1.1 ml, 7.88 mmol) at rt. Di-*tert*-butyl dicarbonate (1.3 g, 5.78 mmol) was then added, and the reaction followed the general procedure. The desired product 3e was obtained as a brown solid (1.3 g, 85% yield). ^1^H NMR (400 MHz, CDCl_3_) *δ* 8.08 (bs, 1H), 7.26–7.24 (m, 1H), 7.03 (d, 1H, *J* = 2.4 Hz) 7.00 (s, 1H), 6.86 (dd, 1H, *J*_1_ = 8.8 Hz, *J*_2_ = 2.4 Hz), 4.61 (bs, 1H), 3.87 (s, 3H), 3.45 (t, 2H, *J* = 6.8 Hz), 2.92 (t, 2H, *J* = 7.2 Hz), 1.44 (s, 9H); ^13^C NMR (100 MHz, CDCl_3_) *δ* 156.4, 154.1, 131.7, 127.9, 123.0, 112.9, 112.4, 112.1, 100.7, 79.6, 56.1, 41.3, 28.5 (3C), 26.0.

#### General procedure for the synthesis of 4a–e

NaH 60% in mineral oil (1.1 eq.) was dissolved in dry DMF (1.2 ml mmol^−1^). The corresponding carbamates 3a–e (1.0 eq.) were dissolved in dry DMF (2.5 ml mmol^−1^) and added with a syringe at 0 °C into the NaH solution. After the addition, the reaction was stirred for 30 minutes at rt. CH_3_I (1.1 eq.) was then dropwise added to the mixture at 0 °C, and allowed to stir at rt for 12 hours. After completion, the residue was dissolved in H_2_O (200 ml) and extracted with 3 × 150 ml EtOAc. The combined organic layers were washed with brine and dried over Na_2_SO_4_, filtered, and concentrated to afford an oil as a crude. The purification over column chromatography using EtOAc : heptane (50 : 50) or MeOH : DCM : TEA (10 : 89 : 1) gave the corresponding methylated indoles 4a–e.

##### *tert*-Butyl(2-(1-methyl-1*H*-indol-3-yl)ethyl)carbamate (4a)

NaH 60% in mineral oil (0.5 g, 12.6 mmol) was dissolved in dry DMF (1.2 ml mmol^−1^). *tert*-Butyl(2-(1*H*-indol-3-yl)ethyl)carbamate 3a (3.0 g, 11.5 mmol) was also dissolved in dry DMF (2.5 ml mmol^−1^) and added at 0 °C into the NaH solution. After the addition, the reaction was stirred for 30 minutes at rt. CH_3_I (1.7 g, 12.6 mmol) was added, and the reaction followed the general procedure. The crude was obtained as a brown oil, and then purified over column chromatography using EtOAc : heptane (50 : 50) to provide 4a (2.7 g, 85% yield). ^1^H NMR (400 MHz, CDCl_3_) *δ* 7.59 (d, 1H, *J* = 7.6 Hz), 7.30 (d, 1H, *J* = 7.6 Hz), 7.25–7.21 (m, 1H), 7.13–7.09 (m, 1H), 6.89 (s, 1H), 4.59 (bs, 1H), 3.76 (s, 3H), 3.44 (t, 2H, *J* = 6.8 Hz), 2.94 (t, 2H, *J* = 7.0 Hz), 1.44 (s, 9H); ^13^C NMR (100 MHz, CDCl_3_) *δ* 156.1, 137.2, 128.0, 127.0, 121.8 (2C), 119.1, 119.0, 111.7, 109.4, 79.2, 41.3, 32.8, 28.6 (3C), 25.9.

##### *tert*-Butyl(2-(5-chloro-1-methyl-1*H*-indol-3-yl)ethyl)carbamate (4b)

NaH 60% in mineral oil (0.18 g, 4.66 mmol) was dissolved in dry DMF (1.2 ml mmol^−1^). *tert*-Butyl(2-(5-chloro-1*H*-indol-3-yl)ethyl)carbamate 3b (1.25 g, 4.24 mmol) was also dissolved in dry DMF (2.5 ml mmol^−1^) and added at 0 °C into the NaH solution. After the addition, the reaction was stirred for 30 minutes at rt. CH_3_I (0.66 g, 4.66 mmol) was added, and the reaction followed the general procedure. The crude was obtained as a yellow oil, and then purified over column chromatography using MeOH : DCM : TEA (10 : 89 : 1) to provide 4b (0.87 g, 67% yield). ^1^H NMR (400 MHz, CDCl_3_) *δ* 7.53 (d, 1H, *J* = 1.6 Hz), 7.21–7.15 (m, 2H), 6.91 (bs, 1H), 4.65 (bs, 1H), 3.74 (s, 3H), 3.41 (t, 2H, *J* = 6.8 Hz), 2.89 (t, 2H, *J* = 6.8 Hz), 1.43 (s, 9H); ^13^C NMR (100 MHz, CDCl_3_) δ 163.2, 135.7, 128.4, 125.0 (2 C), 122.1 (2C), 118.5, 110.4 (2C), 77.3, 41.4, 33.0, 28.5 (3C), 25.8.

##### *tert*-Butyl(2-(1,5-dimethyl-1*H*-indol-3-yl)ethyl)carbamate (4c)

NaH 60% in mineral oil (0.07 g, 1.28 mmol) was dissolved in dry DMF (1.2 ml mmol^−1^). *tert*-Butyl(2-(5-methyl-1*H*-indol-3-yl)ethyl)carbamate 3c (0.32 g, 1.16 mmol) was also dissolved in dry DMF (2.5 ml mmol^−1^) and added at 0 °C into the NaH solution. After the addition, the reaction was stirred for 30 minutes at rt. CH_3_I (0.48 g, 1.28 mmol) was added, and the reaction followed the general procedure. The crude was obtained as a yellow oil, and then purified over EtOAc : heptane (50 : 50) to provide 4c (0.3 g, 89% yield). ^1^H NMR (400 MHz, CDCl_3_) *δ* 7.36 (s, 1H), 7.18 (d, 1H, *J* = 8.0 Hz), 7.05 (dd, 1H, *J*_1_ = 7.6 Hz, *J*_2_ = 1.6 Hz), 6.84 (s, 1H), 4.60 (bs, 1H), 3.72 (s, 3H), 3.43 (t, 2H, *J* = 6.8 Hz), 2.91 (t, 2H, *J* = 6.4 Hz), 2.46 (s, 3H), 1.44 (s, 9H); ^13^C NMR (100 MHz, CDCl_3_) δ 156.1, 135.7, 128.2, 127.1, 123.4 (2C), 118.7, 111.1, 109.1, 79.1, 41.1, 32.8, 28.6 (3C), 25.8, 21.6.

##### *tert*-Butyl(2-(6-fluoro-1-methyl-1*H*-indol-3-yl)ethyl)carbamate (4d)

NaH 60% in mineral oil (0.03 g, 0.75 mmol) was dissolved in dry DMF (1.2 ml mmol^−1^). *tert*-Butyl (2-(6-fluoro-1*H*-indol-3-yl)ethyl)carbamate 3d (0.14 g, 0.50 mmol) was also dissolved in dry DMF (2.5 ml mmol^−1^) and added at 0 °C into the NaH solution. After the addition, the reaction was stirred for 30 minutes at rt. CH_3_I (0.10 g, 0.75 mmol) was added, and the reaction followed the general procedure. The crude was obtained as a yellow oil, and then purified over column chromatography using MeOH : DCM : TEA (10 : 89 : 1) to provide 4d (0.1 g, 68% yield). ^1^H NMR (400 MHz, CDCl_3_) *δ* 7.50–7.46 (m, 1H), 6.95 (dd, 1H, *J*_1_ = 10 Hz, *J*_2_ = 2.0 Hz), 6.89–6.83 (m, 2H), 4.59 (s, 1H), 3.70 (s, 3H), 3.44–3.41 (m, 2H), 2.91 (t, 2H, *J* = 7.0 Hz), 1.44 (s, 9H); ^13^C NMR (100 MHz, CDCl_3_) *δ* 160.0 (d, *J*_1C–F_ = 243 Hz), 156.0, 137.3 (d, *J*_3C–F_ = 10 Hz), 127.1, 124.5, 119.6 (d, *J*_3C–F_ = 10 Hz), 112.0, 107.7 (d, *J*_2C–F_ = 26 Hz), 95.7 (d, *J*_2C–F_ = 26 Hz), 79.3, 41.1, 32.9, 28.5 (3C), 25.9.

##### *tert*-Butyl(2-(5-methoxy-1-methyl-1*H*-indol-3-yl)ethyl)carbamate (4e)

NaH 60% in mineral oil (0.25 g, 6.19 mmol) was dissolved in dry DMF (1.2 ml mmol^−1^). *tert*-Butyl(2-(5-methoxy-1*H*-indol-3-yl)ethyl)carbamate 3e (1.2 g, 4.13 mmol) was also dissolved in dry DMF (2.5 ml mmol^−1^) and added at 0 °C into the NaH solution. After the addition, the reaction was stirred for 30 minutes at rt. CH_3_I (1.9 ml, 6.2 mmol) was added, and the reaction followed the general procedure. The crude was obtained as a yellow oil, and then purified over column chromatography using MeOH : DCM : TEA (10 : 89 : 1) to provide 4e (1.0 g, 80% yield) ^1^H NMR (400 MHz, CDCl_3_) *δ* 7.20 (d, 1H, *J* = 8.8 Hz), 7.02 (d, 1H, *J* = 2.4 Hz), 6.90–6.83 (m, 2H), 4.74 (bs, 1H), 3.87 (s, 3H), 3.73 (s, 3H), 3.43 (t, 2H, *J* = 6.6 Hz), 2.90 (t, 2H, *J* = 6.6 Hz), 1.43 (s, 9H); ^13^C NMR (100 MHz, CDCl_3_) *δ* 156.2, 153.8, 132.7, 128.2, 127.6, 112.2, 110.2, 110.0, 100.9, 79.4, 56.3, 41.3, 33.0, 28.9 (3C), 26.1.

##### *tert*-Butyl methyl(2-(1-methyl-1*H*-indol-3-yl)ethyl)carbamate (27)

NaH 60% in mineral oil (174 mg, 4.37 mmol) was dissolved in dry DMF (1.2 ml mmol^−1^). Compound *tert*-butyl(2-(1-methyl-1*H*-indol-3-yl)ethyl)carbamate 4a (159 mg, 0.54 mmol) was also dissolved in dry DMF (2.5 ml mmol^−1^) and was added slowly into the solution of NaH at 0 °C, and the reaction was cooled down to 0 °C and CH_3_I (341 μl, 5.46 mmol) was added dropwise to the reaction, and after completion of addition, the mixture was stirred at rt for 30 minutes, and then 5 hours at 80 °C. After completion, the crude was dissolved in H_2_O (200 ml) and extracted with 3 × 50 ml EtOAc, combined organic layers were washed with brine dried over Na_2_SO_4_, filtered and concentrated, and the obtained brown sticky crude was purified over column chromatography with EtOAc : heptane (50 : 50) to provide 27 (100 mg, 63.4% yield). ^1^H NMR (400 MHz, CDCl_3_) *inter alia δ* 7.76 (d, 1H, *J* = 8.8 Hz), 7.44 (d, 1H, *J* = 8.0 Hz), 7.40–7.36 (m, 1H), 7.29–7.25 (m, 1H), 7.04–7.00 (m, 1H), 3.88 (s, 3H), 3.64 (m, 2H), 3.10–3.09 (m, 2H), 3.03 (s, 3H), 1.42 (s, 9H); ^13^C NMR (100 MHz, CDCl_3_) *δ* 155.9, 137.2, 126.8 (2C), 121.7, 119.0, 118.9, 112.0, 109.3, 79.3, 49.9, 37.5, 34.4, 28.5 (3C) 23.3.

#### Common procedure for the synthesis of 5a–f

The corresponding carbamates 4a–4e or 27 (1.0 eq.) were dissolved in DCM (2.5 ml mmol^−1^), then TFA (30 eq.) was added to the reaction mixture at rt and the solution was stirred for 1 h. After completion, the reaction was quenched with a solution of saturated NaHCO_3_, the aqueous layer was extracted with 3 × 150 ml DCM, and the combined organic layers were washed with brine and dried over Na_2_SO_4_, filtered, and concentrated to give the corresponding methylated indoles 5a–e.

##### 2-(1-Methyl-1*H*-indol-3-yl)ethan-1-amine (5a)

*tert*-Butyl(2-(1-methyl-1*H*-indol-3-yl)ethyl)carbamate 4a (2.6 g, 9.73 mmol) was dissolved in DCM (2.5 ml mmol^−1^), then TFA (1.9 ml, 25 mmol) was added at rt, and the reaction followed the general procedure. The desired compound was obtained as a powder 5a (1.2 g, 71% yield). ^1^H NMR (400 MHz, DMSO-*d*_6_) *δ* 8.02 (bs, 3H), 7.58 (d, 1H, *J* = 8.0 Hz), 7.42 (d, 1H, *J* = 8.0 Hz), 7.23 (s, 1H), 7.17 (t, 1H, *J* = 7.6 Hz), 7.06 (t, 1H, *J* = 7.4 Hz), 3.75 (s, 3H), 3.04–3.01 (m, 4H); ^13^C NMR (100 MHz, DMSO-*d*_6_) *δ* 136.7, 127.8, 127.1, 121.3, 118.6, 118.4, 109.8, 108.8, 40.6, 32.3, 23.0.

##### 2-(5-Chloro-1-methyl-1*H*-indol-3-yl)ethan-1-amine (5b)

*tert*-Butyl(2-(5-chloro-1-methyl-1*H*-indol-3-yl)ethyl)carbamate 4b (0.85 g, 2.75 mmol) was dissolved in DCM (2.5 ml mmol^−1^), then TFA (6.3 ml, 82.5 mmol) was added to the reaction at rt, and the reaction followed the general procedure. The desired compound was obtained as a brown powder 5b (0.49 g, 85% yield) ^1^H NMR (400 MHz, CDCl_3_) *δ* 7.54 (s, 1H), 7.16 (m, 2H), 6.92 (s, 1H), 3.72 (s, 3H), 3.01 (s, 2H), 2.87 (s, 2H), 2.77 (s, 2H); ^13^C NMR (100 MHz, CDCl_3_) δ 135.7, 128.5 (2C), 124.8, 122.0, 118.6, 111.4, 110.4, 42.1, 32.9, 28.2.

##### 2-(1,5-Dimethyl-1*H*-indol-3-yl)ethan-1-amine (5c)

*tert*-Butyl(2-(1,5-dimethyl-1*H*-indol-3-yl)ethyl)carbamate 4c (0.29 g, 1.00 mmol) was dissolved in DCM (2.5 ml mmol^−1^), then TFA (2.3 ml) was added to the reaction at rt, and the reaction followed the general procedure. The desired compound was obtained as a brown powder 5c (0.17 g, 89% yield). ^1^H NMR (400 MHz, CDCl_3_) *δ* 7.32 (s, 1H), 7.14 (d, 1H, *J* = 8.0 Hz), 7.00 (d, 1H, *J* = 8.4 Hz), 6.88 (bs, 1H), 5.92 (s, 2H), 3.66 (s, 3H), 3.16–3.05 (m, 4H), 2.42 (s, 3H); ^13^C NMR (100 MHz, CDCl_3_) *δ* 135.7, 128.4, 128.1, 127.6, 123.7, 118.3, 109.3, 108.1, 40.5, 32.8, 24.1, 21.5.

##### 2-(6-Fluoro-1-methyl-1*H*-indol-3-yl)ethan-1-amine (5d)

*tert*-Butyl(2-(6-fluoro-1-methyl-1*H*-indol-3-yl)ethyl)carbamate 5d (0.12 g, 0.41 mmol) was dissolved in DCM (2.5 ml mmol^−1^), then TFA (0.9 ml) was added to the reaction at rt, and the reaction followed the general procedure. The desired compound was obtained as a brown powder 5d (60 mg, 76% yield) ^1^H NMR (400 MHz, DMSO-d_6_) *δ* 7.86 (bs, 2H), 7.57–7.53 (m, 1H), 7.30–7.27 (m, 1H), 7.22 (s, 1H), 6.91 (t, 1H, *J* = 9.0 Hz), 3.72 (s, 3H), 3.07–3.01 (m, 2H), 2.99–2.93 (m, 2H); ^13^C NMR (100 MHz, DMSO-*d*_6_) *δ* 159.2 (d, *J*_1C–F_ = 240 Hz), 136.8 (d, *J*_3C–F_ = 10 Hz), 128.4 (d, *J*_4C–F_ = 3.1 Hz), 123.9, 119.5 (d, *J*_3C–F_ = 10 Hz), 109.2, 107.0 (d, *J*_2C–F_ = 24 Hz), 96.2 (d, *J*_2C–F_ = 24 Hz), 48.6, 32.5, 22.8.

##### 2-(5-Methoxy-1-methyl-1*H*-indol-3-yl)ethan-1-amine (5e)

*tert*-Butyl(2-(5-methoxy-1-methyl-1*H*-indol-3-yl)ethyl)carbamate 4e (1.0 g, 3.28 mmol) was dissolved in DCM (2.5 ml mmol^−1^), then TFA (7.5 ml) was added to the reaction at rt, and the reaction followed the general procedure. The desired compound was obtained as a brown powder 5e (0.55 g, 82% yield). ^1^H NMR (400 MHz, DMSO-*d*_6_) *δ* 7.97 (bs, 2H), 7.30 (d, 1H, *J* = 8.8 Hz), 7.16 (s, 1H), 7.08 (d, 1H, *J* = 2.0 Hz), 6.83–6.78 (m, 1H), 3.72 (s, 3H), 3.70 (s, 3H), 3.06–2.91 (m, 4H); ^13^C NMR (100 MHz, DMSO-*d*_6_) *δ* 153.3, 132.1, 128.3, 127.5, 111.3, 110.5, 108.3, 100.4, 55.5, 40.1, 32.5, 22.9.

##### 3-(2-Aminoethyl)-1-methyl-1*H*-indol-5-ol (5f)

2-(5-Methoxy-1-methyl-1*H*-indol-3-yl)ethan-1-amine 5e (0.27 g, 1.32 mmol) was dissolved in DCM (2.5 ml mmol^−1^) and allowed to stir and cool down until −70 °C. After 10 minutes, BBr_3_ (0.66 g, 2.64 mmol) was added slowly to the reaction mixture. After the addition was complete, the reaction was allowed to reach 0 °C and stirred for 4 hours. The reaction mixture was diluted with dichloromethane (25 mL), washed with water (2 × 10 mL) and brine (20 mL), and dried over anhydrous Na_2_SO_4_, and the solvent was removed and purified by column chromatography using 20% ethyl acetate in petroleum ether as the eluent to provide the desired compound 5f as a brown powder (0.24 g, 67% yield). ^1^H NMR (400 MHz, CD_3_OD) *δ* 7.18 (d, 1H, *J* = 8.8 Hz), 7.07 (s, 1H), 6.97 (d, 1H, *J* = 2.0 Hz), 6.76 (dd, 1H, *J*_1_ = 8.2 Hz, *J*_2_ = 2.6 Hz), 3.73 (s, 3H), 3.20 (t, 2H, *J* = 6.2 Hz), 3.06 (t, 2H, *J* = 6.8 Hz); ^13^C NMR (100 MHz, CD_3_OD) *δ* 152.0, 133.9, 129.4 (2C), 112.7, 111.2, 108.6, 103.4, 41.0, 32.9, 24.4.

##### *N*-Methyl-2-(1-methyl-1*H*-indol-3-yl)ethan-1-amine (28, Scheme S3)

*tert*-Butyl methyl(2-(1-methyl-1*H*-indol-3-yl)ethyl)carbamate 27 (0.1 g, 0.32 mmol) was dissolved in DCM (2.5 ml mmol^−1^), then TFA (0.8 ml) was added to the reaction at rt, and the reaction followed the general procedure. The desired compound was obtained as a brown powder 28 (0.05 g, 76% yield). ^1^H NMR (400 MHz, CDCl_3_) *δ* 7.59 (d, 1H, *J* = 8.0 Hz), 7.29 (d, 1H, *J* = 8.4 Hz), 7.24 (t, 1H, *J* = 7.6 Hz), 7.11 (t, 1H, *J* = 7.4 Hz), 6.92 (s, 1H), 6.31 (bs, 1H), 3.73 (s, 3H), 3.12–3.09 (m, 4H), 2.54 (s, 3H); ^13^C NMR (100 MHz, CDCl_3_) *δ* 137.2, 127.5, 127.3, 122.0, 119.2, 118.8, 110.1, 109.5, 50.9, 34.4, 32.7, 23.6.

#### General procedure for the synthesis of indoles 6–17

The corresponding tryptamines 5a–f (1.0 eq.) and the corresponding benzaldehydes 2a–f (1.1 eq.) were dissolved in EtOH or MeOH (3 ml mmol^−1^) and allowed to reflux for 12 h. The reaction mixture was then cooled down to rt and NaBH_4_ (1.5 eq.) was added and allowed to stir at rt for 1 h. After completion, the reaction was quenched with a saturated solution of NaHCO_3_ and EtOAc was added. The water phase was extracted three times with EtOAc (3 × 150 ml), and the combined organic layers were washed once with brine, dried over Na_2_SO_4_, filtered, and concentrated. The crude was dissolved in EtOAc (1 ml) and treated with 2 M HCl (1.0 eq.) in diethyl ether at 0 °C, filtered, and then recrystallized from IPA to obtain the desired compound as a white powder.

##### *N*-Benzyl-2-(1*H*-indol-3-yl)ethan-1-amine hydrochloride (6)

2-(1*H*-Indol-3-yl)ethan-1-amine 1a (150 mg, 0.94 mmol) and benzaldehyde 2a (109 mg, 1.03 mmol) were dissolved in EtOH (3 ml mmol^−1^) and allowed to reflux at 90 °C for 12 h. The reaction mixture was cooled down to rt and NaBH_4_ (53.1 mg, 1.40 mmol) was added, and the reaction was carried out according to the general procedure to afford compound 6 (170 mg, 63% yield). ^1^H NMR (400 MHz, DMSO-*d*_6_) *δ* 11.0 (bs, 1H), 9.28 (bs, 2H), 7.58–7.55 (m, 3H), 7.47–7.42 (m, 3H), 7.37 (d, 1H, *J* = 6.8 Hz), 7.24–7.20 (m, 1H), 7.12–6.99 (m, 2H), 4.20 (s, 2H), 3.18–3.13 (m, 4H); ^13^C NMR (100 MHz, DMSO-*d*_6_) *δ* 136.3, 132.1, 130.0 (2C), 128.9, 128.7 (2C), 126.7, 123.3, 121.2, 118.5, 118.1, 111.6, 109.3, 49.8, 47.1, 21.6. HRMS *m*/*z* [M + H]^+^ calcd. 251.1543, found 251.1511.

##### *N*-Benzyl-2-(1-methyl-1*H*-indol-3-yl)ethan-1-amine hydrochloride (7)

2-(1-Methyl-1*H*-indol-3-yl)ethan-1-amine 5a (100 mg, 0.57 mmol) and benzaldehyde 2a (67 mg, 0.63 mmol) were dissolved in EtOH (3 ml mmol^−1^) and allowed to reflux for 12 h. Then, NaBH_4_ (32.0 mg, 0.86 mmol) was added at rt, and the reaction was carried out according to the general procedure to afford compound 7 (92 mg, 60% yield). ^1^H NMR (600 MHz, DMSO-d_6_) *δ* 9.13 (bs, 1H), 7.57–7.54 (m, 3H), 7.45–7.40 (m, 4H), 7.21 (s, 1H), 7.17 (t, 1H, *J* = 7.5 Hz), 7.05 (t, 1H, *J* = 7.5 Hz), 4.20 (s, 2H), 3.74 (s, 3H), 3.15 (t, 2H, *J* = 7.5 Hz), 3.08 (t, 2H, *J* = 7.2 Hz); ^13^C NMR (150 MHz, DMSO-d_6_) *δ* 136.7, 132.3, 130.0 (2C), 129.0, 128.7 (2C), 127.7, 127.0, 121.4, 118.6, 118.4, 109.8, 108.6, 50.0, 47.2, 32.3, 21.6. HRMS *m*/*z* [M + H]^+^ calcd. 265.1700, found 265.1675.

##### 2-(1*H*-Indol-3-yl)-*N*-(2-methoxybenzyl)ethan-1-amine hydrochloride (8)

2-(1*H*-Indol-3-yl)ethan-1-amine 1a (50 mg, 0.31 mmol) and 2-methoxybenzaldehyde 2b (52 mg, 0.3 mmol) were dissolved in MeOH (3 ml mmol^−1^) and the mixture was stirred at rt for 12 h. NaBH_4_ (20 mg, 0.53 mmol) was then added, and the reaction was carried out according to the general procedure to afford compound 8 (77 mg, 78% yield). ^1^H NMR (400 MHz, DMSO-*d*_6_) *δ* 11.0 (s, 1H), 9.20 (bs, 2H), 7.56 (d, 1H, *J* = 7.6 Hz), 7.51 (d, 1H, *J* = 7.6 Hz), 7.44–7.40 (m, 1H), 7.38–7.36 (m, 1H), 7.23 (d, 1H, *J* = 2.0 Hz), 7.11–7.07 (m, 2H), 7.02–6.98 (m, 2H), 4.18–4.14 (m, 2H), 3.82 (s, 3H), 3.15–3.13 (m, 4H); ^13^C NMR (100 MHz, DMSO-*d*_6_) *δ* 157.5, 136.3, 131.5, 130.8, 126.7, 123.3, 121.2, 120.4, 119.8, 118.5, 118.2, 111.6, 111.1, 109.3, 55.6, 47.0, 44.9, 21.5 HRMS *m*/*z* [M + H]^+^ calcd. 281.1649, found 281.1669.

##### *N*-(2-Methoxybenzyl)-2-(1-methyl-1*H*-indol-3-yl)ethan-1-amine hydrochloride (9)

2-(1-Methyl-1*H*-indol-3-yl)ethan-1-amine 5b (343 mg, 1.96 mmol) and 2-methoxybenzaldehyde 2b (295 mg, 2.16 mmol) were dissolved in EtOH (3 ml mmol^−1^) and allowed to reflux for 12 h. NaBH_4_ (112 mg, 2.95 mmol) was then added at rt, and the reaction was carried out according to the general procedure to afford compound 9 (302 mg, 47% yield). ^1^H NMR (400 MHz, DMSO-*d*_6_) *δ* 9.25 (bs, 2H), 7.58 (d, 1H, *J* = 7.6 Hz), 7.53–7.50 (m, 1H), 7.44–7.40 (m, 2H), 7.21 (s, 1H), 7.18–7.14 (m, 1H), 7.09–6.98 (m, 3H), 4.17–4.14 (m, 2H), 3.82 (s, 3H), 3.74 (s, 3H), 3.17–3.09 (m, 4H). ^13^C NMR (100 MHz, DMSO-d_6_) *δ* 157.5, 136.7, 131.5, 130.7, 127.6, 127.0, 121.3, 120.4, 119.8, 118.6, 118.4, 111.1, 109.8, 108.7, 55.6, 46.9, 44.8, 32.3, 21.3. HRMS *m*/*z* [M + H]^+^ calcd. 295.1766, found 295.1762.

##### 2-(1*H*-Indol-3-yl)-*N*-(4-nitrobenzyl)ethan-1-amine hydrochloride (10)

2-(1*H*-Indol-3-yl)ethan-1-amine 1a (50 mg, 0.31 mmol) and 4-nitrobenzaldehyde 2c (47 mg, 0.31 mmol) were dissolved in MeOH (3 ml mmol^−1^) and the mixture was stirred at rt for 12 h. NaBH_4_ (20 mg, 0.53 mmol) was then added, and the reaction was carried out according to the general procedure to afford compound 10 (82 mg, 80% yield). ^1^H NMR (400 MHz, DMSO-*d*_6_) *δ* 11.0 (s, 1H), 9.53 (bs, 2H), 8.31 (d, 2H, *J* = 8.8 Hz), 7.86 (d, 2H, *J* = 8.8 Hz), 7.58 (d, 1H, *J* = 8.0 Hz), 7.37 (d, 1H, *J* = 8.0 Hz), 7.24 (d, 1H, *J* = 2.0 Hz), 7.10 (t, 1H, *J* = 7.4 Hz), 7.01 (t, 1H, *J* = 7.8 Hz), 4.36 (s, 2H), 3.23–3.18 (m, 2H), 3.15–3.11 (m, 2H); ^13^C NMR (100 MHz, DMSO-d_6_) *δ* 147.7, 139.7, 136.4, 131.3 (2C), 126.7, 123.6 (2C), 123.4, 121.2, 118.5, 118.1, 111.6, 109.2, 48.9, 47.2, 21.5. HRMS *m*/*z* [M + H]^+^ calcd. 296.1394, found 296.1379.

##### 2-(1-Methyl-1*H*-indol-3-yl)-*N*-(4-nitrobenzyl)ethan-1-amine hydrochloride (11)

2-(1-Methyl-1*H*-indol-3-yl)ethan-1-amine 5a (100 mg, 0.57 mmol) and 4-nitrobenzaldehyde 2c (95 mg, 0.53 mmol) were dissolved in EtOH (3 ml mmol^−1^) and the mixture was stirred at rt for 12 h. NaBH_4_ (54 mg, 0.86 mmol) was then added, and the reaction was carried out according to the general procedure to afford compound 11 (30 mg, 17% yield). ^1^H NMR (600 MHz, DMSO-*d*_6_) *δ* 9.33 (bs, 2H), 8.32 (d, 2H, *J* = 8.4 Hz), 7.84 (d, 2H, *J* = 8.4 Hz), 7.59 (d, 1H, *J* = 7.6 Hz), 7.42 (d, 1H, *J* = 8.4 Hz), 7.23 (s, 1H), 7.18 (t, 1H, *J* = 7.4 Hz), 7.06 (t, 1H, *J* = 7.6 Hz), 4.37 (s, 2H), 3.75 (s, 3H), 3.24–3.05 (m, 4H); ^13^C NMR (150 MHz, DMSO-d_6_) *δ* 147.7, 139.6, 136.7, 131.3 (2C), 127.7, 127.0, 123.6 (2C), 121.4, 118.6, 118.4, 109.8, 108.6, 48.9, 47.2, 32.3, 21.5. HRMS *m*/*z* [M + H]^+^ calcd. 310.1551, found 310.1546.

##### *N*-(4-Chlorobenzyl)-2-(1*H*-indol-3-yl)ethan-1-amine hydrochloride (12)

2-(1*H*-Indol-3-yl)ethan-1-amine 1a (120 mg, 0.74 mmol) and 4-chlorobenzaldehyde 2d (119 mg, 0.84 mmol) were dissolved in EtOH (3 ml mmol^−1^) and allowed to reflux for 12 h. NaBH_4_ (42 mg, 1.12 mmol) was then added, and the reaction was carried out according to the general procedure to afford compound 12 (142 mg, 62% yield). ^1^H NMR (400 MHz, DMSO-*d*_6_) *δ* 11.0 (s, 1H), 9.17 (bs, 2H), 7.59–7.50 (m, 4H), 7.36 (d, 1H, *J* = 8.0 Hz), 7.21 (d, 1H, *J* = 1.8 Hz), 7.13–7.07 (m, 1H), 7.04–6.98 (m, 1H), 4.19 (s, 2H), 3.16–3.13 (m, 2H), 3.10–3.08 (m 2H); ^13^C NMR (100 MHz, DMSO-d_6_) *δ* 136.3, 133.7, 132.0 (2C), 131.3, 128.7 (2C), 126.7, 123.3, 121.2, 118.5, 118.1, 111.6, 109.3, 49.1, 47.0, 21.8. HRMS *m*/*z* [M + H]^+^ calcd. 285.1154, found 285.1135.

##### *N*-(4-Chlorobenzyl)-2-(1-methyl-1*H*-indol-3-yl)ethan-1-amine hydrochloride (13)

2-(1-Methyl-1*H*-indol-3-yl)ethan-1-amine 5a (100 mg, 0.57 mmol) and 4-chlorobenzaldehyde 2d (89 mg, 0.63 mmol) were dissolved in EtOH (3 ml mmol^−1^) and the mixture was stirred at rt for 12 h. NaBH_4_ (32 mg, 0.86 mmol) was then added, and the reaction was carried out according to the general procedure to afford compound 13 (37 mg, 20% yield). ^1^H NMR (600 MHz, DMSO-*d*_6_) *δ* 9.61 (bs, 2H), 7.63 (d, 2H, *J* = 8.4 Hz), 7.60 (d, 1H, *J* = 7.8 Hz), 7.51 (d, 2H, *J* = 8.4 Hz), 7.41 (d, 1H, *J* = 8.4 Hz), 7.20 (s, 1H), 7.17 (t, 1H, *J* = 7.5 Hz), 7.04 (t, 1H, *J* = 6.6 Hz), 4.18 (s, 2H), 3.74 (s, 3H), 3.15–3.09 (m, 4H); ^13^C NMR (150 MHz, DMSO-d_6_) *δ* 136.7, 133.6, 132.1 (2C), 131.2, 128.6 (2C), 127.6, 127.1, 121.3, 118.6, 118.4, 109.8, 108.7, 49.0, 46.9, 32.3, 21.5. HRMS *m*/*z* [M + H]^+^ calcd. 299.1310, found 299.1300.

##### 2-(1-Methyl-1*H*-indol-3-yl)-*N*-(4-methylbenzyl)ethan-1-amine hydrochloride (14)

2-(1-Methyl-1*H*-indol-3-yl)ethan-1-amine 5a (70 mg, 0.40 mmol) and 4-methylbenzaldehyde 2e (53 mg, 0.44 mmol) were dissolved in EtOH (3 ml mmol^−1^) and the mixture was stirred at rt for 12 h. NaBH_4_ (23 mg, 0.60 mmol) was then added, and the reaction was carried out according to the general procedure to afford compound 14 (23 mg, 23% yield). ^1^H NMR (600 MHz, DMSO-*d*_6_) *δ* 9.10 (bs, 2H), 7.56 (d, 1H, *J* = 7.8 Hz), 7.43–7.40 (m, 3H), 7.25 (d, 2H, *J* = 7.7 Hz), 7.20 (s, 1H), 7.18–7.15 (m, 1H), 7.06–7.03 (m, 1H), 4.13 (s, 2H), 3.74 (s, 3H), 3.11–3.08 (m, 4H), 2.32 (s, 3H); ^13^C NMR (150 MHz, DMSO-*d*_6_) *δ* 138.3, 136.7, 129.9 (2C), 129.2 (3C), 127.6, 127.0, 121.4, 118.6, 118.4, 109.8, 108.7, 49.7, 46.9, 32.3, 21.6, 20.8. HRMS *m*/*z* [M + H]^+^ calcd. 279.1856, found 279.1838.

##### *N*-(3-Chloro-4,5-dimethoxybenzyl)-2-(1*H*-indol-3-yl)ethan-1-amine hydrochloride (15)

2-(1*H*-Indol-3-yl)ethan-1-amine 1a (200 mg, 1.25 mmol) and 3-chloro-4,5-dimethoxybenzaldehyde 2f (250 mg, 1.25 mmol) were dissolved in MeOH (3 ml mmol^−1^) and the mixture was stirred at rt for 48 h. NaBH_4_ (81 mg, 2.13 mmol) was then added, and the reaction was carried out according to the general procedure to afford compound 15 (97 mg, 77% yield). ^1^H NMR (400 MHz, DMSO-*d*_6_) *δ* 11.0 (s, 1H), 9.64, (bs, 2H), 7.58 (d, 1H, *J* = 7.6 Hz), 7.47 (d, 1H, *J* = 1.4 Hz), 7.37 (d, 1H, *J* = 8.4 Hz), 7.29–7.24 (m, 2H), 7.10 (t, 1H, *J* = 7.6 Hz), 7.00 (t, 1H, *J* = 7.6 Hz), 4.14 (s, 2H), 3.87 (s, 3H), 3.76 (s, 3H), 3.22–3.05 (m, 4H); ^13^C NMR (100 MHz, DMSO-*d*_6_) *δ* 153.4, 144.9, 136.3, 129.0, 126.7 (2C), 123.3, 123.0, 121.2, 118.4, 118.2, 114.0, 111.6, 109.3, 60.3, 56.3, 49.1, 46.8, 21.6. HRMS *m*/*z* [M + H]^+^ calcd. 345.1365, found 345.1363.

##### *N*-(3-Chloro-4,5-dimethoxybenzyl)-2-(1-methyl-1*H*-indol-3-yl)ethan-1-amine hydrochloride (16)

2-(1-Methyl-1*H*-indol-3-yl)ethan-1-amine 5a (200 mg, 1.14 mmol) and 3-chloro-4,5-dimethoxybenzyl 2f (253 mg, 1.26 mmol) were dissolved in EtOH (3 ml mmol^−1^) and the mixture was stirred at rt for 12 h. NaBH_4_ (23 mg, 0.60 mmol) was then added, and the reaction was carried out according to the general procedure to afford compound 16 (140 mg, 34% yield). ^1^H NMR (600 MHz, DMSO-*d*_6_) *δ* 9.21 (bs, 2H), 7.57 (d, 1H, *J* = 7.8 Hz), 7.42 (d, 1H, *J* = 7.8 Hz), 7.37–7.33 (m, 1H), 7.25 (s, 1H), 7.23 (s, 1H), 7.16 (t, 1H, *J* = 7.8 Hz), 7.06 (t, 1H, *J* = 7.2 Hz), 4.14 (s, 2H), 3.86 (s, 3H), 3.76 (s, 3H), 3.75 (s, 3H), 3.15–3.13 (m, 2H), 3.11–3.10 (m, 2H); ^13^C NMR (150 MHz, DMSO-*d*_6_) *δ* 153.4, 144.9, 136.7, 129.0, 127.7, 127.0, 126.7, 123.0, 121.3, 118.6, 118.4, 114.0, 109.8, 108.7, 60.2, 56.3, 49.1, 46.8, 32.3, 21.4. HRMS *m*/*z* [M + H]^+^ calcd. 359.1521, found 359.1538.

##### 2-(1-Methyl-1*H*-indol-3-yl)-*N*-(naphthalen-2-ylmethyl)ethan-1-amine hydrochloride (17)

2-(1-Methyl-1*H*-indol-3-yl)ethan-1-amine 5a (100 mg, 0.57 mmol) and 2-naphthaldehyde 2g (117 mg, 0.74 mmol) were dissolved in EtOH (3 ml mmol^−1^) and allowed to reflux for 12 h. NaBH_4_ (33 mg, 0.86 mmol) was then added, and the reaction was carried out according to the general procedure to afford compound 17 (47 mg, 23% yield). ^1^H NMR (400 MHz, DMSO-*d*_6_) *δ* 9.18 (bs, 2H), 8.07 (s, 1H), 8.01 (d, 1H, *J* = 8.8 Hz), 7.98–7.92 (m, 2H), 7.70–7.66 (m, 1H), 7.61–7.55 (m, 3H), 7.42 (d, 1H, *J* = 8.4 Hz), 7.21 (s, 1H), 7.17 (t, 1H, *J* = 7.6 Hz), 7.06–7.00 (m, 1H), 4.38 (s, 2H), 3.74 (s, 3H), 3.22 (t, 2H, *J* = 7.6 Hz), 3.11 (t, 2H, *J* = 7.6 Hz); ^13^C NMR (150 MHz, DMSO-d_6_) *δ* 136.7, 132.8, 132.6, 129.7, 129.4, 128.4, 127.8, 127.7 (2C), 127.2, 127.0, 126.8, 126.7, 121.4, 118.6, 118.3, 109.8, 108.6, 50.1, 47.0, 32.3, 21.6. HRMS *m*/*z* [M+]^+^ calcd. 315.1259, found 315.1236.

#### General procedure for the synthesis of indoles 18–26

The corresponding substituted indole hydrochloride (1.0 eq.) and 2-methoxybenzaldehyde (1.3 eq.) were dissolved in MeOH (2 ml mmol^−1^), along with TEA (1.1 eq.). The reaction mixture was stirred at rt for 3 h, followed by the addition of NaBH_4_ (1.5 eq), and stirring was continued for an additional 1 h at rt. After completion, the reaction was quenched with a saturated solution of NaHCO_3_ and EtOAc was added. The water phase was extracted three times with EtOAc (3 × 150 ml), and the combined organic layers were washed once with brine, dried over Na_2_SO_4_, filtered, and concentrated. The yellow residue was dissolved in EtOAc (1 ml) and treated with 2 M HCl (1.0 eq.) in diethyl ether at 0 °C, then sonicated, filtered, and finally recrystallized from IPA to give the desired compounds.

##### 2-(5-Chloro-1*H*-indol-3-yl)-*N*-(2-methoxybenzyl)ethan-1-amine hydrochloride (18)

2-(5-Chloro-1*H*-indol-3-yl)ethan-1-amine hydrochloride 1c (120 mg, 0.61 mmol) and 2-methoxybenzaldehyde 2b (0. 11 g, 0.87 mmol) were dissolved in MeOH (2 ml mmol^−1^), along with TEA (104 μL, 0.74 mmol). The reaction was stirred at rt for 3 h, after which NaBH_4_ (35 mg, 0.92 mmol) was added, and the reaction was carried out according to the general procedure to afford compound 18 (130 mg, 60% yield). ^1^H NMR (400 MHz, DMSO-*d*_6_) *δ* 11.2 (s, 1H), 8.9 (s, 1H), 7.63 (s, 1H), 7.48–7.38 (m, 3H), 7.33 (s, 1H), 7.10 (d, 2H, *J* = 8.4 Hz), 7.02 (t, 1H, *J* = 7.4 Hz), 4.18 (s, 2H), 3.84 (s, 3H), 3.17–3.13 (m, 2H), 3.10–3.06 (m, 2H); ^13^C NMR (150 MHz, DMSO-*d*_6_) *δ* 157.5, 134.8, 131.4, 130.8, 127.9, 125.4, 123.3, 121.2, 120.4, 120.0, 117.5, 113.1, 111.1, 109.4, 55.6, 47.0, 45.0, 21.4. HRMS *m*/*z* [M + H]^+^ calcd. 315.1259, found 315.1236.

##### 2-(5-Chloro-1-methyl-1*H*-indol-3-yl)-*N*-(2-methoxybenzyl)ethan-1-amine hydrochloride (19)

2-(5-Chloro-1-methyl-1*H*-indol-3-yl)ethan-1-amine 5b (390 mg, 1.87 mmol), 2-methoxybenzaldehyde 2b (269 μl, 2.05 mmol) and TEA (286 μl, 2.05 mmol) were dissolved in EtOH (2 ml mmol^−1^) and allowed to stir at rt for 12 h. NaBH_4_ (177 mg, 2.80 mmol) was then added, and the reaction was carried out according to the general procedure to afford compound 19 (305 mg, 45% yield). ^1^H NMR (400 MHz, DMSO-*d*_6_) *δ* 8.84 (bs, 2H), 7.65 (d, 1H, *J* = 2.0 Hz), 7.47–7.41 (m, 3H), 7.31 (s, 1H), 7.17 (dd, 1H, *J*_1_ = 8.2, *J*_2_ = 2.2 Hz), 7.11 (d, 1H, *J* = 7.6 Hz), 7.04–7.00 (m, 1H), 4.18 (t, 2H, *J* = 6.0 Hz), 3.84 (s, 3H), 3.76 (s, 3H), 3.16–3.05 (m, 4H); ^13^C NMR (150 MHz, DMSO-*d*_6_) *δ* 157.5, 135.2, 131.4, 130.8, 129.6, 128.1, 123.5, 121.2, 120.4, 119.8, 117.7, 111.5, 111.1, 108.5, 55.6, 47.0, 44.9, 32.6, 21.2. HRMS *m*/*z* [M + H]^+^ calcd. 329.1416, found 329.1420.

##### *N*-(2-Methoxybenzyl)-2-(5-methyl-1*H*-indol-3-yl)ethan-1-amine hydrochloride (20)

2-(5-Methyl-1*H*-indol-3-yl)ethan-1-amine hydrochloride 1c (200 mg, 0.94 mmol) and 2-methoxybenzaldehyde 2b (168 mg, 1.23 mmol) were dissolved in MeOH (2 ml mmol^−1^) along with TEA (146 μL, 1.04 mmol). The reaction was allowed to stir at rt for 3 h, then NaBH_4_ (54 mg, 1.42 mmol) was added, and the reaction was carried out according to the general procedure to afford compound 20 (212 mg, 67% yield). ^1^H NMR (600 MHz, DMSO-*d*_6_) *δ* 10.8 (s, 1H), 8.95 (s, 2H), 7.48 (d, 1H, *J* = 7.8 Hz), 7.43 (t, 1H, *J* = 8.1 Hz), 7.29 (s, 1H), 7.25 (d, 1H, *J* = 8.5 Hz), 7.17 (s, 1H), 7.10 (d, 1H, *J* = 8.1 Hz), 7.02 (t, 1H, *J* = 7.5 Hz), 6.92 (d, 1H, *J* = 8.4 Hz), 4.18 (s, 2H), 3.83 (s, 3H), 3.16–3.06 (m, 4H), 2.38 (s, 3H); ^13^C NMR (150 MHz, DMSO-*d*_6_) *δ* 157.5, 134.7, 131.4, 130.8, 126.9 (2C), 123.4, 122.8, 120.4, 119.8, 117.6, 111.3, 111.1, 108.7, 55.6, 47.0, 45.0, 21.5, 21.3. HRMS *m*/*z* [M + H]^+^ calcd. 295.1805, found 295.1806.

##### 2-(6-Fluoro-1*H*-indol-3-yl)-*N*-(2-methoxybenzyl)ethan-1-amine hydrochloride (21)

2-(6-Fluoro-1*H*-indol-3-yl)ethan-1-amine hydrochloride 1d (200 mg, 0.93 mmol) and 2-methoxybenzaldehyde 2b (165 mg, 1.21 mmol) were dissolved in MeOH (2 ml mmol^−1^) along with TEA (143 μL, 1.02 mmol). The reaction was allowed to stir at rt for 3 h, then NaBH_4_ (54 mg, 1.42 mmol) was added, and the reaction was carried out according to the general procedure to afford compound 21 (160 mg, 51% yield). ^1^H NMR (400 MHz, DMSO-*d*_6_) *δ* 11.1 (s, 1H), 8.91 (s, 1H), 7.54 (dd, 1H, *J*_1_ = 8.8 Hz, *J*_2_ = 5.6 Hz), 7.48–7.41 (m, 2H), 7.24 (d, 1H, *J* = 2.0 Hz), 7.15 (dd, 1H, *J*_1_ = 10.0 Hz, *J*_2_ = 2.2 Hz), 7.10 (d, 1H, *J* = 8.4 Hz), 7.01 (t, 1H, *J* = 7.2 Hz), 6.91–6.86 (m, 1H), 4.17 (s, 2H), 3.83 (s, 3H), 3.18–3.07 (m, 4H); ^13^C NMR (100 MHz, DMSO-*d*_6_) *δ* 159.0 (d, *J*_1C–F_ = 236 Hz), 157.5, 136.1 (d, *J*_3C–F_ = 10 Hz), 131.4, 130.8, 124.0 (d, 1C, *J*_4C–F_ = 3.2 Hz), 123.6, 120.4, 119.8, 119.1 (d, *J*_3C–F_ = 10 Hz), 111.1, 109.6, 107.0 (d, 1C, *J*_2C–F_ = 25 Hz), 97.5 (d, *J*_2C–F_ = 25 Hz), 55.6, 47.0, 45.1 21.4. HRMS *m*/*z* [M + H]^+^ calcd. 299.1555, found 299.1551.

##### 2-(6-Fluoro-1-methyl-1*H*-indol-3-yl)-*N*-(2-methoxybenzyl)ethan-1-amine hydrochloride (22)

2-(6-Fluoro-1-methyl-1*H*-indol-3-yl)ethan-1-amine 5d (40 mg, 0.20 mmol), 2-methoxybenzaldehyde 2a (35 μl, 0.27 mmol) and TEA (29 μl, 0.28 mmol) were dissolved in EtOH (2 ml mmol^−1^) and allowed to stir at rt for 12 h. Then, NaBH_4_ (12 mg, 0.31 mmol) was added, and the reaction was carried out according to the general procedure to afford compound 22 (40 mg, 55% yield). ^1^H NMR (600 MHz, DMSO-*d*_6_) *δ* 8.80 (bs, 2H), 7.55–7.44 (m, 3H), 7.31–7.30 (m, 1H), 7.22 (s, 1H), 7.11–6.89 (m, 3H), 4.16 (s, 2H), 3.82 (s, 3H), 3.72 (s, 3H), 3.13–3.08 (m, 4H); ^13^C NMR (150 MHz, DMSO-*d*_6_) *δ* 159.2 (d, *J*_1C–F_ = 227 Hz), 157.5, 136.7 (d, *J*_3C–F_ = 10 Hz), 131.3, 130.7, 128.2 (d, *J*_4C–F_ = 3.5 Hz), 123.8, 120.4 (2C), 119.5 (d, *J*_3C–F_ = 10 Hz), 111.1, 109.2, 107.0 (d, *J*_2C–F_ = 24 Hz), 96.2 (d, *J*_2C–F_ = 26 Hz), 55.6, 47.1, 45.1, 32.5, 21.5. HRMS *m*/*z* [M + H]^+^ calcd. 313.1711, found 313.1726.

##### 2-(5-Methoxy-1*H*-indol-3-yl)-*N*-(2-methoxybenzyl)ethan-1-amine hydrochloride (23)

2-(5-Methoxy-1*H*-indol-3-yl)ethan-1-amine 1e (70 mg, 0.36 mmol) and 2-methoxybenzaldehyde 2a (55 mg, 0.40 mmol) were dissolved in EtOH (2 ml mmol^−1^) and allowed to reflux for 12 h. Then, the reaction mixture was cooled down to rt and NaBH_4_ (29 mg, 0.55 mmol) was added, and the reaction was carried out according to the general procedure to afford compound 23 (80 mg, 70% yield). ^1^H NMR (600 MHz, DMSO-*d*_6_) *δ* 10.8 (s, 1H), 8.86 (s, 2H), 7.47 (d, 1H, *J* = 7.2 Hz), 7.43 (t, 1H, *J* = 7.8 Hz), 7.26 (d, 1H, *J* = 8.4 Hz), 7.19 (d, 1H, *J* = 1.8 Hz), 7.10 (d, 1H, *J* = 8.4 Hz), 7.04–7.00 (m, 2H), 6.75 (dd, 1H, *J*_1_ = 8.7 Hz, *J*_2_ = 2.1 Hz), 4.18 (s, 2H), 3.82 (s, 3H), 3.77 (s, 3H), 3.15 (t, 2H, *J* = 7.8 Hz), 3.07 (t, 2H, *J* = 7.8 Hz); ^13^C NMR (150 MHz, DMSO-*d*_6_) *δ* 157.5, 153.2, 131.4, 131.4, 130.8, 127.1, 124.0, 120.4, 119.8, 112.2, 111.3, 111.1, 108.9, 100.1, 55.6, 55.5, 47.0, 45.0, 21.6. HRMS *m*/*z* [M + H]^+^ calcd. 311.1755, found 311.1724.

##### 2-(5-Methoxy-1-methyl-1*H*-indol-3-yl)-*N*-(2-methoxybenzyl)ethan-1-amine hydrochloride (24)

2-(5-Methoxy-1-methyl-1*H*-indol-3-yl)ethan-1-amine 5e (300 mg, 1.47 mmol), 2-methoxybenzaldehyde 2a (211 μl, 1.62 mmol) and TEA (225 μl, 1.62 mmol) were dissolved in EtOH (2 ml mmol^−1^) and allowed to stir at rt for 12 h. Then, NaBH_4_ (139 mg, 2.20 mmol) was added, and the reaction was carried out according to the general procedure to afford compound 24 (80 mg, 45% yield). ^1^H NMR (400 MHz, DMSO-*d*_6_) *δ* 8.93 (bs, 2H), 7.50–7.38 (m, 2H), 7.32 (d, 1H, *J* = 9.2 Hz), 7.16 (s, 1H), 7.10–7.00 (m, 3H), 6.82 (d, 1H, *J* = 8.4 Hz), 4.18 (s, 2H), 3.82 (s, 3H), 3.78 (s, 3H), 3.71 (s, 3H), 3.12–3.02 (m, 4H); ^13^C NMR (150 MHz, DMSO-d_6_) *δ* 157.5, 153.3, 132.0, 131.4, 130.8, 128.2, 127.4, 120.4, 119.7, 111.3, 111.1, 110.5, 108.1, 100.5, 55.6, 55.5, 46.9, 44.9, 32.5, 21.3. HRMS *m*/*z* [M + H]^+^ calcd. 325.1911, found 325.1912.

##### 3-(2-((2-Methoxybenzyl)amino)ethyl)-1*H*-indol-5-ol hydrochloride (25)

3-(2-Aminoethyl)-1*H*-indol-5-ol hydrochloride 1f (50 mg, 0.23 mmol) and 2-methoxybenzaldehyde 2a (48 mg, 0.35 mmol) were dissolved in MeOH (2 ml mmol^−1^), along with NaCNBH_3_ (22 mg, 0.35 mmol), and the reaction mixture was stirred at rt for 30 min. After completion, the solvent was removed and water was added (20 ml). 1 M HCl was added until pH 3 was reached and then chloroform was used to wash the aqueous phase. The water layer was treated with a 2 M NaOH solution up to pH 9 and then extracted with EtOAc, washed in turn with brine, dried over Na_2_SO_4_, filtered and concentrated. The crude was purified over column chromatography (eluent 90 : 9 : 1 DCM : MeOH : NH_4_OH). The resulting oil was dissolved in EtOAc (1 ml) and treated with 2 M HCl in diethyl ether at 0 °C, sonicated, filtered, and then recrystallized from IPA to give compound 25 (25 mg, 32% yield). ^1^H NMR (400 MHz, DMSO-*d*_6_) *δ* 10.7 (s, 1H), 8.98 (s, 2H), 8.68 (s, 1H), 7.50–7.40 (m, 2H), 7.16–7.09 (m, 3H), 7.02–7.00 (m, 1H), 6.84 (s, 1H), 6.63 (d, 1H, *J* = 8.0 Hz), 4.16 (s, 2H), 3.83 (s, 3H), 3.11–3.02 (m, 4H); ^13^C NMR (100 MHz, DMSO-d_6_) *δ* 157.5, 150.4, 131.4, 130.8 (2C), 127.4, 123.6, 120.4, 119.8, 111.8, 111.6, 111.1, 108.3, 102.0, 55.6, 47.0, 45.0, 21.6. HRMS *m*/*z* [M + H]^+^ calcd. 297.1598, found 297.1594.

##### 3-(2-((2-Methoxybenzyl)amino)ethyl)-1-methyl-1*H*-indol-5-ol hydrochloride (26)

3-(2-Aminoethyl)-1-methyl-1*H*-indol-5-ol 5f (100 mg, 0.36 mmol), 2-methoxybenzaldehyde 2a (63 μl, 0.47 mmol) and TEA (57 μl, 0.40 mmol) were dissolved in MeOH (2 ml mmol^−1^) and was stirred at rt for 12 h. NaBH_4_ (21 mg, 0.55 mmol) was then added, and the reaction was carried out according to the general procedure to afford compound 26 (40 mg, 31% yield). ^1^H NMR (400 MHz, DMSO-*d*_6_) *δ* 8.84 (bs, 1H), 8.75 (bs, 1H), 7.46–7.41 (m, 2H), 7.20 (d, 1H, *J* = 8.4 Hz), 7.12 (s, 1H), 7.09 (s, 1H), 7.02 (t, 1H, *J* = 7.4 Hz), 6.85 (d, 1H, *J* = 2.0 Hz), 6.69 (dd, 1H, *J*_1_ = 8.8 Hz, *J*_2_ = 1.6 Hz), 4.17 (s, 2H), 3.84 (s, 3H), 3.68 (s, 3H), 3.12 (t, 2H, *J* = 8.0 Hz), 3.00 (t, 2H, *J* = 7.6 Hz); ^13^C NMR (150 MHz, DMSO-*d*_6_) *δ* 157.4, 150.7, 131.5, 131.4, 130.9, 127.9, 127.7, 120.4, 119.8, 111.6, 111.1, 110.2, 107.3, 102.4, 55.6, 47.0, 45.0, 38.2, 32.4, 21.4. HRMS *m*/*z* [M + H]^+^ calcd. 311.1754, found 311.1748.

##### *N*-Benzyl-*N*-methyl-2-(1-methyl-1*H*-indol-3-yl)ethan-1-amine hydrochloride (29)

*N*-Methyl-2-(1-methyl-1*H*-indol-3-yl)ethan-1-amine 28 (50 mg, 0.26 mmol) and benzaldehyde 2a (35 μl, 0.34 mmol) were dissolved in EtOH (2 ml mmol^−1^), and allowed to reflux for 12 h. The reaction mixture was cooled down, and NaBH_4_ (15 mg, 0.39 mmol) was added, and the reaction was carried out according to the general procedure to afford compound 29 (40 mg, 48% yield). ^1^H NMR (400 MHz, DMSO-*d*_6_) *δ* 10.3 (bs, 1H), 7.60–7.57 (m, 2H), 7.53 (d, 1H, *J* = 7.6 Hz), 7.48–7.46 (m, 3H), 7.40 (d, 1H, *J* = 8.4 Hz), 7.21–7.14 (m, 2H), 7.03 (t, 1H, *J* = 7.6 Hz), 4.35 (s, 2H), 3.73 (s, 3H), 3.36–3.17 (m, 4H), 2.77 (s, 3H); ^13^C NMR (150 MHz, DMSO-d_6_) *δ* 136.7, 131.2 (2C), 130.4, 129.5, 128.9 (2C), 127.6, 127.0, 121.5, 118.7, 118.5, 109.8, 108.4, 58.4, 54.9, 48.8, 32.4, 19.7. HRMS *m*/*z* [M + H]^+^ calcd. 279.1856, found 279.1852.

##### *N*-Benzyl-*N*,*N*-dimethyl-2-(1-methyl-1*H*-indol-3-yl)ethan-1-aminium (30)

NaH 60% in mineral oil (34 mg, 0.83 mmol) was dissolved in dry DMF (2.5 ml mmol^−1^). Compound *N*-benzyl-2-(1*H*-indol-3-yl)ethan-1-amine 6 (70 mg, 0.29 mmol) was also dissolved in dry DMF (0.5 ml mmol^−1^), and at 0 °C was added slowly into the solution of NaH. After completion of the addition, the mixture was stirred at rt for 30 minutes. The reaction was then cooled down to 0 °C, and CH_3_I (35 μl, 0.83 mmol) was added dropwise, and stirred at rt for 4 hours. After completion, the residue was dissolved in H_2_O (20 ml) and extracted with 3 × 20 ml EtOAc, and the combined organic layers were washed with brine dried over Na_2_SO_4_, filtered, and concentrated. The obtained yellow oil was dissolved in EtOAc (1 ml), and treated with 2 M HCl in diethyl ether at 0 °C, sonicated, filtered, and finally recrystallized from IPA to give compound 30 (40 mg, 34% yield). ^1^H NMR (400 MHz, DMSO-*d*_6_) *δ* 7.65 (d, 1H, *J* = 8.4 Hz), 7.60–7.53 (m, 4H), 7.44 (d, 1H, *J* = 8.4 Hz), 7.26 (s, 1H), 7.19 (t, 1H, *J* = 7.6 Hz), 7.08 (t, 1H, *J* = 7.6 Hz), 4.66 (s, 2H), 3.77 (s, 3H), 3.52–3.50 (m, 2H), 3.28 (m, 2H), 3.10 (s, 6H); ^13^C NMR (100 MHz, CDCl_3_) *δ* 136.7, 133.0 (2C), 130.3, 129.0 (2C), 128.1, 127.8, 126.9, 121.5, 118.7, 118.5, 109.9, 107.6, 66.4, 63.6, 49.1, 40.1, 32.4, 18.3. HRMS *m*/*z* [M + H]^+^ calcd. 293.2013, found 293.2017.

#### General procedure for the synthesis of indoles 31–33

The substituted tryptamine hydrochlorides 1c and 1d (1.0 eq.) and the corresponding benzaldehydes 2a and 2b (1.3 eq.) were dissolved in EtOH (2 ml mmol^−1^), and allowed to reflux for 12 h. After completion, the reaction was quenched with a saturated solution of NaHCO_3_, and EtOAc (50 ml) was added. The water phase was extracted with 3 × 50 ml EtOAc, and the combined organic layers were washed once with brine and dried over Na_2_SO_4_, filtered, and concentrated. The light brown residue was dissolved in EtOAc (1 ml), and treated with 2 M HCl (1.0 eq.) in diethyl ether at 0 °C, then sonicated, filtered, and finally recrystallized from IPA to give the desired products as racemic mixtures.

##### 1-(2-Methoxyphenyl)-6,9-dimethyl-2,3,4,9-tetrahydro-1*H*-pyrido[3,4-*b*]indole (31)

2-(1,5-Dimethyl-1*H*-indol-3-yl)ethan-1-amine hydrochloride 1c (100 mg, 0.52 mmol) and 2-methoxybenzaldehyde 2b (90 μl, 0.69 mmol) were dissolved in EtOH (2 ml mmol^−1^), and allowed to reflux for 12 h. The reaction was then carried out according to the general procedure to afford compound 31 as a racemate (110 mg, 60% yield). ^1^H NMR (400 MHz, DMSO-d_6_) *δ* 10.0 (bs, 1H), 8.93 (bs, 1H), 7.50 (t, 1H, *J* = 7.8 Hz), 7.38 (s, 1H), 7.34 (d, 1H, *J* = 8.4 Hz), 7.25 (d, 1H, *J* = 8.4 Hz), 7.06 (d, 1H, *J* = 8.4 Hz), 6.94 (t, 1H, *J* = 7.4 Hz), 6.66 (d, 1H, *J* = 6.4 Hz), 6.15 (s, 1H), 3.97 (s, 3H), 3.43–3.40 (m, 1H), 3.24 (s, 3H), 3.08–3.00 (m, 3H), 2.42 (s, 3H); ^13^C NMR (100 MHz, DMSO-*d*_6_) *δ* 156.9, 135.7, 131.7, 130.3, 128.3, 128.0, 125.4, 123.7, 121.2, 120.6, 118.0, 111.6, 109.4, 107.2, 56.0, 47.8, 37.8, 29.5, 21.1, 18.1. HRMS *m*/*z* [M + H]^+^ calcd. 307.1766, found 307.1769.

##### 7-Fluoro-1-phenyl-2,3,4,9-tetrahydro-1*H*-pyrido[3,4-*b*]indole hydrochloride (32)

2-(6-Fluoro-1*H*-indol-3-yl)ethan-1-amine hydrochloride 1d (100 mg, 0.46 mmol) and benzaldehyde 2a (64.2 mg, 0.60 mmol) were dissolved in EtOH (2 ml mmol^−1^), and allowed to reflux at 90 °C for 12 h. The reaction was then carried out according to the general procedure to afford compound 32 as a racemate (75 mg, 53% yield). ^1^H NMR (400 MHz, DMSO-*d*_6_) *δ* 11.0 (s, 1H), 10.2 (bs, 1H), 9.43 (bs, 1H), 7.56–7.42 (m, 6H), 7.10–6.90 (m, 2H), 5.93 (s, 1H), 3.41–3.35 (m, 2H), 3.15–3.00 (m, 2H); ^13^C NMR (100 MHz, DMSO-*d*_6_) *δ* 159.3 (d, *J*_1C–F_ = 240 Hz), 136.5 (d, *J*_3C–F_ = 10 Hz), 134.5, 130.0 (2C), 129.8 (2C), 129.0 (d, *J*_4C–F_ = 3.5 Hz), 119.3 (d, *J*_3C–F_ = 10 Hz), 107.6 (d, *J*_2C–F_ = 25 Hz), 107.4, 97.6 (d, *J*_2C–F_ = 25 Hz), 79.2, 55.4, 18.0. HRMS *m*/*z* [M + H]^+^ calcd. 267.1293, found 267.1019.

##### 7-Fluoro-1-(2-methoxyphenyl)-2,3,4,9-tetrahydro-1*H*-pyrido[3,4-b]indole (33)

2-(6-Fluoro-1*H*-indol-3-yl)ethan-1-amine hydrochloride 1d (100 mg, 0.46 mmol) and 2-methoxybenzaldehyde 2b (115 mg, 0.84 mmol) were dissolved in EtOH (2 ml mmol^−1^), and allowed to reflux for 12 h. The reaction was then carried out according to the general procedure to afford compound 33 as a racemate (85 mg, 55% yield). ^1^H NMR (400 MHz, DMSO-*d*_6_) *δ* 11.0 (s, 1H), 10.0 (bs, 1H), 9.0 (bs, 1H), 7.56–7.45 (m, 2H), 7.21 (d, 1H, *J* = 8.3 Hz), 7.08 (dd, 1H, *J*_1_ = 10.0 Hz, *J*_2_ = 2.4 Hz), 6.97 (t, 1H, *J* = 7.5 Hz), 6.94–6.88 (m, 2H), 6.03 (s, 1H), 3.92 (s, 3H), 3.49–3.38 (m, 1H), 3.28–3.17 (m, 1H), 3.12–2.95 (m, 2H); ^13^C NMR (100 MHz, DMSO-*d*_6_) *δ* 159.4 (d, *J*_1C–F_ = 242 Hz), 157.2 (2C), 136.4 (d, *J*_3C–F_ = 10 Hz), 131.4, 130.4, 128.6, 122.5, 120.5, 119,3 (d, *J*_3C–F_ = 10 Hz), 111.5 (2C), 107.9, 107.4 (d, *J*_2C–F_ = 25 Hz), 97.6 (d, *J*_2C–F_ = 25 Hz), 56.0 (2C), 49.2, 18.2. HRMS *m*/*z* [M + H]^+^ calcd. 297.1398, found 297.1388.

### *In silico* predictions and cell viability assay

*In silico* predictions of toxicity endpoints for selected indole-based inhibitors were performed using ProTox 3.0 (https://tox.charite.de/protox3/),^35^ which estimates various toxicity classes based on the molecular structure. SwissADME (https://www.swissadme.ch/)^36^ was used to evaluate physicochemical properties and lead-likeness.

Cell viability was assessed using the resazurin reduction assay in *Spodoptera frugiperda* (Sf9) and human embryonic kidney (HEK293) cells. Both cell types were seeded at 20 000 cells per well in 96-well plates and allowed to attach and reach approximately 50% confluence before compound addition. Cells were treated with three concentrations of inhibitor 15 for 24 h. Cells that received the same DMSO concentration as experimental wells but no inhibitor served as vehicle controls (100% viability reference). Staurosporine (STS) was included as a positive cytotoxic control, and propranolol (Prop) as a non-cytotoxic reference treatment. Sf9 cells were maintained at 27 °C, and HEK293 cells at 37 °C, in their respective culture media. After compound exposure, resazurin solution was added directly to each well to a final concentration of 40 μM (10 μg mL^−1^), and plates were incubated for 3 h under standard culture conditions. Fluorescence was measured at an excitation wavelength of 535 nm and an emission wavelength of 595 nm using a microplate reader. Cell viability was expressed as a percentage relative to DMSO vehicle controls. All substances were analyzed in triplicate on two separate occasions (three wells per concentration per experiment).

### Protein expression

The expression of the recombinant AChE1 enzyme from mosquitoes (*An. gambiae, and Ae. aegypti*), and the recombinant vertebrate enzyme *h*AChE was performed as described in a previous publication.^37^

### IC_50_ determination

The *in vitro* biochemical evaluation has been performed using the activity-based Ellman assay.^38^ The IC_50_ values for the synthesized indole derivatives were determined on the recombinant *Ag*AChE1, *Aa*AChE1 and *h*AChE according to the following procedure. Freshly prepared stock solutions of the indole compounds were prepared from solid material dissolved in DMSO at a concentration of 100 mM. The dilution series were prepared in 0.1 M sodium phosphate buffer (pH 7.4). Eight different concentrations of indole compounds were used with a maximum of 1 mM. The activity measurements were performed using the non-purified recombinant enzyme in growth medium, and the enzymatic activity was measured using the Ellman assay, adjusted to a 96-well format. The compounds were incubated along with the enzyme for 5 min at rt, then the reaction was initiated with the addition of acetylcholine iodide (ATChI) and the substrate and the enzymatic reaction was measured by monitoring changes in the absorbance of individual wells at 412 nm over 65 s in a synergy H4 plate reader (Molecular Devices). The assay was performed at 30 °C in a final assay volume of 200 μl of 0.1 M phosphate buffer (pH 7.4) containing 0.2 mM of the reagent 5,5′-dithiobis (2-nitrobenzoic acid) and 1 mM of the substrate acetylthiocholine iodide. The average slope determined for eight positive controls (where the inhibitor was replaced with phosphate buffer) on each plate was taken to represent 100% activity, and the activity observed in the sample wells was quantified in relation to this value. IC_50_ values were calculated using nonlinear regression (curve-fitting) in GraphPad prism and the log [inhibitor] *vs.* response variable slop equation was fitted using four parameters.

### Generation, collection, and refinement of crystal structures

The catalytic domain of AChE from *m*AChE was expressed in HEK293F cells, purified, and crystallized following previously established protocols.^39^ Briefly, HEK293F cells were cultured in suspension using Freestyle 293 and Glutamax media (Gibco), supplemented with 20 μg ml^−1^ Gentamicin (Gibco). The *m*AChE-containing culture supernatant was harvested by centrifugation, and the enzyme was purified from the clarified supernatant through a series of affinity and size-exclusion chromatography steps. Protein crystallization was performed using the hanging drop vapor diffusion method. The protein solution, at a concentration of 10 mg ml^−1^, was mixed with a reservoir solution composed of 27–30% (w/v) PEG750MME and 0.1 M HEPES buffer, pH 6.9–7.1. To form binary (inhibitor·AChE) complexes, inhibitors were soaked into the pre-formed *m*AChE crystals prior to flash freezing in liquid nitrogen, as described in a previous study.^40^ X-ray diffraction data were collected at the MAXIV synchrotron (Lund, Sweden) using the Biomax beamline equipped with an Eiger detector. The collected data were indexed and integrated using XDS^41^ and scaled with AIMLESS.^42^ Initial phases were determined by rigid-body refinement using a modified apo structure of *m*AChE (PDB: 1J06) as a starting model. Further crystallographic refinement and manual model building were conducted using the Phenix software^43^ suite and COOT.^44^ Model validation was performed with MolProbity (integrated within Phenix) and the wwPDB Validation Service (https://validate-rcsb-1.wwpdb.org/).

### Molecular dynamics (MD) simulations

#### System preparation for MD simulations

MD simulations of *m*AChE·9 were based on coordinates from the X-ray structure (PDB: 9SNJ). Coordinates for *Ag*AChE1·9 were obtained by superposing the X-ray structure of apo *Ag*AChE1 (PDB: 5YDI) against *m*AChE·9. The binding pose of inhibitor 9 was thereafter merged with *Ag*AChE1, followed by altering the conformation of Tyr489_Ag_ (corresponding to Tyr337_m_) to avoid clashes. Inhibitor 9 was geometry optimized followed by calculation of electrostatic surface potentials (ESPs) using the HF/6-31G* basis set with Gaussian 09. The secondary amine in the linker of 9 was protonated, *i.e.* positively charged. Partial atomic charges were calculated using the restrained electrostatic potential (RESP) method using the antechamber program of AmberTools. Other parameters were assigned by the general amber force field (GAFF). Files were converted to GROMACS format using the acpype python script. Pdb2gmx within GROMACS was used for the generation of topology and coordinate files, with the AMBER99SB-ILDN force field.^45^

#### MD simulations

Each system was solvated using a dodecahedral periodic box of TIP3P water. Sodium ions were added to neutralize the system that were then energy minimized using the steepest decent algorithm. Heating to 300 K was thereafter performed over a 100 ps NVT simulation. This was followed by a 500 ps NPT simulation to equilibrate the pressure to 1 atm. During both of these simulations, the heavy atoms were restrained at their starting positions with a force constant of 1000 kJ mol^−1^ nm^2^. These restraints were stepwise removed over a 1 ns NPT simulation. The Berendsen thermostat was used for regulating the temperature and pressure. A time step of 2 fs was used for all simulations, constraining bonds using the parallel LINCS algorithm. Short range non-bonded interactions were computed for atom pairs within a cutoff of 14 Å. Long-range electrostatic interactions were calculated using the particle-mesh-Ewald summation method, using fourth-order cubic interpolation with a 1.2 Å grid spacing. Five replicates of 100 ns MD simulations were performed for *m*AChE·9 and *Ag*AChE1·9, respectively, with varying initial velocities. All simulations were run with GROMACS 5.1.4.^45^

#### Analysis

Root-mean-square deviation (RMSD) values were calculated using the gmx rms module in GROMACS,^45^ superposing against the main chain atoms of the NPT equilibrated structure. According to the resulting RMSD values the simulation converged after 50 ns. Thus, all subsequent analyses were performed using the concatenated 50–100 ns of each simulation. Root-mean-square fluctuation (RMSF) values were calculated using the gmx rmsf module. Pairwise minimum distances between selected atoms were calculated using the gmx pairdist module. Principal component analysis (PCA) was performed by calculating the mass-weighted covariance matrix of heavy atoms of inhibitor 9 using the gmx covar module, after superposing the trajectory against the main chain heavy atoms of the NPT equilibrated structure. Eigenvectors and eigenvalues were generated, and projections of the trajectory to eigenvector 1–3 were calculated using the gmx anaeig module. Cluster analysis of the generated binding poses of 9 was performed using gmx_clusterByFeatures^46^ including eigenvector 1–3, using the *K*-means algorithm and the Elbow method with a threshold of 3.0% on the sum of square residual to sum of square total ratio. The water occupancy was calculated using the gmx trjorder module, using a cutoff of 3.5 Å as the specified hydrogen bonding distance for selected heavy atoms.

### *In vivo* experiments

*Ae. aegypti* Mombasa strain and *An. gambiae* Kisumu strain from Kenya were used to test the insecticidal activities of the compounds. These mosquitoes have been colonized at KEMRI for over 20 years and are routinely tested to verify their susceptibility to permethrin and deltamethrin in accordance with WHO tube bioassay guidelines using diagnostic concentrations of 0.75% permethrin and 0.05% deltamethrin impregnated on filter paper. Mosquito rearing was carried out in an insectary maintained at 27–28 °C *ca.* 80% humidity, on a 12/12 h light/darkness cycle, and maintained at optimal larval concentrations to avoid possible effects of competition. For mosquito tests, nonblood fed, five-day old female mosquitoes were used, and testing was performed in batches of approximately five mosquitoes (100 in total per compound). Five mosquitoes were placed in a 500 ml paper cup and anesthetized by placing the cup in a −20 °C freezer for 3 min. Thereafter, for the topical application tests, the mosquitoes were gently poured onto a plate refrigerated at −20 °C overlaid with a paper towel, and the compound solution (acetone, 0.1 μl) was deposited on the upper part of the pronotum using a micro-pipette. As a negative control, 0.1 μl of pure acetone was applied on some mosquitoes, and as a positive control propoxur insecticide was used (Tables S9 and S10). After the topical application, the mosquitoes were returned to the paper cups and placed back in the insectary, where they were given a glucose meal and maintained under standard conditions. Mosquito mortality was recorded after 24 h.

## Conflicts of interest

There is no conflict of interest to declare.

## Supplementary Material

MD-017-D5MD00797F-s001

## Data Availability

The data supporting this article have been included as part of the supplementary information (SI). Crystallographic data for the two new protein–ligand complexes *m*AChE·8 and *m*AChE·9 have been deposited at the RCSB Protein Data Bank (PDB) under PDB entry codes 9SND and 9SNJ prior to publication and can be obtained from https://www.rcsb.org/. Supplementary information: chemical structures of starting materials; supplementary schemes of synthesis; dose–response IC_50_ curves for inhibition kinetics; table of data collection and refinement statistics of X-ray crystallography structures; MD simulations data; *in vivo* raw data tables; NMR spectra. See DOI: https://doi.org/10.1039/d5md00797f.
