# Supplementary material for: Potent and selective indole-based inhibitors targeting disease-transmitting mosquitoes
Source: RSC Med Chem. 2025 Nov 19;17(2):1166–86. doi: 10.1039/d5md00797f (PMC12805588; doi:10.1039/d5md00797f)

Supplementary Information (SI) for RSC Medicinal Chemistry.
This journal is © The Royal Society of Chemistry 2025

Potent and Selective Indole-based Inhibitors Targeting Disease-Transmitting Mosquitoes

R. Rajeshwari[1], V. Duvauchelle[1], C. Lindgren[1], K. Stangner[1], S. Knutsson[1], N. Forsgren[2], F. Ekström[2], L. Kamau[3], A. Linusson*[1]

[1]*Department of Chemistry, Umeå University (Sweden);* [2]*Swedish Defense Research Agency, Umeå (Sweden);* [3]*Centre of Biotechnology Research and Development, Kenya Medical Research Institute, Nairobi, Kenya*
*Corresponding author. E-mail address: anna.linusson@umu.se

Table of Contents

IN SILICO PREDICTIONS AND CELL VIABILITY ASSAY 2
STARTING MATERIAL STRUCTURES 4
SUPPLEMENTARY SYNTHETIC SCHEMES 5
DOSE-RESPONSE-CURVES FOR INHIBITION KINETICS 6
X-RAY CRYSTALLOGRAPHY 10
MD SIMULATIONS DATA 11
IN VIVO RAW DATA 16
NMR SPECTRA OF INTERMEDIATES 18
NMR SPECTRA OF FINAL COMPOUNDS 36

In silico predictions and cell viability assay

Table S1. In silico predictions of selected indole-based inhibitors against toxicity endpoints[a]

Compound	Toxicity end points		
	Cytotoxicity	Mutagenicity	Carcinogenicity
**8**	Inactive (0.61)	Inactive (0.59)	Inactive (0.64)
**9**	Active (0.50)	Active (0.57)	Inactive (0.61)
**10**	Inactive (0.70)	Active (0.95)	Active (0.66)
**15**	Inactive (0.53)	Inactive (0.63)	Inactive (0.63)
**16**	Active (0.52)	Inactive (0.54)	Inactive (0.61)
**18**	Inactive (0.58)	Inactive (0.61)	Inactive (0.67)

[a] The toxicity end points were predicted with ProTox 3.0 (https://tox.charite.de/protox3/)

Table S2. In silico assessment of selected indole-based inhibitors for physicochemical properties and lead-likeness[a]

ID	MW	TPSA	iLOGP	XLOGP3	ESOL Solubility (mg/ml)	ESOL Class	PAINS #alerts	Lead likeness #violations	Lipinski #violations
**8**	280.36	37.05	2.81	3.34	4.29e-02	Soluble	0	0	0
**9**	294.39	26.19	3.55	3.30	4.13e-02	Soluble	0	0	0
**10**	296.34	78.22	2.38	2.54	1.22e-01	Soluble	0	0	0
**15**	344.84	46.28	3.27	3.94	1.19e-02	Moderately soluble	0	1	0
**16**	358.86	35.42	3.91	3.90	1.12e-02	Moderately soluble	0	2	0
**18**	314.81	37.05	2.89	3.97	1.25e-02	Moderately soluble	0	1	0

[a] The in silico assessment was made with SwissADME (https://www.swissadme.ch/)

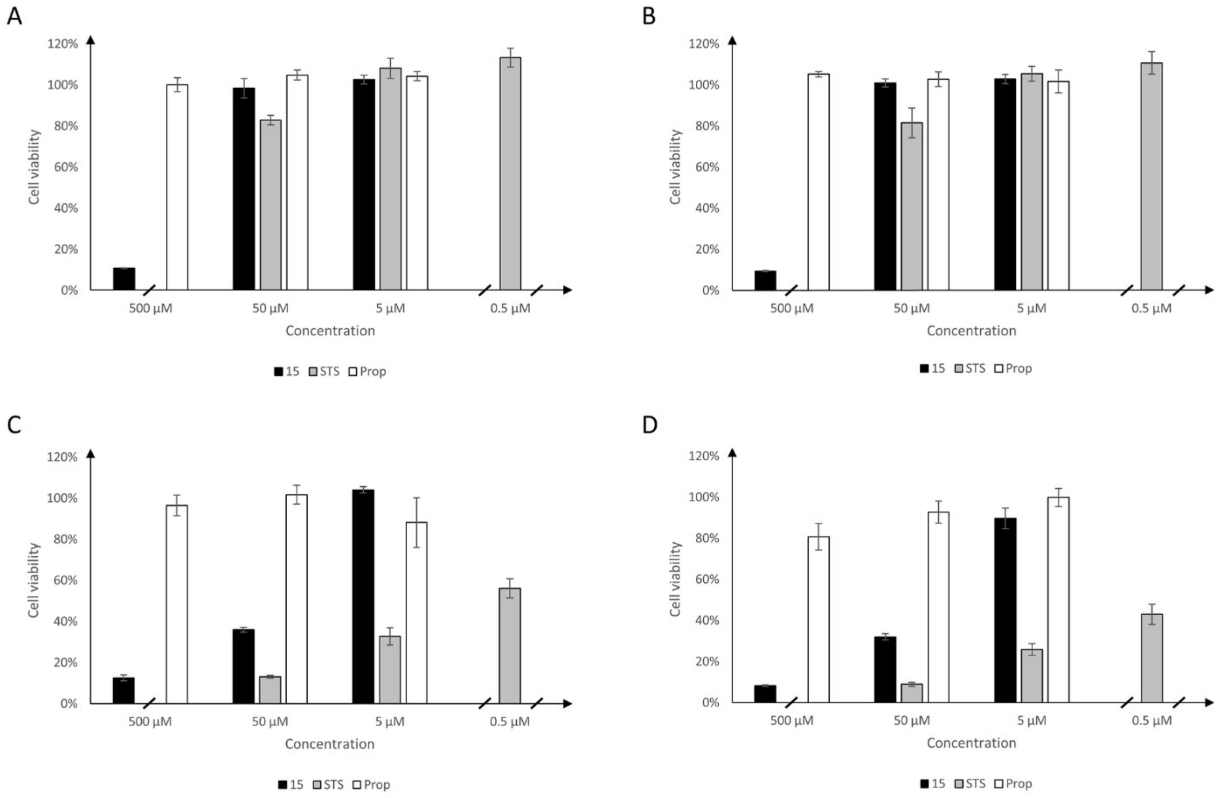

Figure S1. Assessment of cell viability after exposure to different concentrations of inhibitor 15 using the resazurin assay. (A-B) *Spodoptera frugiperda* (Sf9) insect cells. (C-D) Human embryonic kidney (HEK293) cells. Cells were exposed to three concentrations of inhibitor **15** for 24 h on two separate occasions, and viability was assessed using the resazurin reduction assay. Staurosporine (STS) and propranolol (Prop) were included as reference cytotoxic controls. Cell viability is expressed as a percentage relative to untreated controls. Data represent the mean ± SD of three independent experiments (n = 3).

Starting material structures

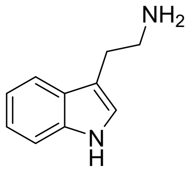

1a

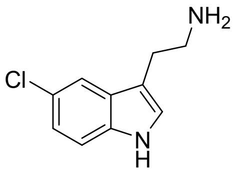

1b

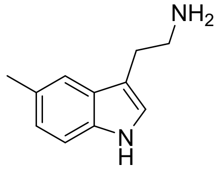

1c

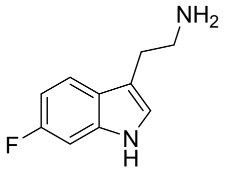

1d

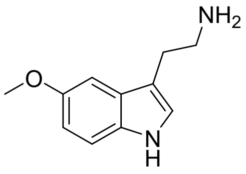

1e

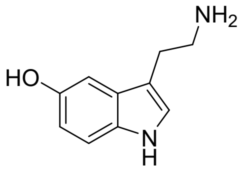

1f

Figure S2. Indoles (**1a-f**) as starting materials.

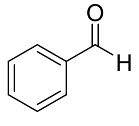

2a

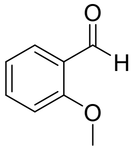

2b

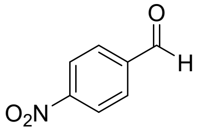

2c

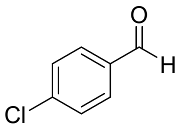

2d

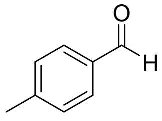

2e

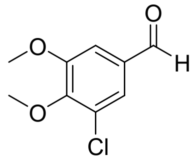

2f

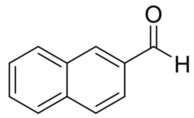

2g

Figure S3. Aldehydes (**2a-g**) as starting materials.

Supplementary synthetic schemes

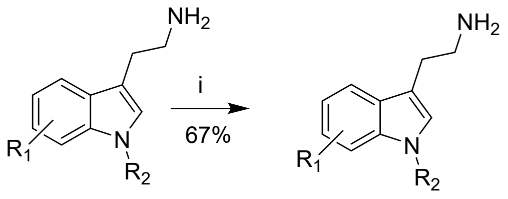

5e **5f**

R_1 = OCH_3
R_2 = CH_3

R_1 = OH
R_2 = CH_3

Scheme S1. Reagent and conditions: i) BBr_3, DCM -70˚C-rt, 12h.

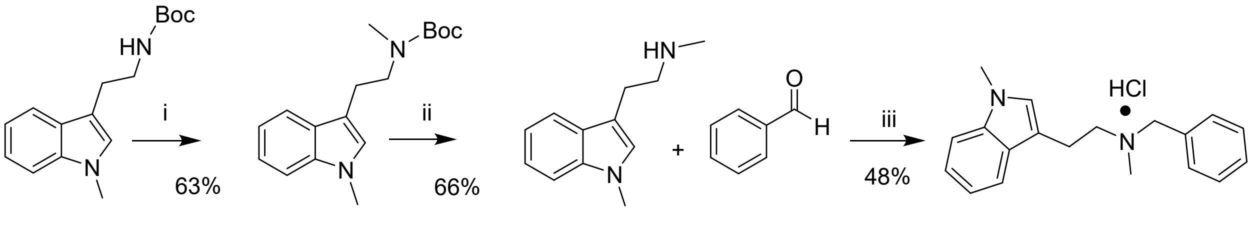

4a **27** **28** **2a** **29**

Scheme S2. Reagent and conditions: i) NaH, DMF 0˚C- 90˚C; CH_3I, 0˚C-rt, 12h. ii) TFA, DCM 0˚C-rt, 5h. iii) EtOH or MeOH, reflux, 12h; $NaBH_4$, 0˚C-rt; 12h 2M HCl in ether.

Dose-response-curves for inhibition kinetics

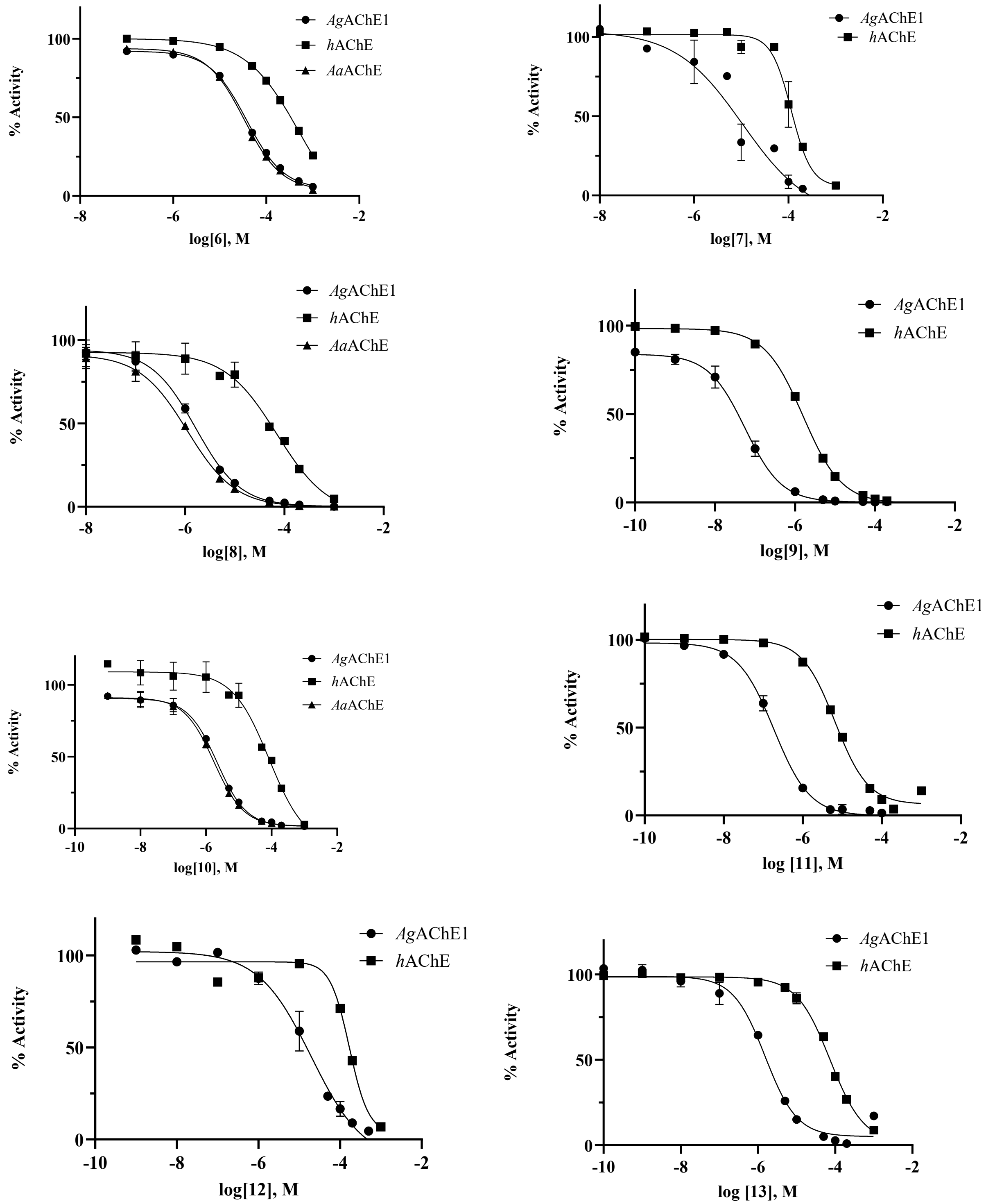

Figure S4. Dose response curves showing the inhibition of *Ag*AChE1 (dots), *Aa*AChE1 (triangle), and *h*AChE (squares) at different concentrations of inhibitors.

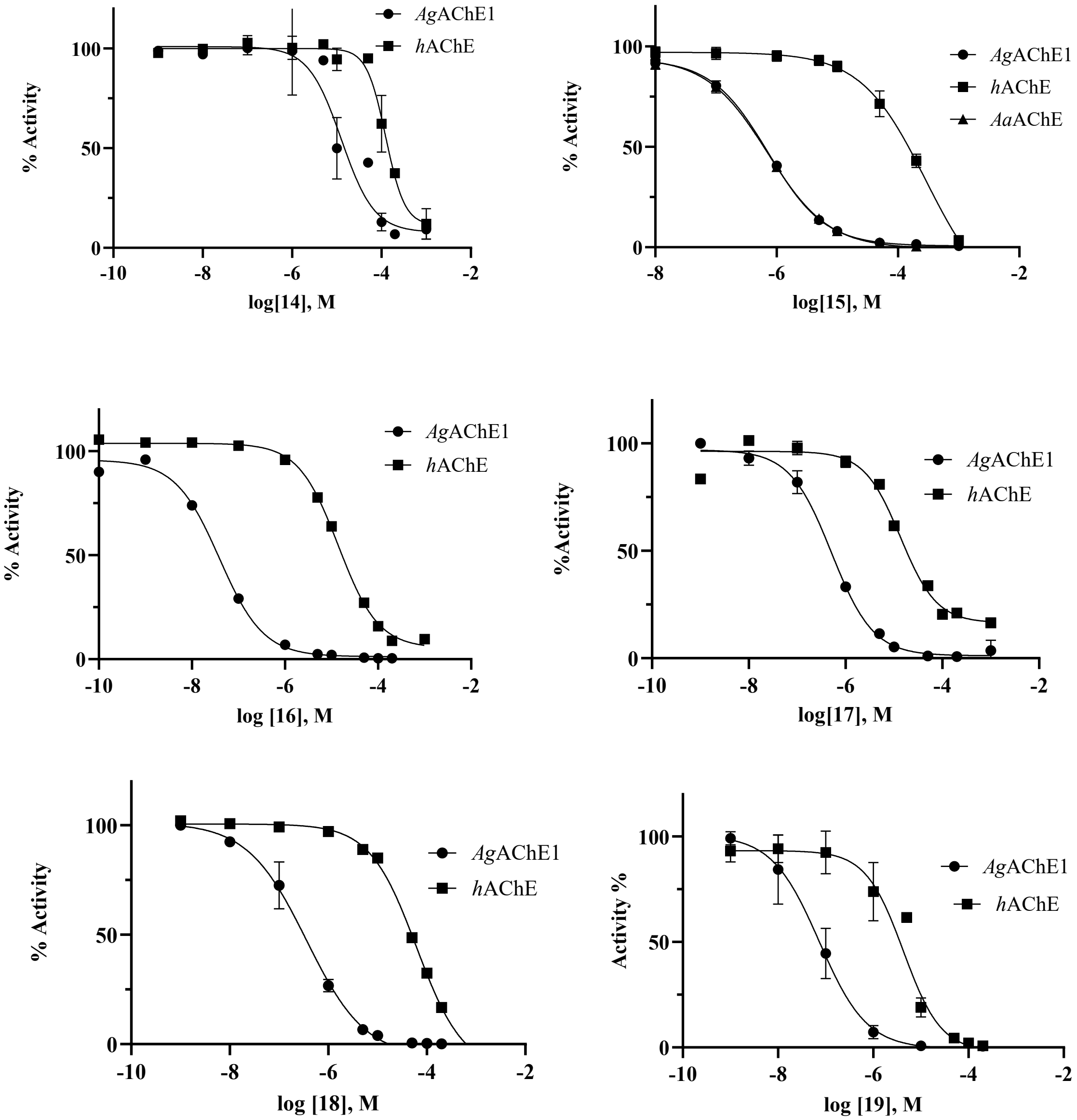

Figure S4 continued. Dose response curves showing the inhibition of *Ag*AChE1 (dots), *Aa*AChE1 (triangle), and *h*AChE (squares) at different concentrations of inhibitors.

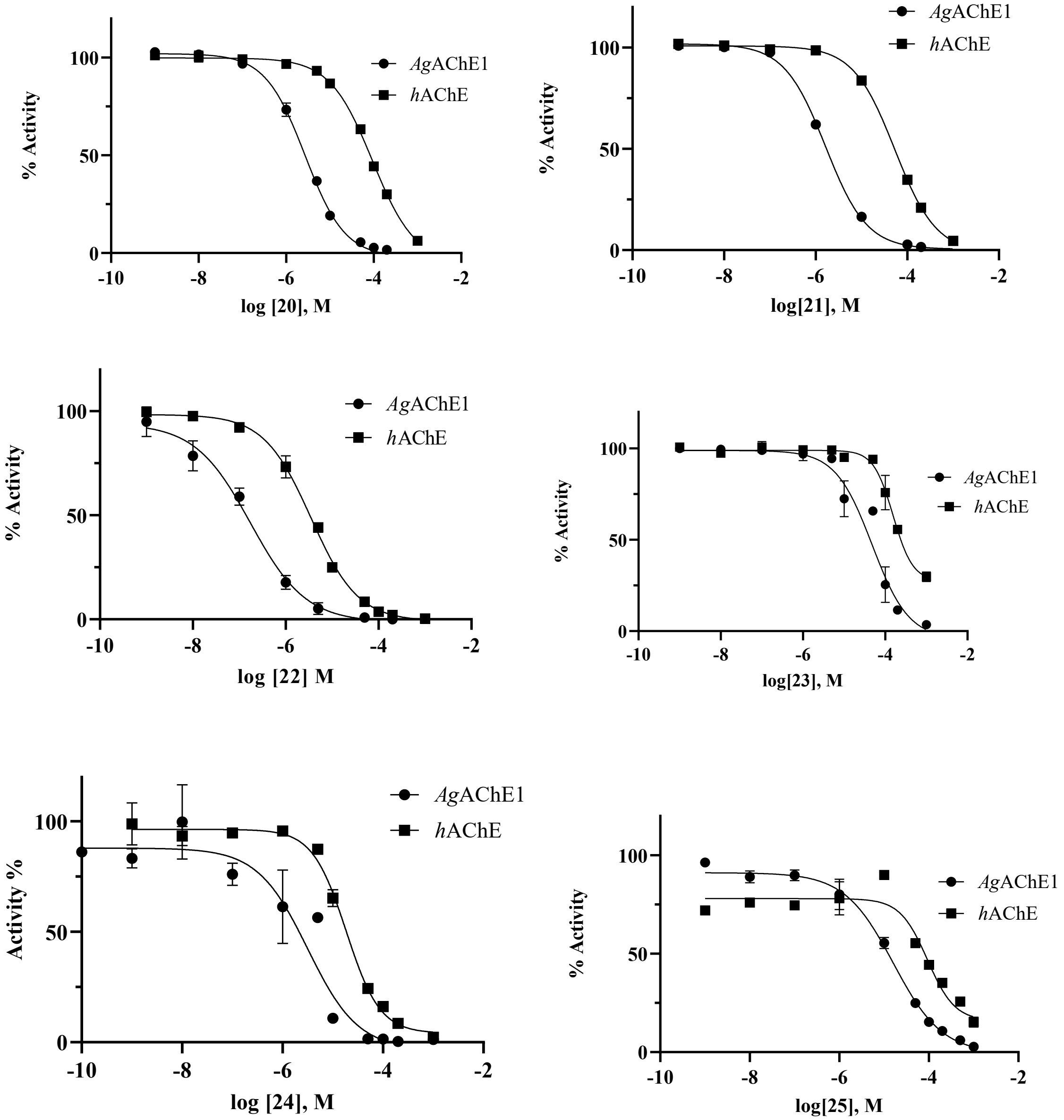

Figure S4 continued. Dose response curves showing the inhibition of *Ag*AChE1 (dots), *Aa*AChE1 (triangle), and *h*AChE (squares) at different concentrations of inhibitors.

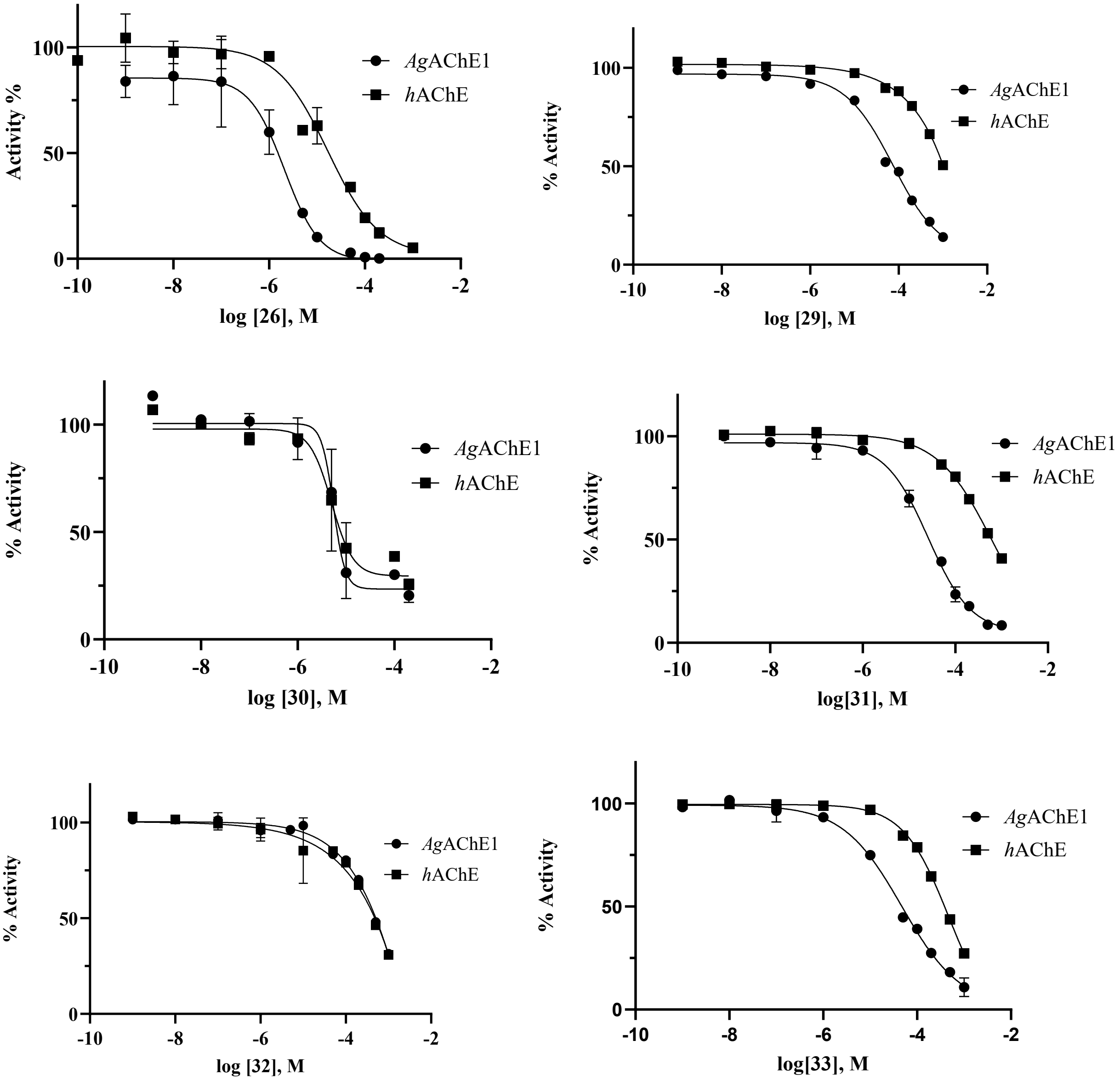

Figure S4 continued. Dose response curves showing the inhibition of *Ag*AChE1 (dots), *Aa*AChE1 (triangle), and *h*AChE (squares) at different concentrations of inhibitors.

X-ray crystallography

Table S3. Data collection and refinement statistics

	*m*AChE•8	*m*AChE•9
**PDB entry code**	9SND	9SNJ
**Resolution range (Å)**	29.68 - 2.4 (2.48 - 2.4)	29.27 - 2.3 (2.36 - 2.3)
**Space group**	P 21 21 21	P 21 21 21
**Unit cell**	78.353 111.104 227.383 90 90 90	79.493 112.581 227.811 90 90 90
**Total reflections**	596475 (40287)	678925 (49391)
**Unique reflections**	168071 (11817)	190385 (14316)
**Multiplicity**	3.5 (3.4)	3.6 (3.5)
**Completeness (%)**	99.34 (99.08)	99.68 (99.29)
**Mean I/sigma (I)**	7.78 (0.96)	8.40 (1.49)
**Wilson B-factor**	48.62	47.39
**R-merge**	0.08845 (0.8472)	0.07242 (0.6556)
**R-meas**	0.1043 (1.002)	0.08531 (0.7763)
**R-pim**	0.05486 (0.5298)	0.04464 (0.4103)
**CC1/2**	0.996 (0.823)	0.996 (0.894)
**CC***	0.999 (0.95)	0.999 (0.972)
**Reflections used in refinement**	77905 (6982)	91241 (6880)
**Reflections used for R-free**	1533 (115)	1798 (142)
**R-work**	0.2088 (0.3090)	0.1795 (0.2382)
**R-free**	0.2232 (0.2973)	0.2033 (0.2826)
**Number of non-hydrogen atoms**	8772	8866
**macromolecules**	8356	8373
**Ligands**	163	135
**Solvent**	253	358
**Protein residues**	1068	1068
**RMS (bonds)**	0.002	0.002
**RMS (angles)**	0.57	0.60
**Ramachandran favored (%)**	95.85	96.42
**Ramachandran allowed (%)**	3.49	2.92
**Ramachandran outliers (%)**	0.66	0.66
**Rotamer outliers (%)**	0.23	0.34
**Clashscore**	2.20	1.55
**Average B-factor**	64.44	64.96
**Macromolecules**	64.41	65.06
**Ligands**	79.14	70.23
**Solvent**	56.45	60.64

MD simulations data

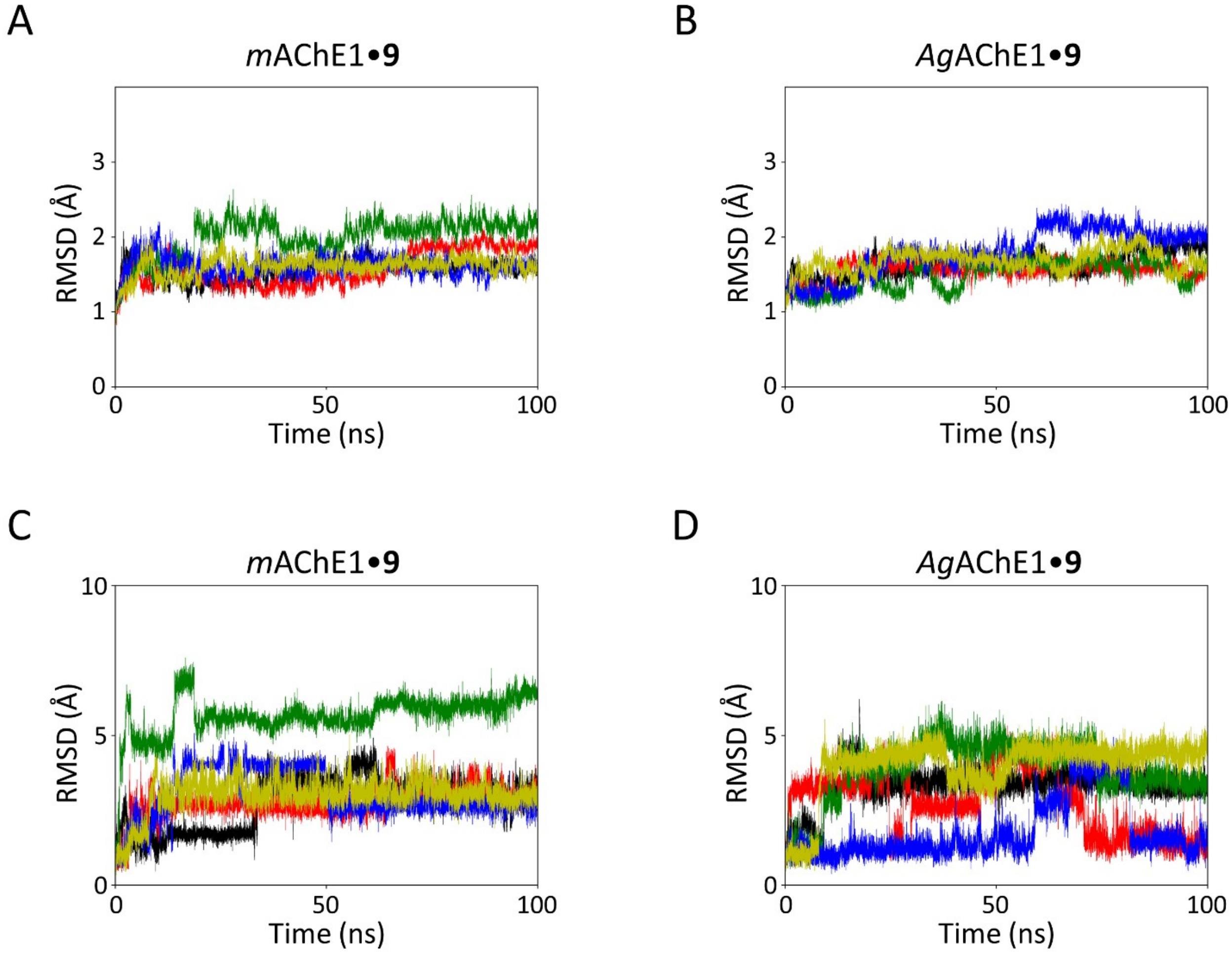

Figure S5. Root mean square deviation (RMSD) values vs simulation time for each MD simulation. (**A**) RMSD for protein mainchain atoms, for *m*AChE•**9**. (**B**) RMSD for protein mainchain atoms, for *Ag*AChE1•**9**. (**C**) RMSD for inhibitor **9** heavy atoms, for *m*AChE•**9**. (**D**) RMSD for inhibitor **9** heavy atoms, for *Ag*AChE1•**9**.

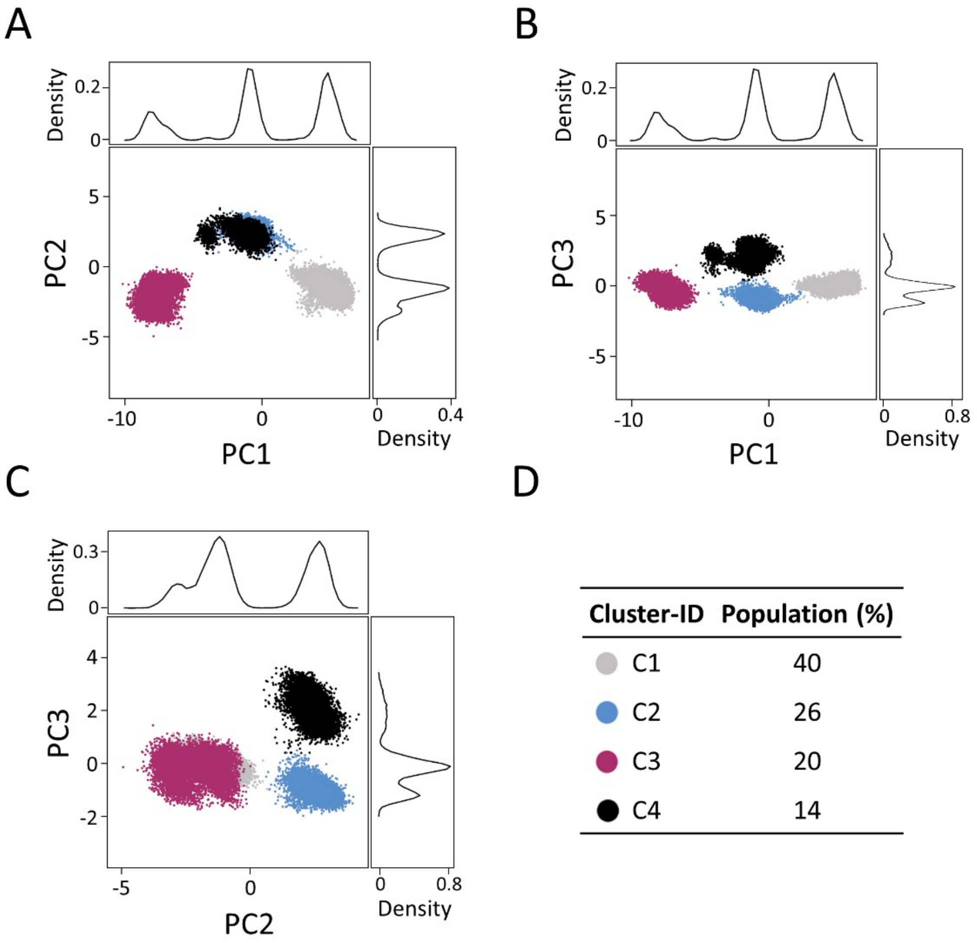

Cluster-ID	Population (%)
C1	40
C2	26
C3	20
C4	14

Figure S6. Projections of the first three PCs of the concatenated 50-100ns trajectories for *m*AChE•**9**, displaying the largest collective motion of the inhibitor **9** along the PCs. The different clusters in the PCA subspace, representing different binding conformations of the inhibitor **9**, are color coded. The density visualizes the relative population of conformations. (**A**) PC1 vs PC2. (**B**) PC1 vs PC3. (**C**) PC2 vs PC3. (**D**) Color code and population for the different clusters.

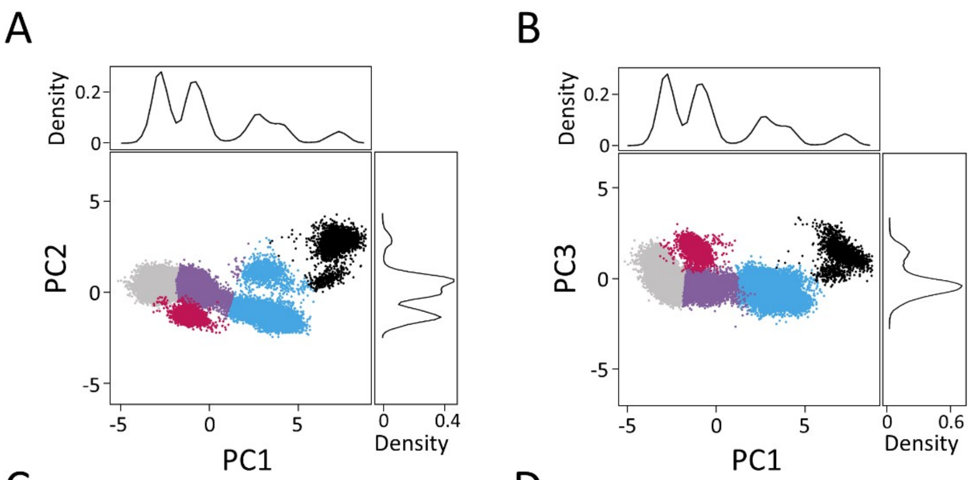

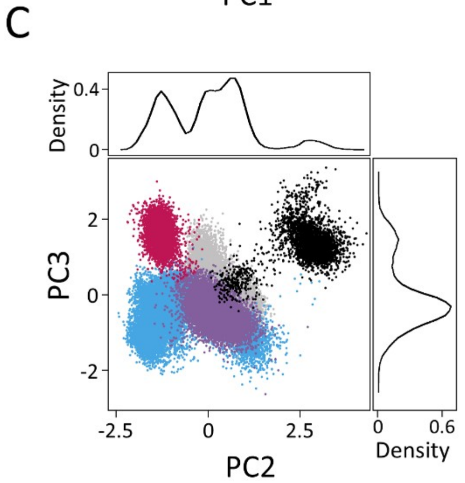

D

Cluster-ID	Population (%)
C1	34
C2	25
C3	24
C4	11
C5	6

Figure S7. Projections of the first three PCs of the concatenated 50-100ns trajectories for *Ag*AChE1•**9**, displaying the largest collective motion of the inhibitor **9** along the PCs. The different clusters in the PCA subspace, representing different binding conformations of the inhibitor **9**, are color coded. The density visualizes the relative population of conformations. (**A**) PC1 vs PC2. (**B**) PC1 vs PC3. (**C**) PC2 vs PC3. (**D**) Color code and population for the different clusters.

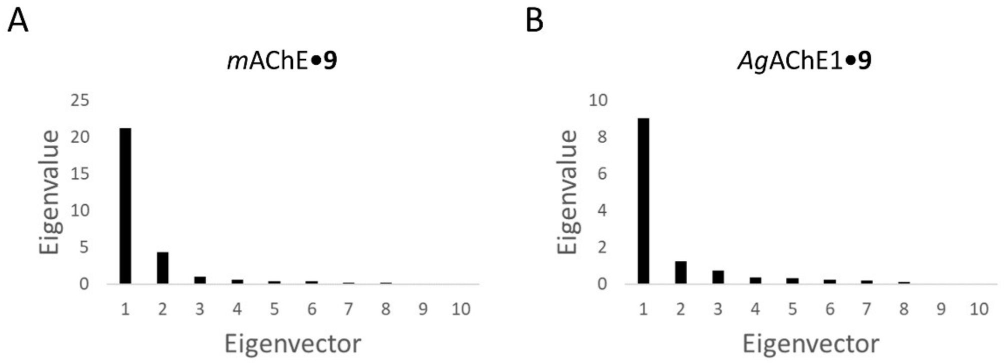

Figure S8. Eigenvalues for the first ten PCs (eigenvectors) for the PCA of heavy atoms of inhibitor **9**, over the concatenated 50-100 ns MD simulations for **A**) *m*AChE•**9**. **B**) *Ag*AChE1•**9**.

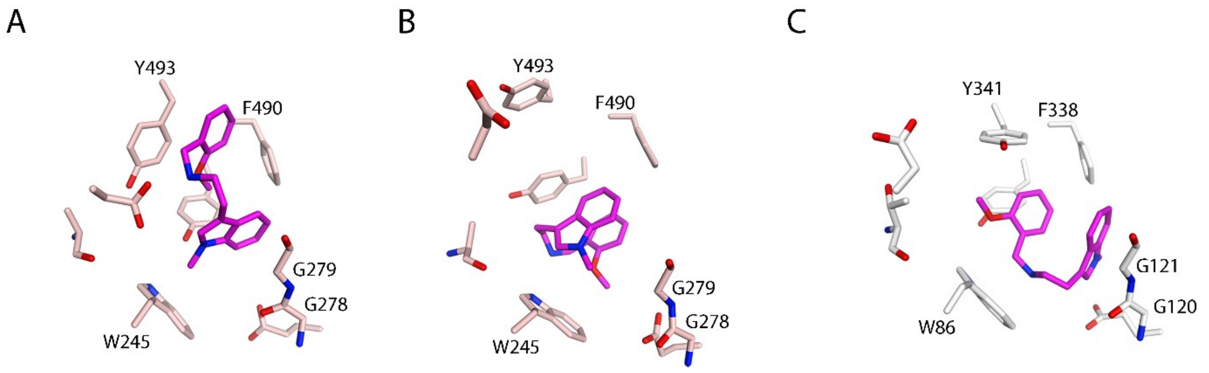

Figure S9. Representative binding poses of **9** in complex with *Ag*AChE1 and *m*AChE, selected based on cluster analysis of the inhibitors' conformations during the MD-simulations. (**A-B**) The centroid of clusters 4 and 5 of *Ag*AChE•**9** with populations of 11% and 6% of the full trajectory. (**C**) The centroid of cluster 4 of *m*AChE•**9** with a population of 14%. Amino acid residues identified as important for interactions with **9** are highlighted. The tyrosine residue in the center of the residues is Y489/Y337. The centroid inhibitor conformations of the three largest clusters are shown in Figure 6.

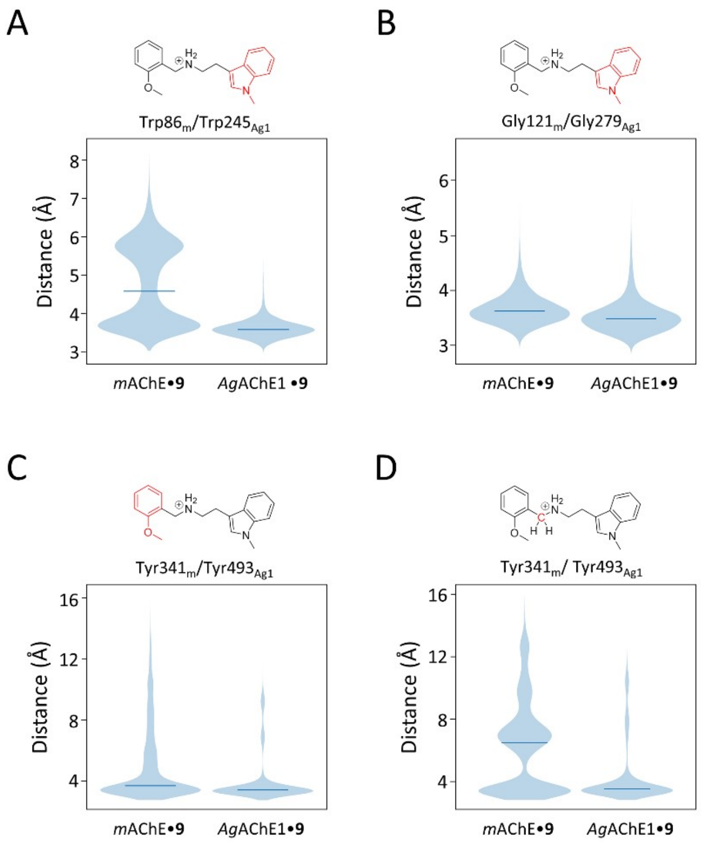

Figure S10. Pairwise minimum distances measured between the indicated atoms of inhibitor **9** (marked in red) and heavy atoms of the side chain of selected amino acids. Distances were calculated for the concatenated 50-100 ns MD simulations. The dark blue line indicates the mean value of the minimum distance, and the width of the light blue shape represents the fraction of frames at each distance. (**A**) Distance between the indole moiety of **9** and $Trp86_m/Trp245_{Ag1}$. (**B**) Distance between the indole moiety of **9** and $Gly121_m/Gly279_{Ag1}$. (**C**) Distance between the methoxy benzyl moiety of **9** and $Tyr341_m/Tyr493_{Ag1}$. (**D**) Distance between one α-carbon to the secondary amine in the linker of **9** and $Tyr341_m/Tyr493_{Ag1}$.

Table S4. Water occupancy at hydrogen bonding distances (< 3 Å) to selected heavy atoms of inhibitor **9**, for concatenated 50-100 ns MD simulations of *m*AChE•**9** and *Ag*AChE1•**9**. The average number of water molecules are reported.

	**Water occupancy**		
	**Methoxy benzyl (any atom)**	**Indole (any atom)**	**Linker (nitrogen)**
*m*AChE•**9**	0.07	0.18	0.89
*Ag*AChE1•**9**	0.06	0.11	0.41

In vivo raw data

Table S5. In vivo raw data for compound **8** as insecticide towards *Ae. aegypti* at a dose of 2, 1, 0.5, 0.2 and 0.02 nmol/mosquito.

Compound	Dose (nmol)	No. Mosquitoes (*Aa*)	24h Mortality[a]	% 24 Mortality[b]	48h Mortality[a]	% 48 Mortality[b]
**Acetone**	**Control**	50	1	2.00	1	2.00
**8**	2	100	58	58.00	63	63.00
**8**	1	100	31	31.00	39	39.00
**8**	0.5	100	9	9.00	12	12.00
**8**	0.2	100	2	2.00	4	4.00

[a,b] Average value from of three different experiments (triplicate).

Table S6. In vivo raw data for compound **9** as insecticide towards *Ae. aegypti* at a dose of 2, 1, 0.5, 0.2 and 0.02 nmol/mosquito.

Compound	Dose (nmol)	No. Mosquitoes (*Aa*)	24h Mortality[a]	% 24 Mortality[b]	48h Mortality[a]	% 48 Mortality[b]
**Acetone**	**Control**	50	1	2.00	1	2.00
**9**	2	100	100	100.00	100	100.00
**9**	1	100	100	100.00	100	100.00
**9**	0.5	100	90	90.00	90	90.00
**9**	0.2	100	45	45.00	54	54.00
**9**	0.02	100	2	2.00	3	3.00

[a,b] Average value from of three different experiments (triplicate).

Table S7. In vivo raw data for compounds **16** and **18** as insecticide towards *An. gambiae* at a dose of 2 and 0.2 nmol/mosquito.

Compound	Dose (nmol)	No. Mosquitoes (*Ag*)	24h Mortality[a]	% 24 Mortality[b]	48h Mortality[a]	% 48 Mortality[b]
**Acetone**	**Control**	50	2	4.00	2	4.00
**16**	2	98	98	100.00	98	100.00
**16**	0.2	100	50	50.00	53	53.00
**18**	2	100	100	100.00	100	100.00
**18**	0.2	90	41	45.56	43	47.78

[a,b] Average value from of three different experiments (triplicate).

Table S8. In vivo raw data for compounds **16** and **18** as insecticide towards *Ae. aegypti* at a dose of 2 and 0.2 nmol/mosquito.

Compound	Dose (nmol)	No. Mosquitoes (*Aa*)	24h Mortality[a]	% 24 Mortality[b]	48h Mortality[a]	% 48 Mortality[b]
**Acetone**	**Control**	50	2	4.00	2	4.00
**16**	2	98	98	100.00	98	100.00
**16**	0.2	100	6	12.00	6	12.00
**18**	2	100	100	100.00	100	100.00
**18**	0.2	50	2	4.00	2	4.00

[a,b] Average value from of three different experiments (triplicate).

Table S9. In vivo raw data for propoxur insecticide (positive control) towards *An. gambiae* at a dose of 0.02-0.00002 nmol/mosquito.

Compound	Dose (nmol)	No. Mosquitoes (*Ag*)	24 Mortality[a]	% 24 Mortality[b]	48 Mortality[a]	% 48 Mortality[b]
**Propoxur**	0.02	115	115	100.00	115	100.00
**Propoxur**	0.002	129	100	77.52	108	83.72
**Propoxur**	0.0002	88	4	4.55	6	6.82
**Propoxur**	0.00002	150	1	0.67	1	0.67

[a,b] Average value from of three different experiments (triplicate).

Table S10. In vivo raw data for propoxur insecticide (positive control) towards *Ae. aegypti* at a dose of 0.02-0.00002 nmol/mosquito.

Compound	Dose (nmol)	No. Mosquitoes (*Aa*)	24 Mortality[a]	% 24 Mortality[b]	48 Mortality[a]	% 48 Mortality[b]
**Propoxur**	0.02	115	115	99.00	115	99.00
**Propoxur**	0.002	129	100	73.52	108	83.72
**Propoxur**	0.0002	88	4	1.69	6	1.7
**Propoxur**	0.00002	150	1	0.67	1	0.67

[a,b] Average value from of three different experiments (triplicate).

NMR spectra of intermediates

(Tert-butyl (2-(1H-indol-3-yl)ethyl)carbamate) (3a)

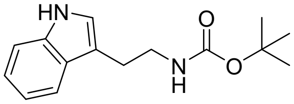

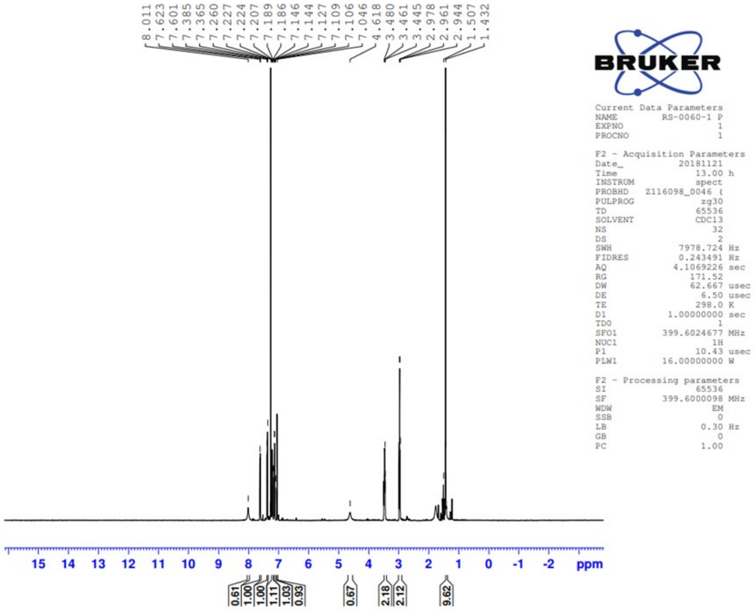

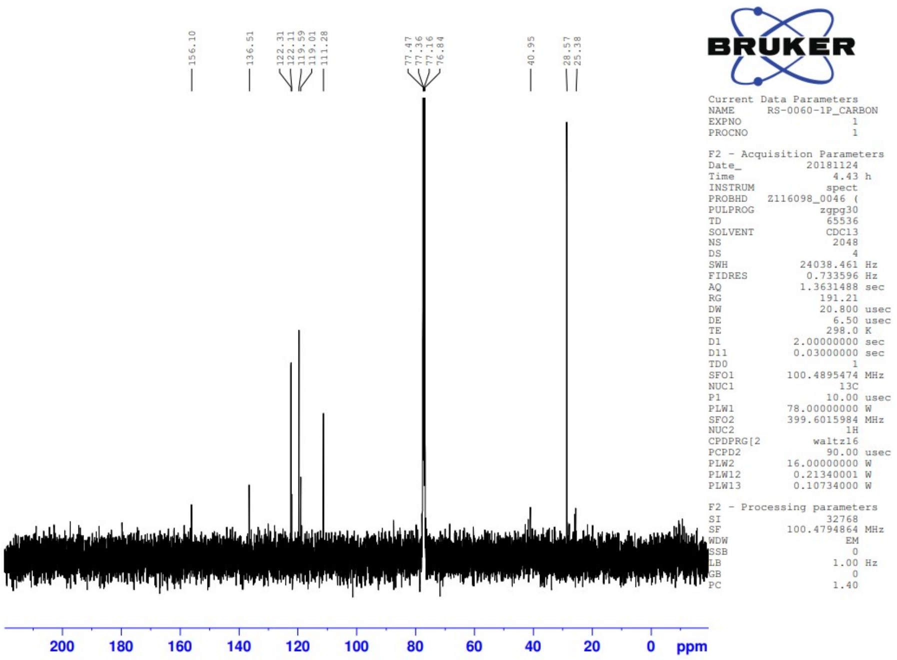

Tert-butyl (2-(5-chloro-1H-indol-3-yl)ethyl)carbamate (3b).

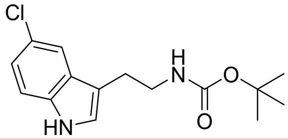

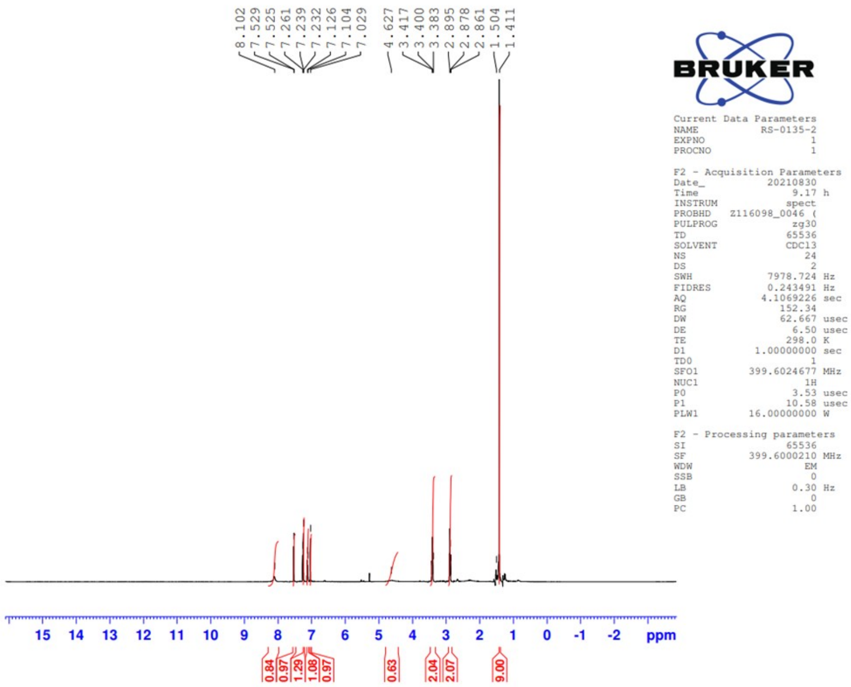

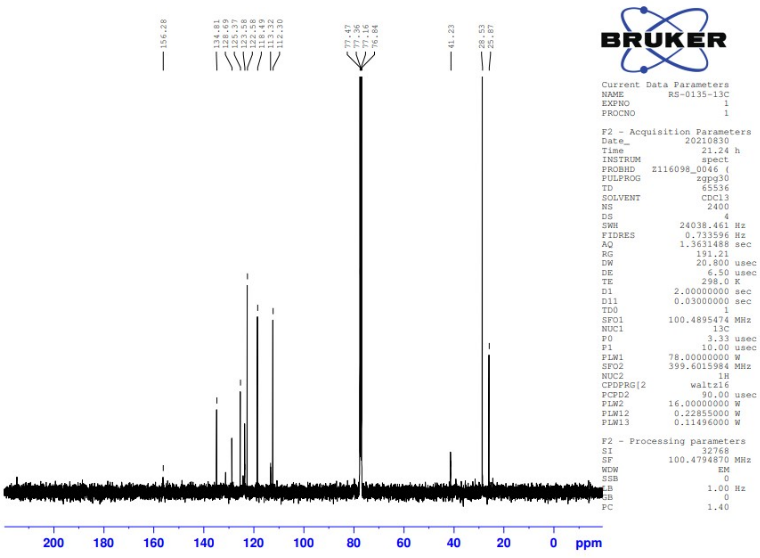

Tert-butyl (2-(5-methyl-1H-indol-3-yl)ethyl)carbamate (3c)

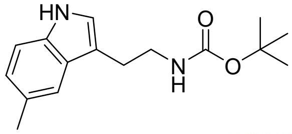

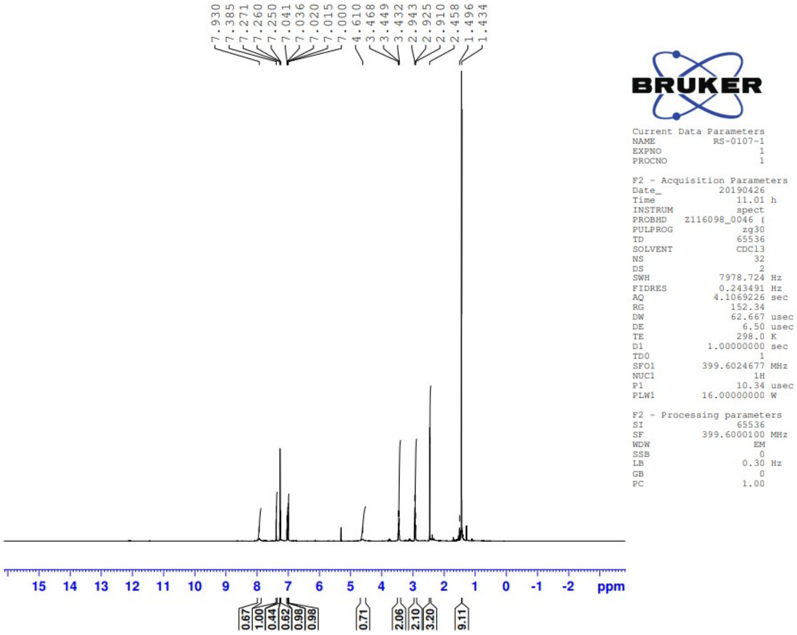

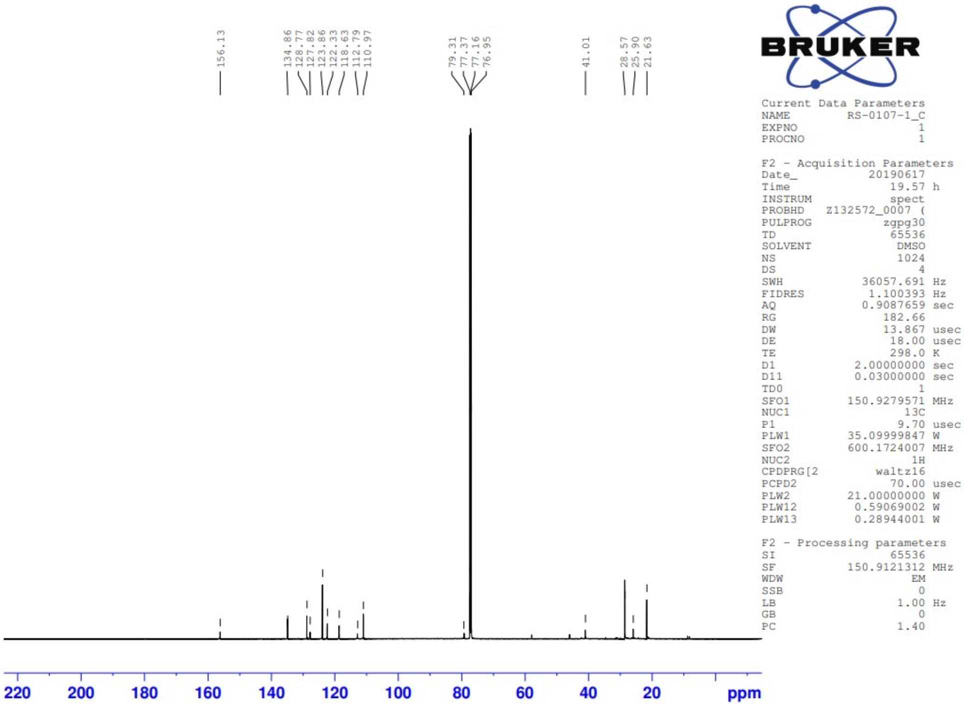

Tert-butyl (2-(6-fluoro-1H-indol-3-yl)ethyl)carbamate (3d).

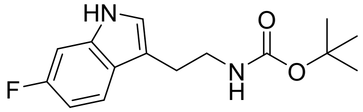

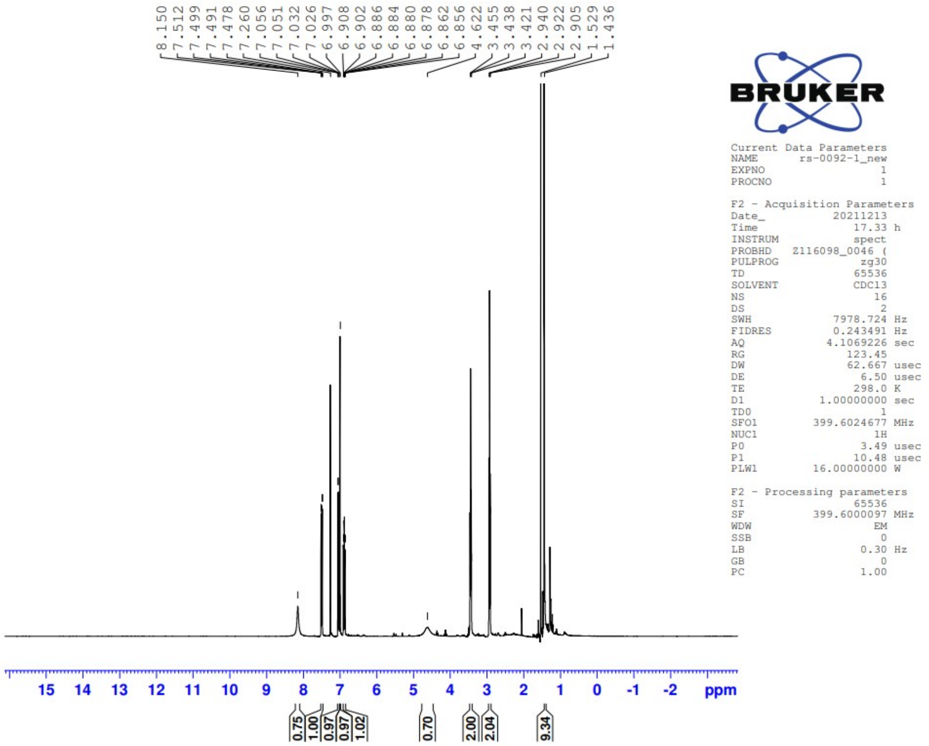

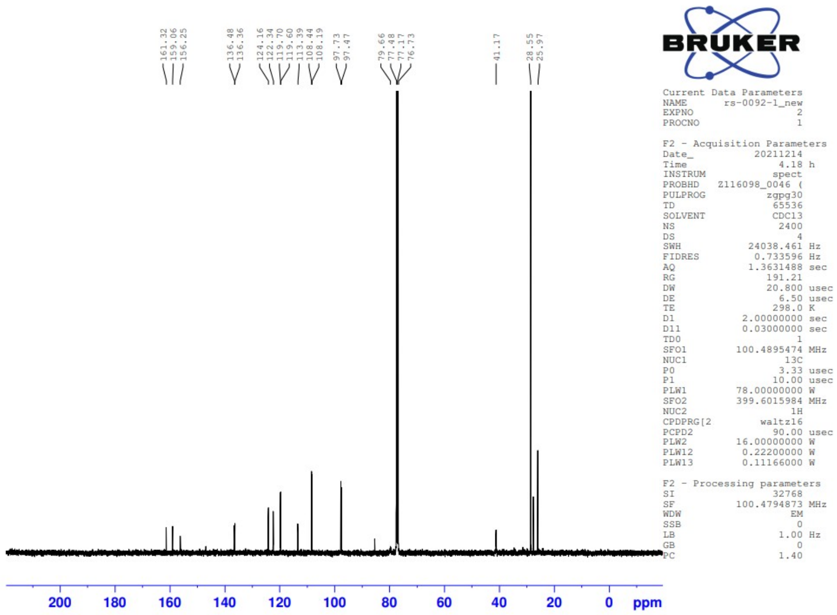

Tert-butyl (2-(5-methoxy-1H-indol-3-yl)ethyl)carbamate (3e).

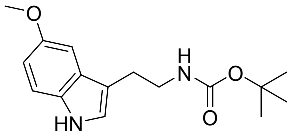

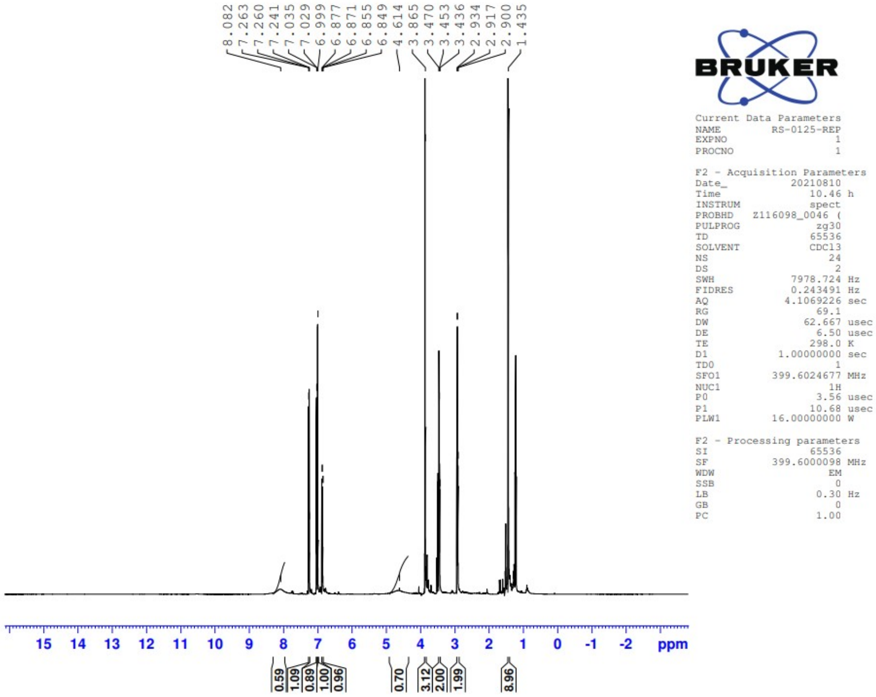

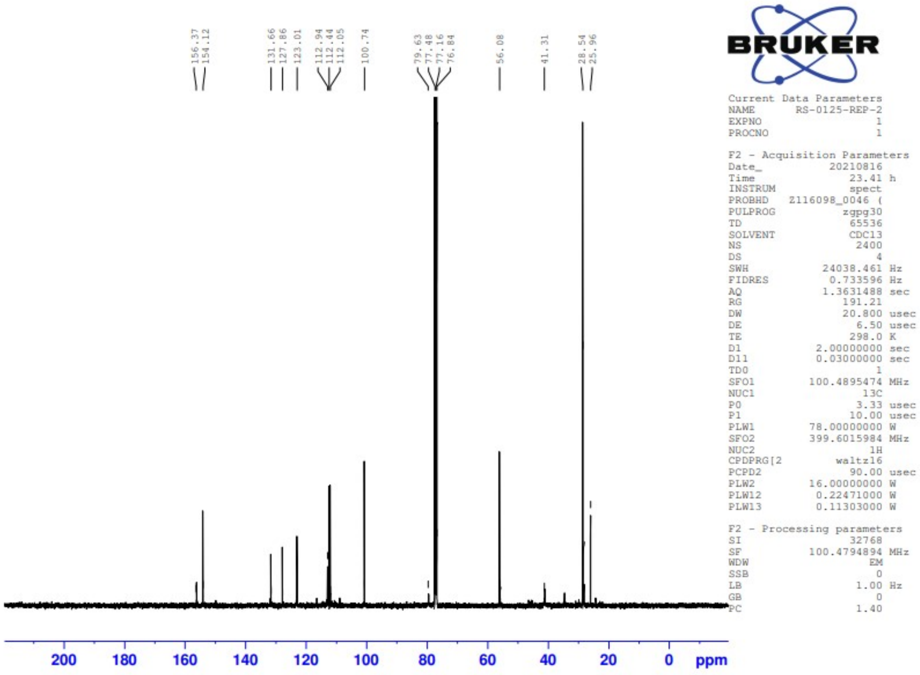

(Tert-butyl (2-(1-methyl-1H-indol-3-yl)ethyl)carbamate) (4a)

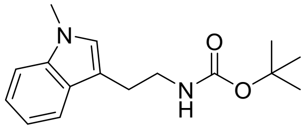

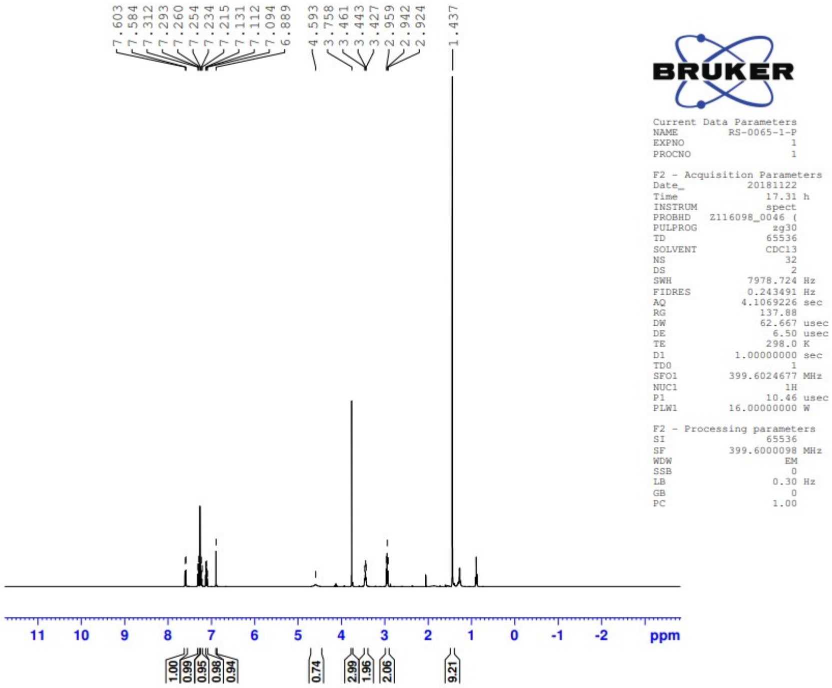

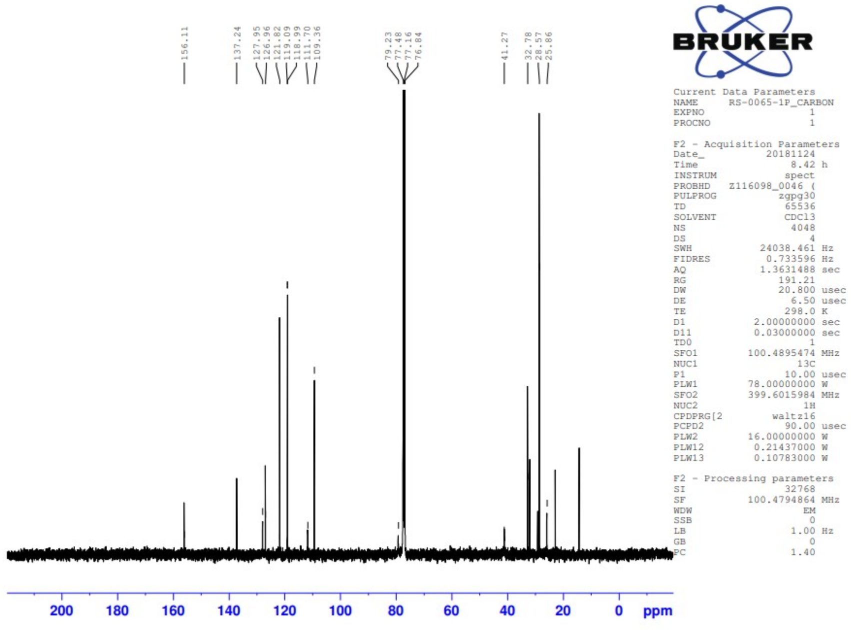

Tert-butyl (2-(5-chloro-1-methyl-1H-indol-3-yl)ethyl)carbamate (4b)

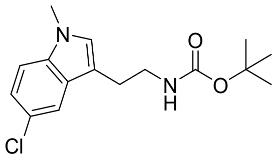

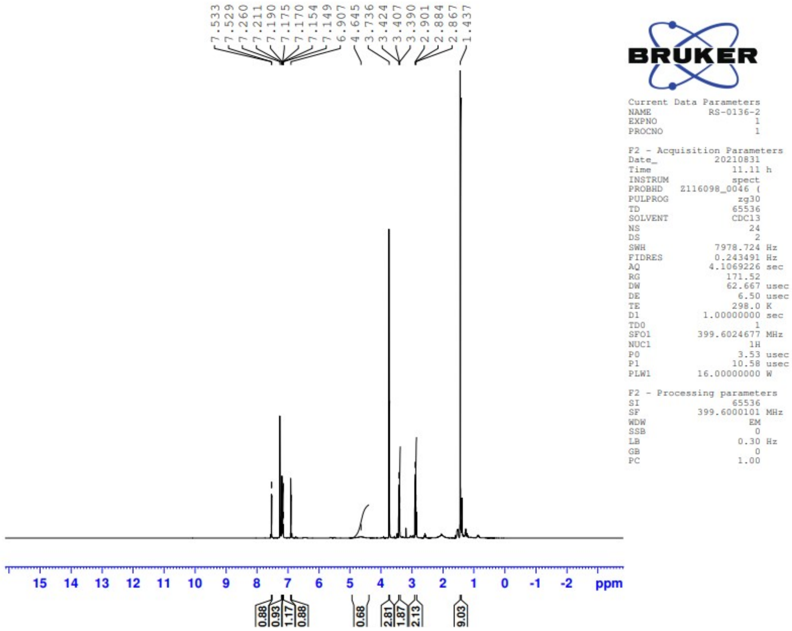

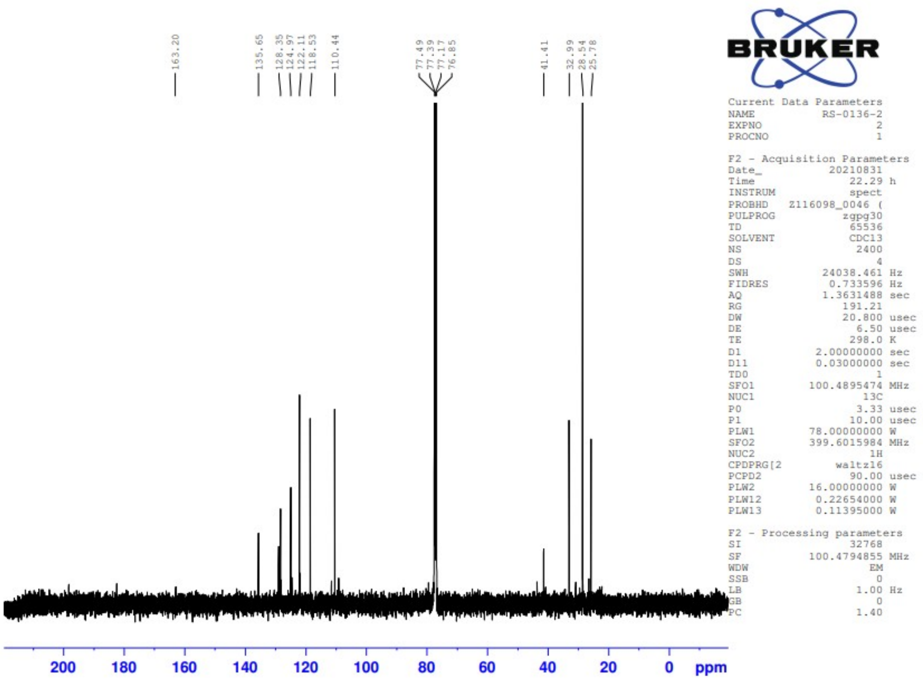

Tert-butyl (2-(5-methyl-1H-indol-3-yl)ethyl)carbamate (4c)

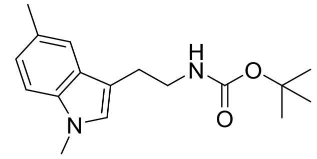

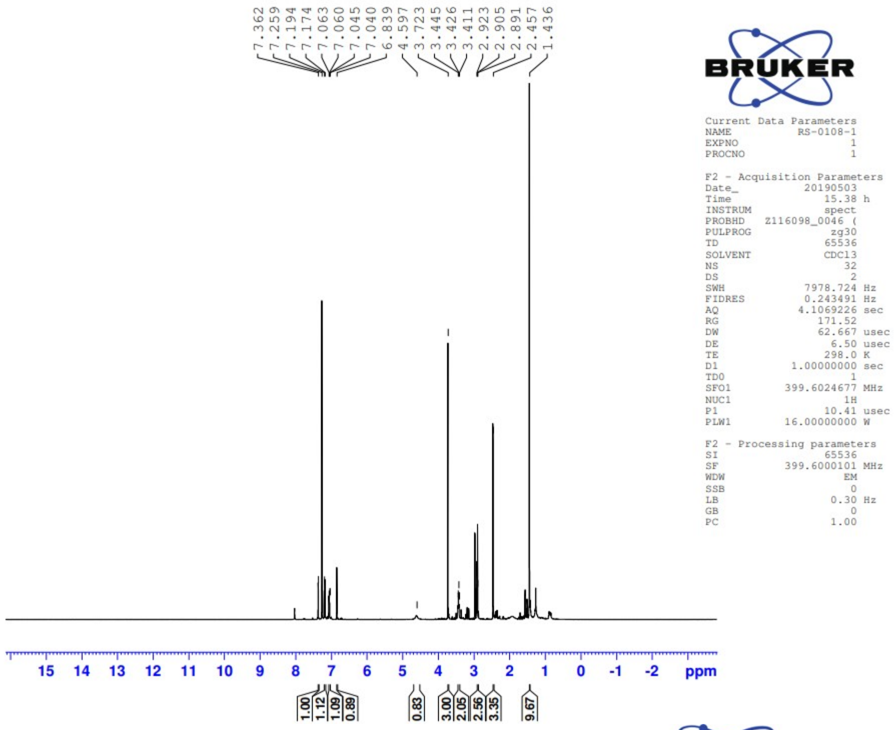

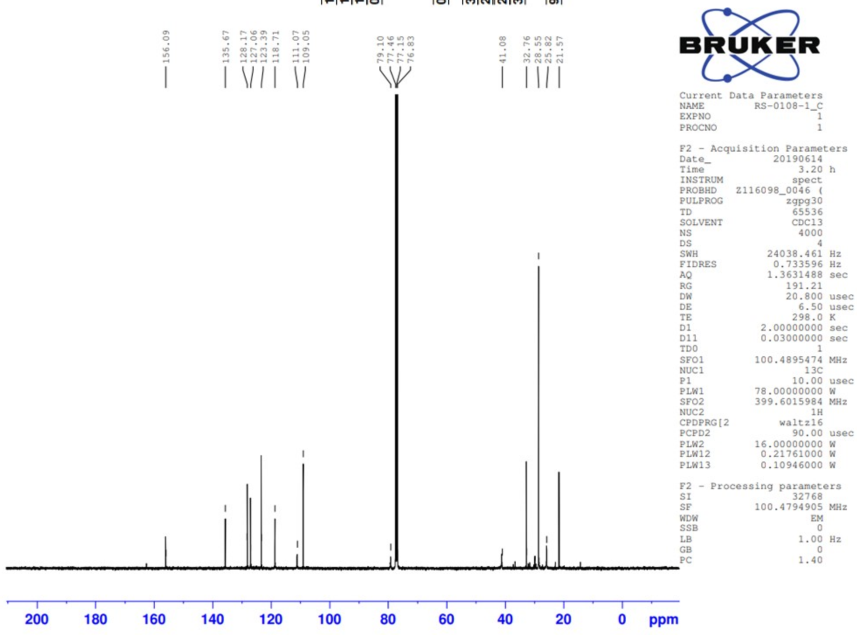

Tert-butyl (2-(6-fluoro-1-methyl-1H-indol-3-yl)ethyl)carbamate (4d).

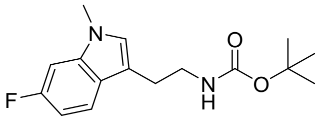

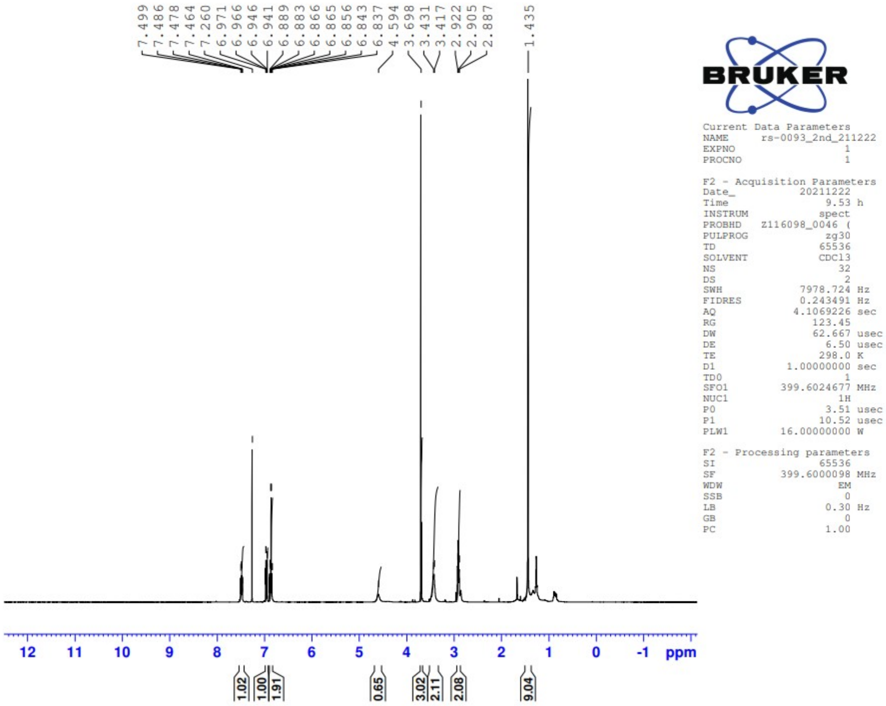

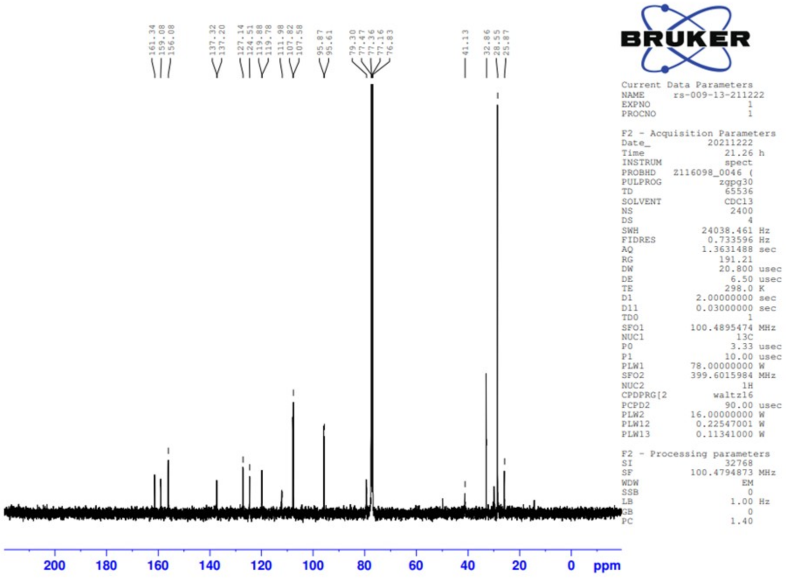

Tert-butyl (2-(5-methoxy-1-methyl-1H-indol-3-yl)ethyl)carbamate (4e)

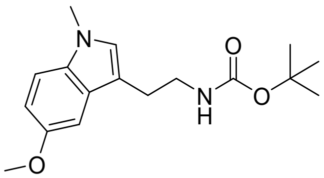

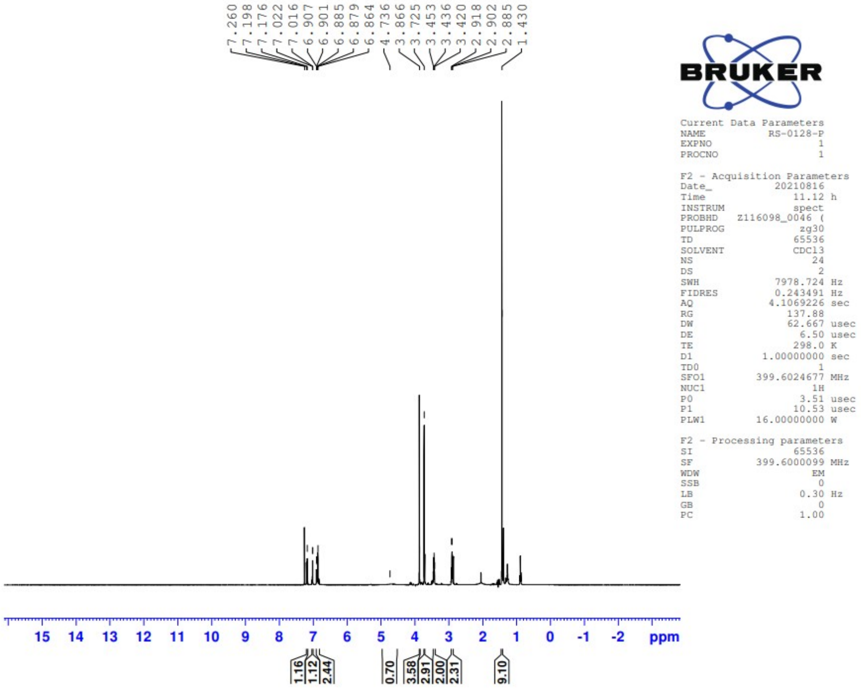

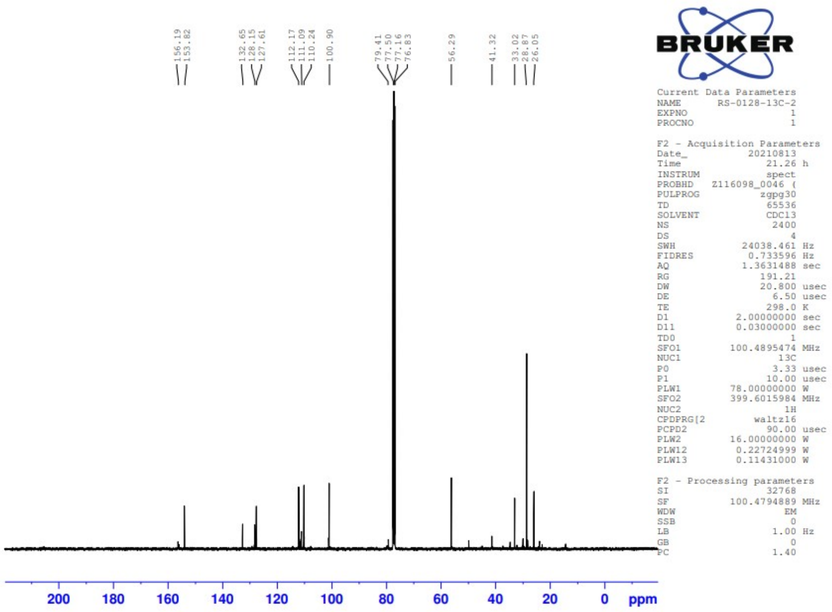

Tert-butyl methyl(2-(1-methyl-1H-indol-3-yl)ethyl)carbamate (27)

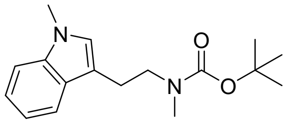

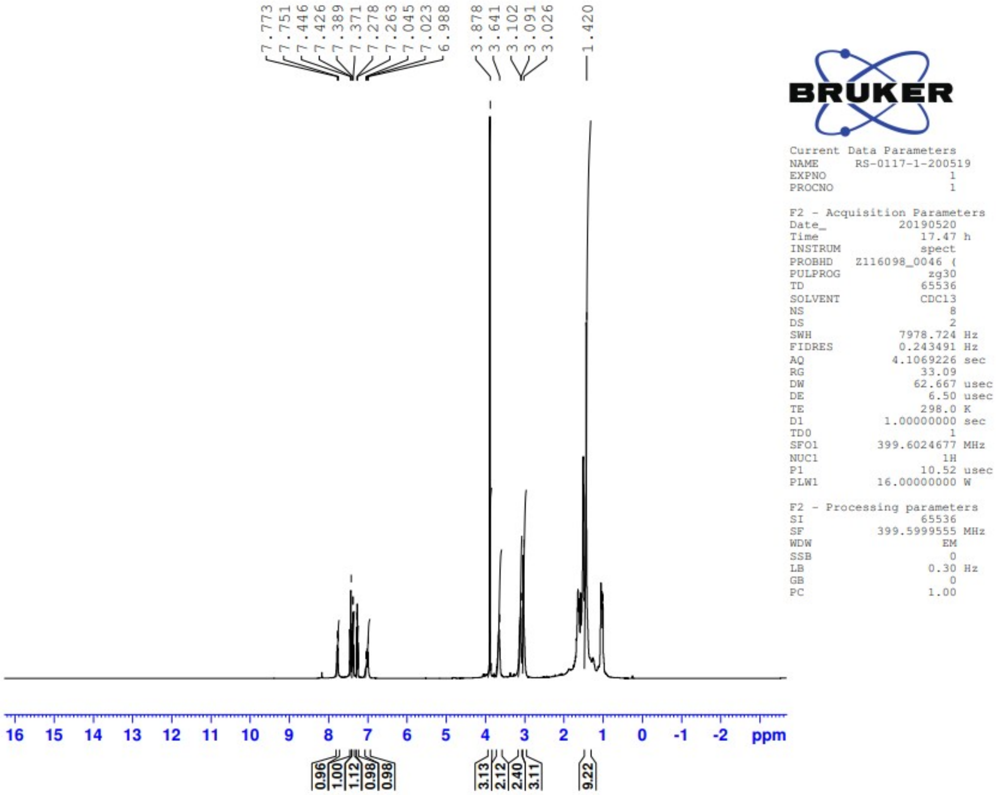

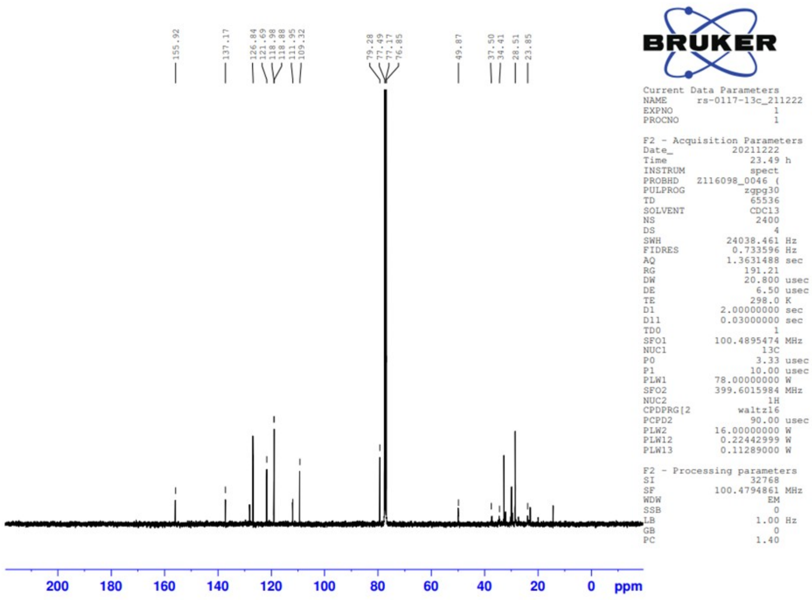

2-(1-methyl-1H-indol-3-yl)ethan-1-amine (5a)

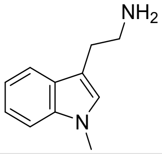

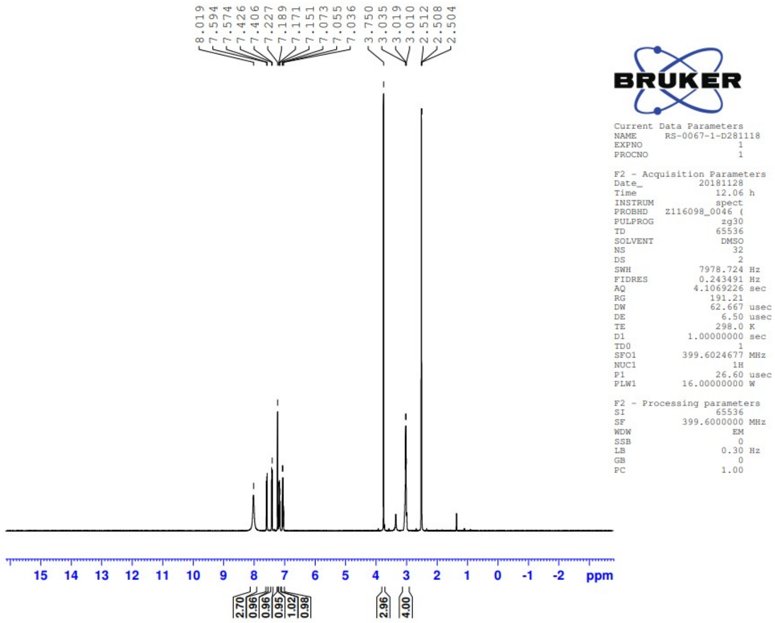

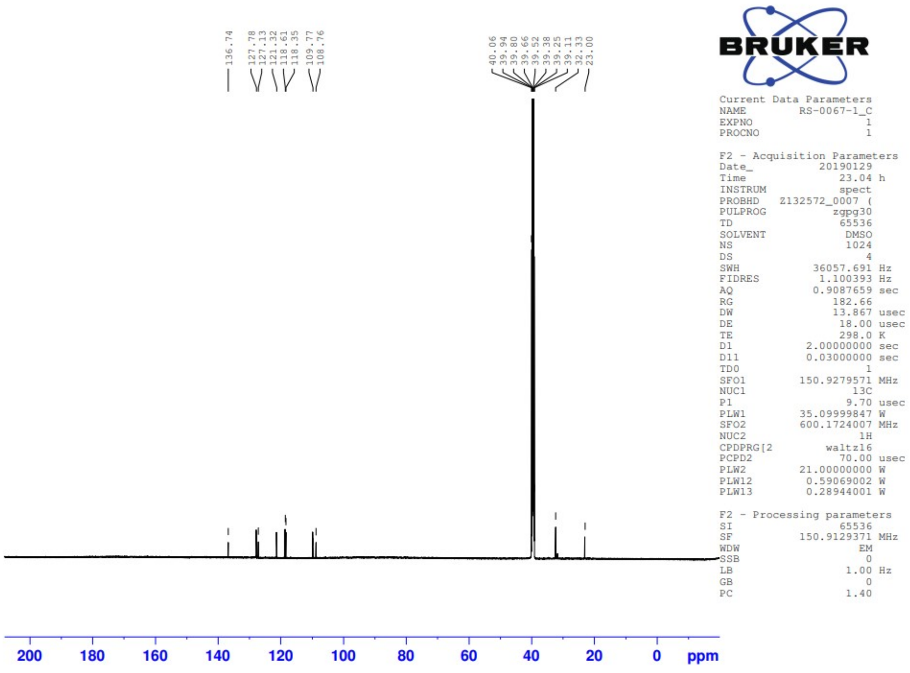

2-(5-chloro-1-methyl-1H-indol-3-yl)ethan-1-amine (5b).

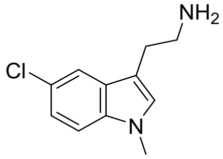

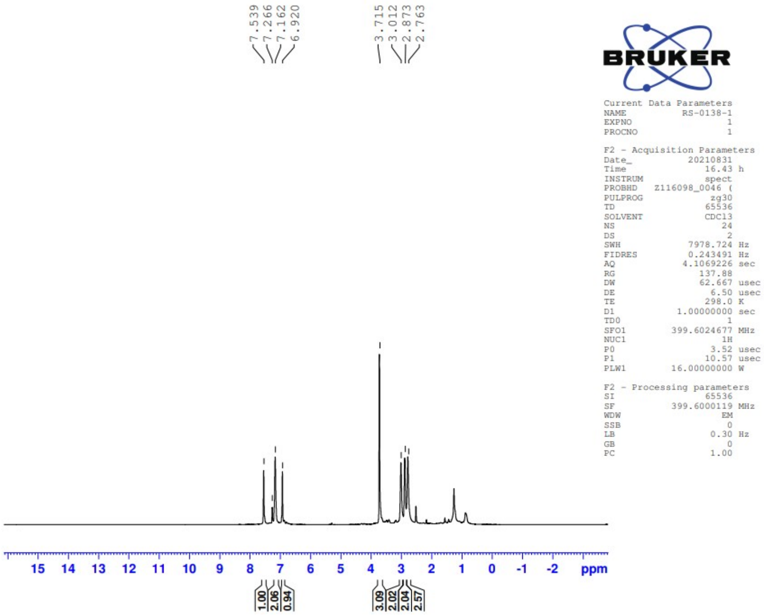

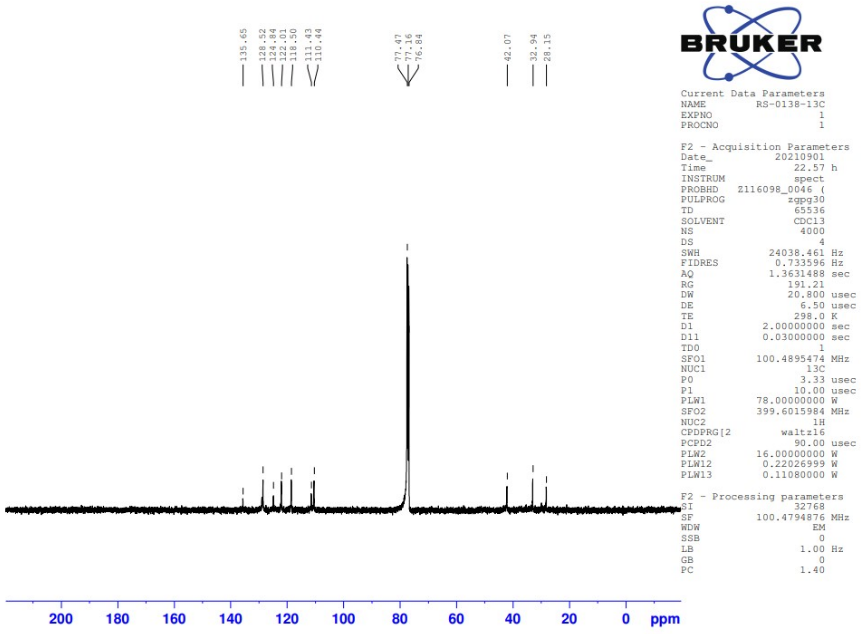

2-(1,5-dimethyl-1H-indol-3-yl)ethan-1-amine (5c).

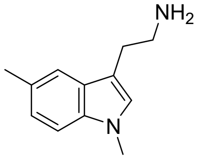

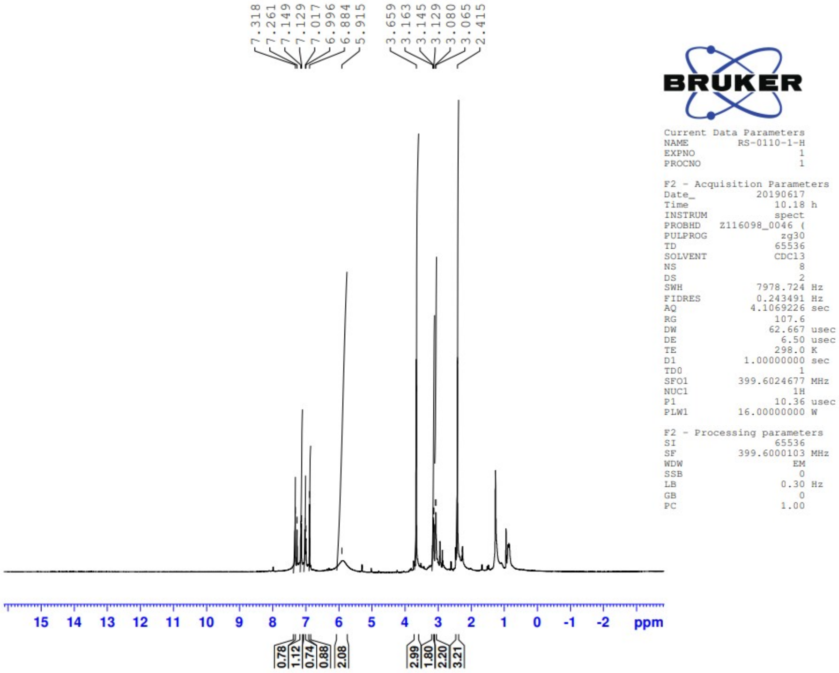

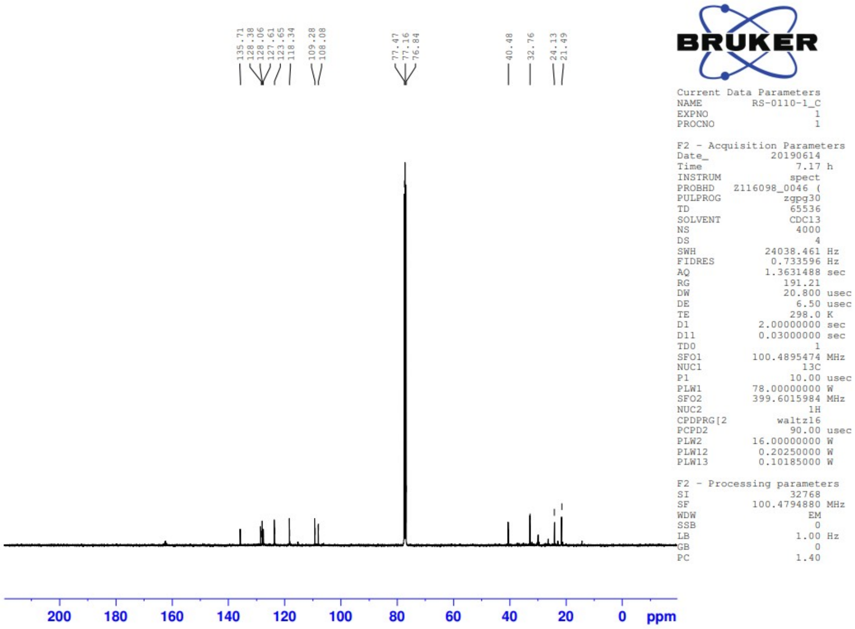

2-(6-fluoro-1-methyl-1H-indol-3-yl)ethan-1-amine (5d).

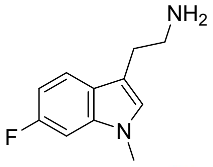

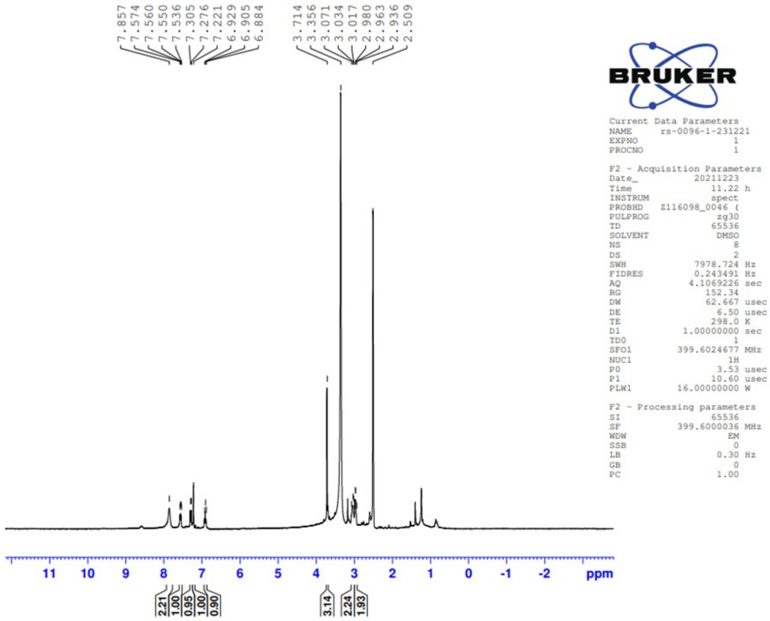

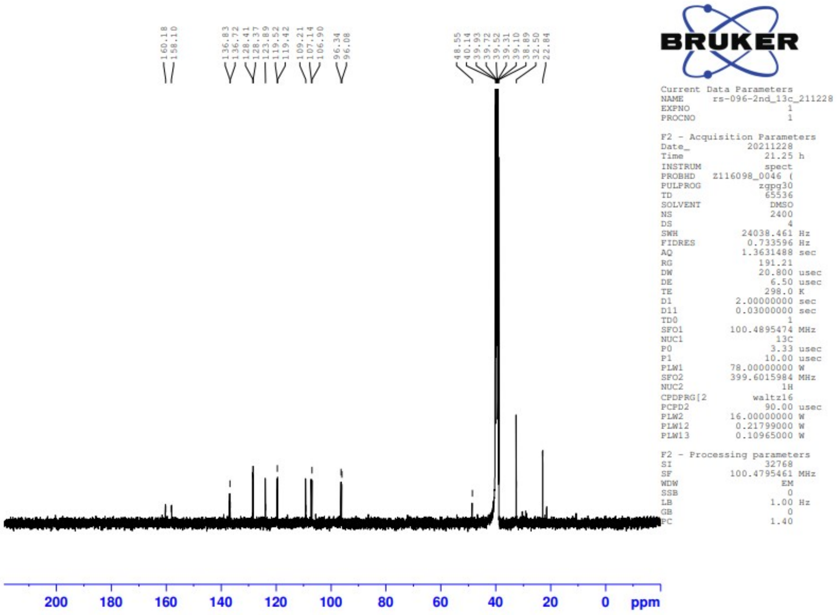

2-(5-methoxy-1-methyl-1H-indol-3-yl)ethan-1-amine (5e)

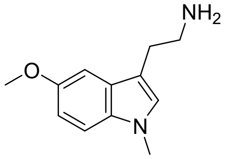

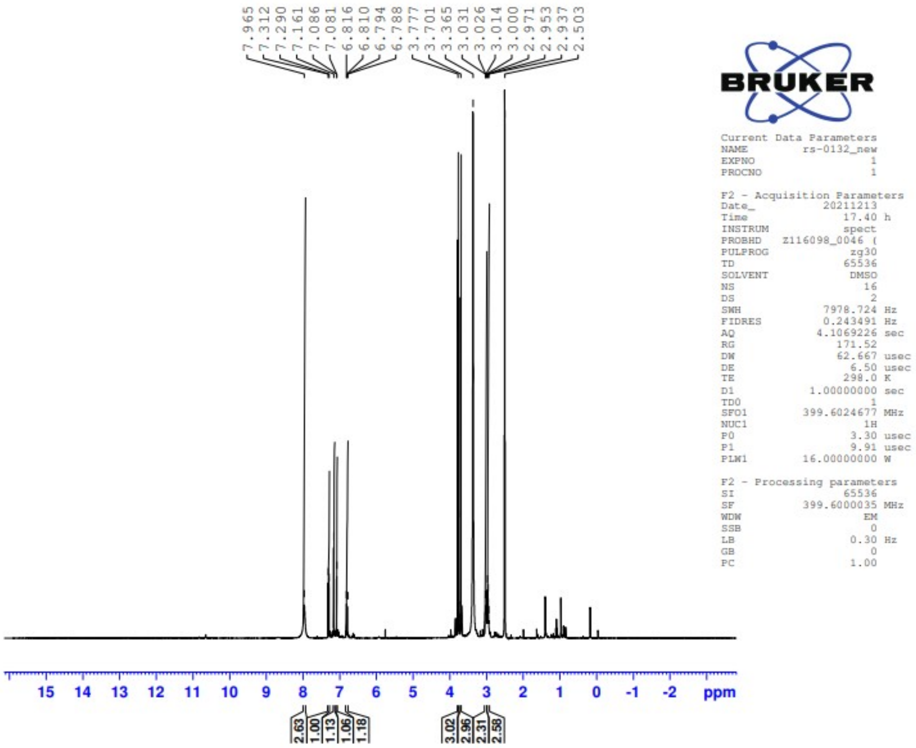

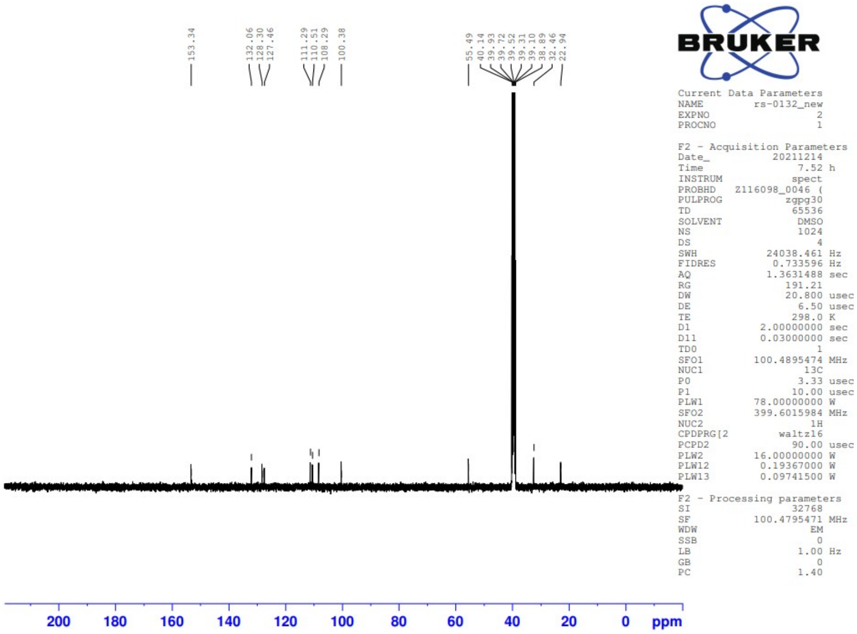

3-(2-aminoethyl)-1-methyl-1H-indol-5-ol (5f)

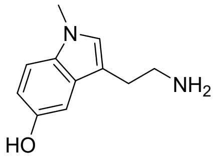

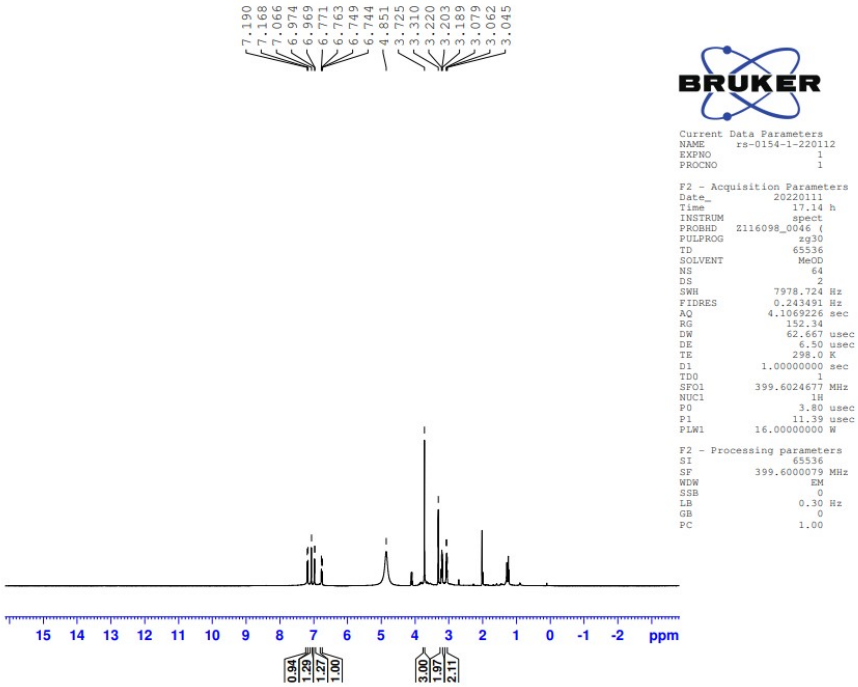

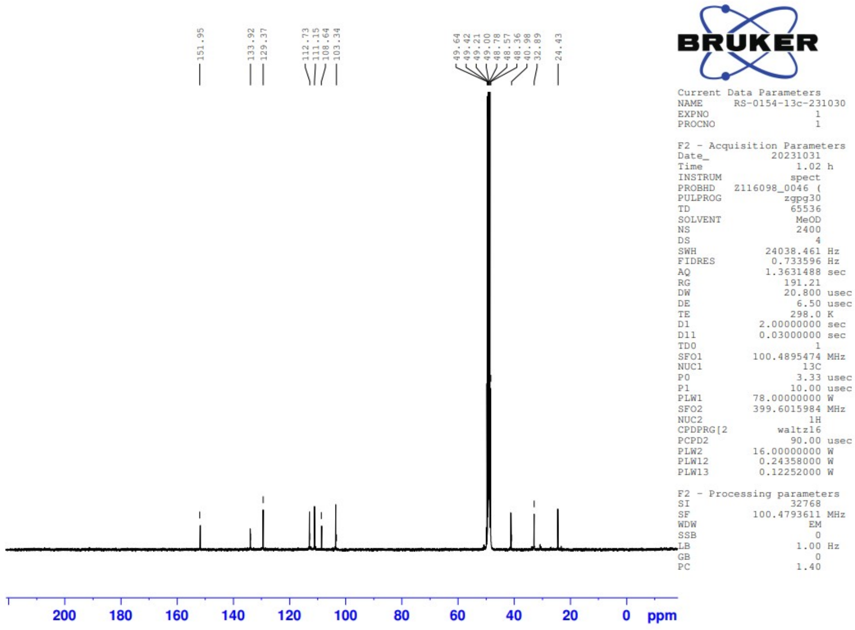

N-methyl-2-(1-methyl-1H-indol-3-yl)ethan-1-amine (28)

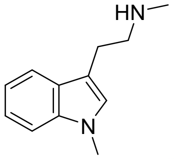

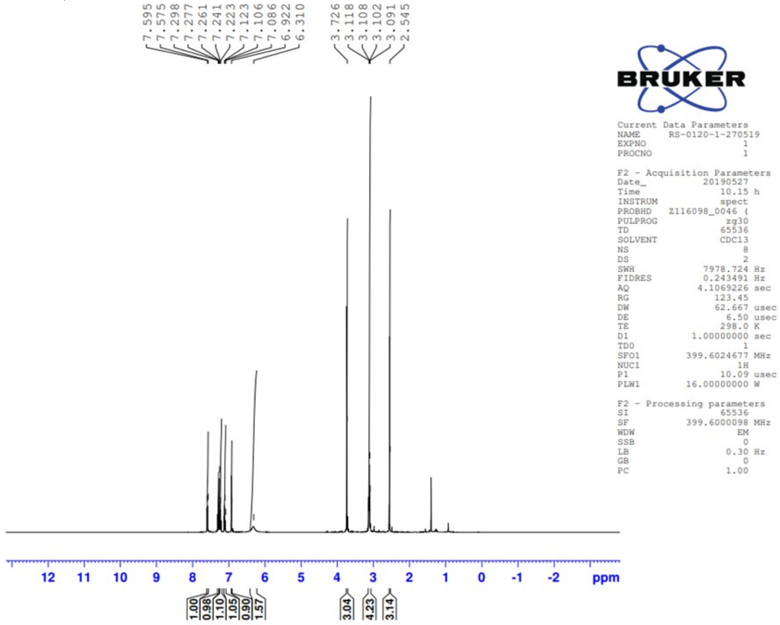

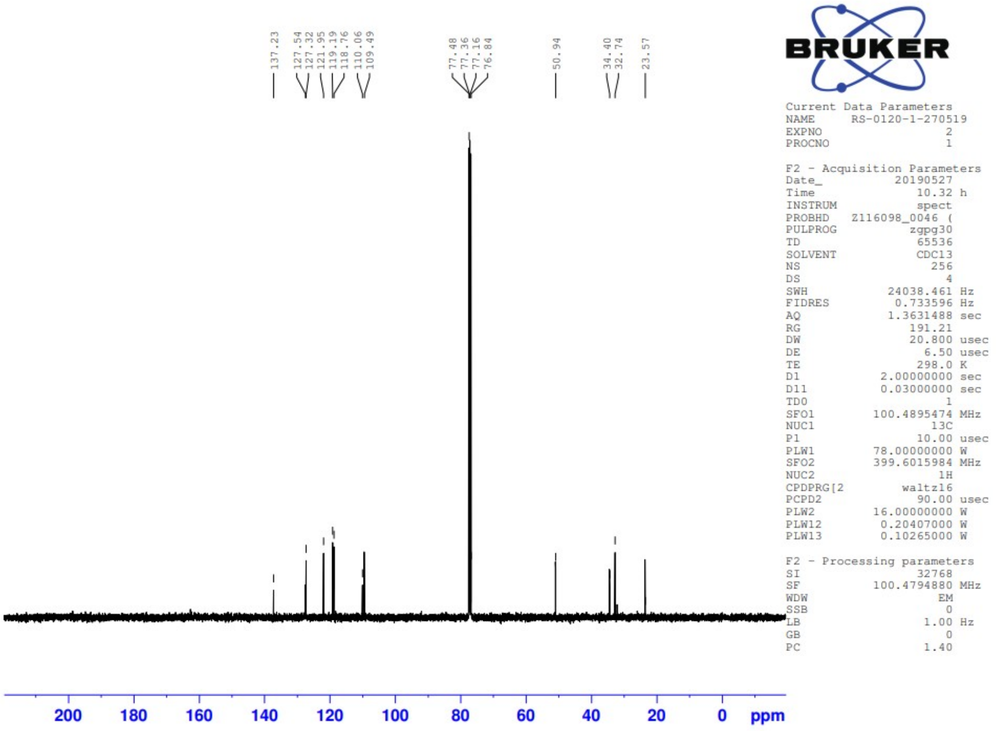

NMR spectra of final compounds

N-benzyl-2-(1H-indol-3-yl)ethan-1-amine HCl (6)

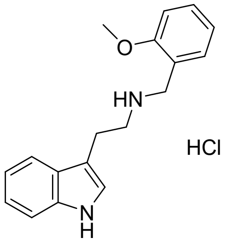

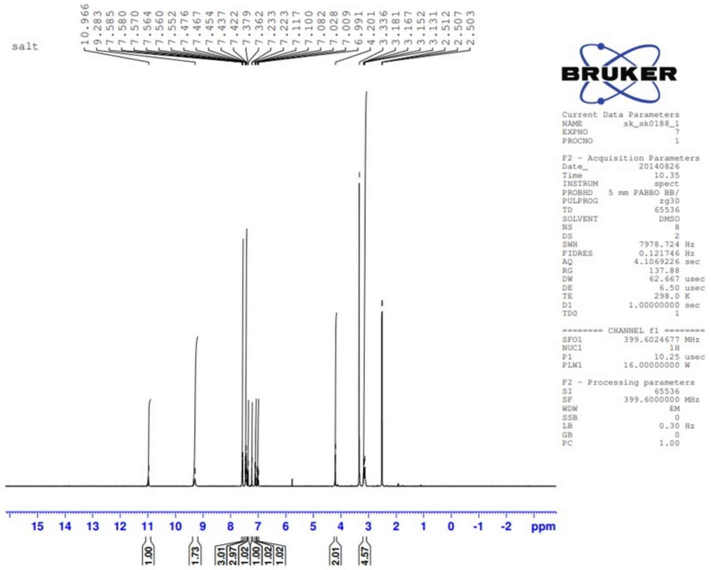

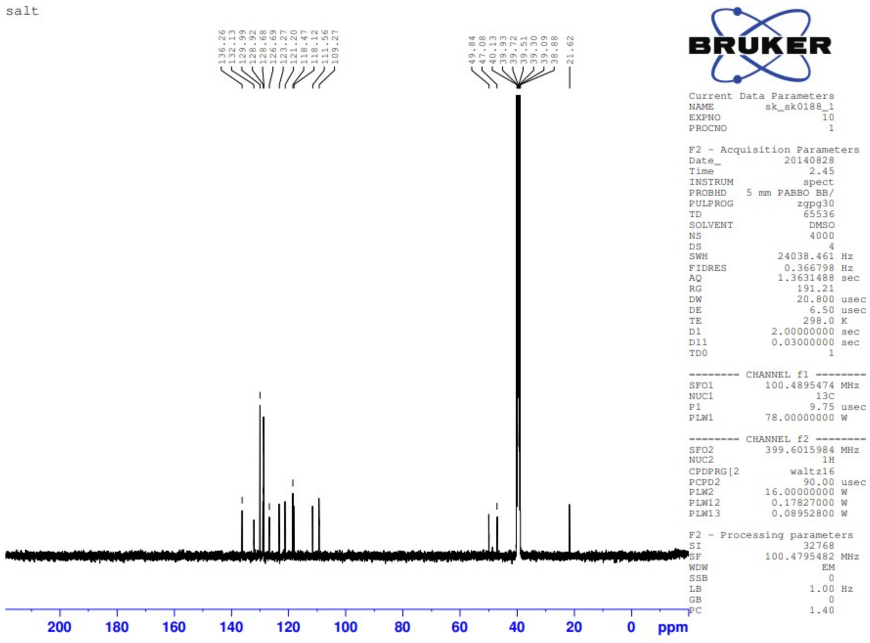

benzyl-2-(1-methyl-1H-indol-3-yl)ethan-1-amine HCl (7).

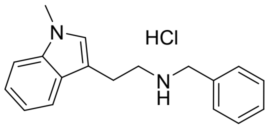

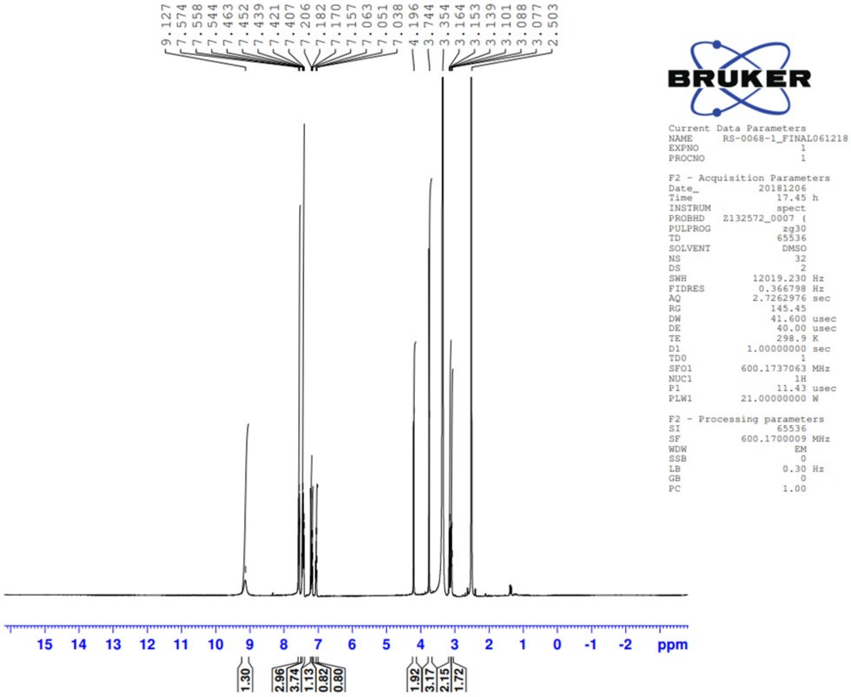

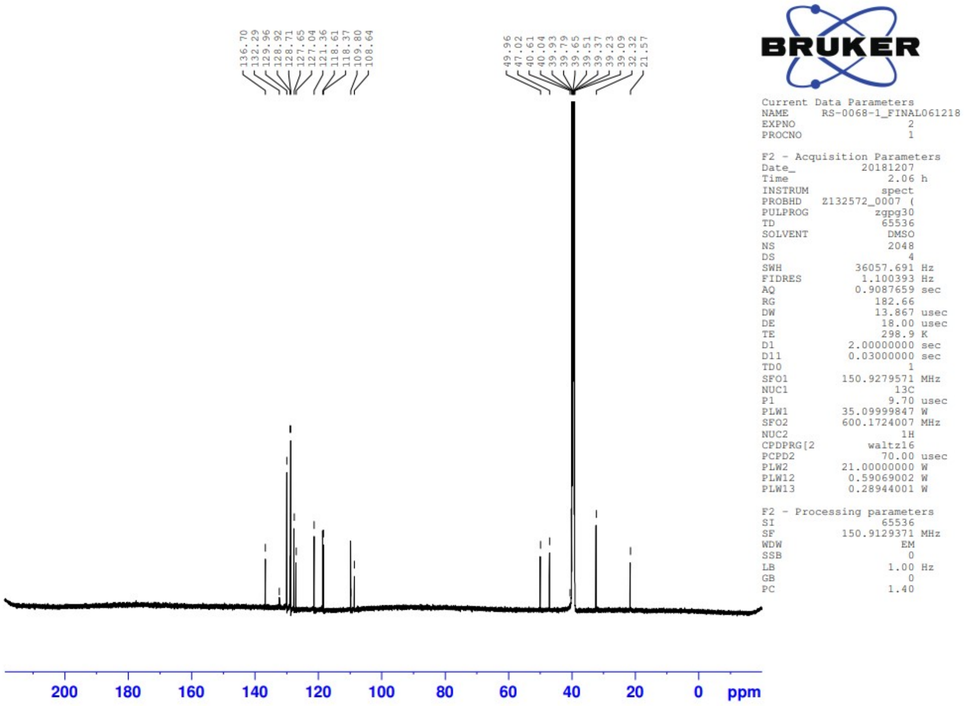

2-(1H-indol-3-yl)-N-(2-methoxybenzyl)ethan-1-amine HCl (8).

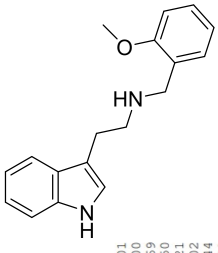

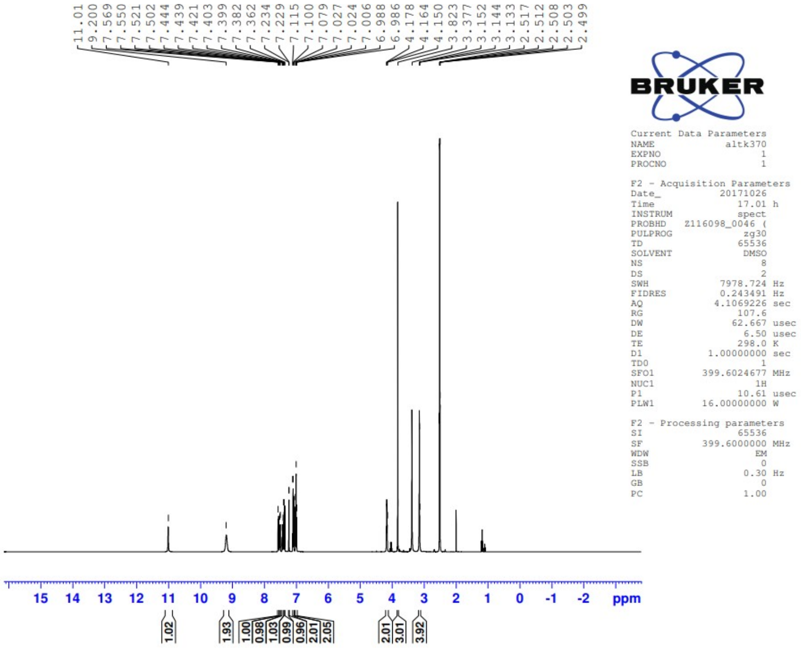

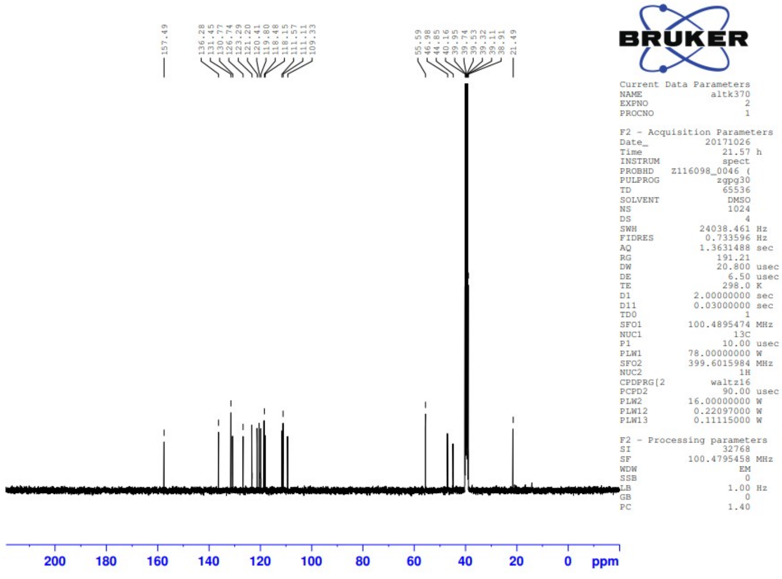

N-(2-methoxybenzyl)-2-(1-methyl-1H-indol-3-yl)ethan-1-amine HCl (9).

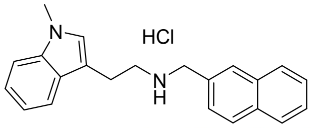

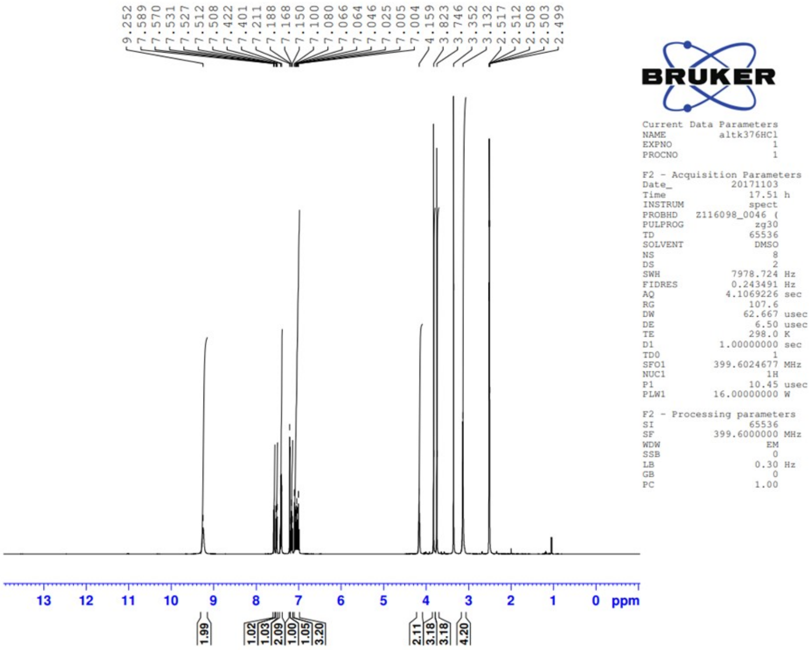

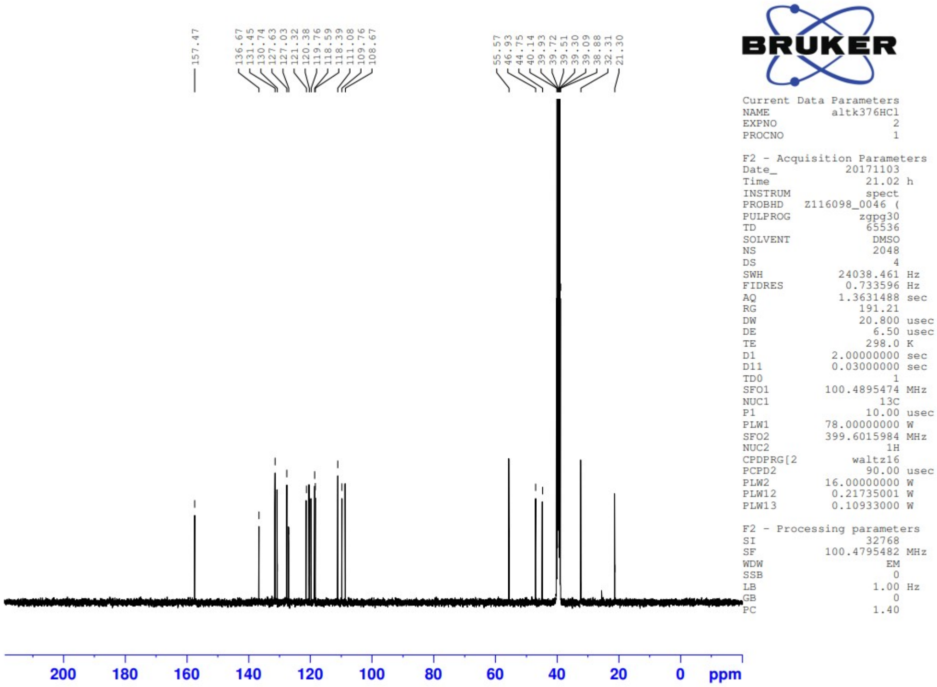

2-(1H-indol-3-yl)-N-(4-nitrobenzyl)ethan-1-amine HCl (10)

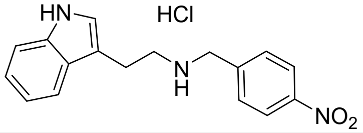

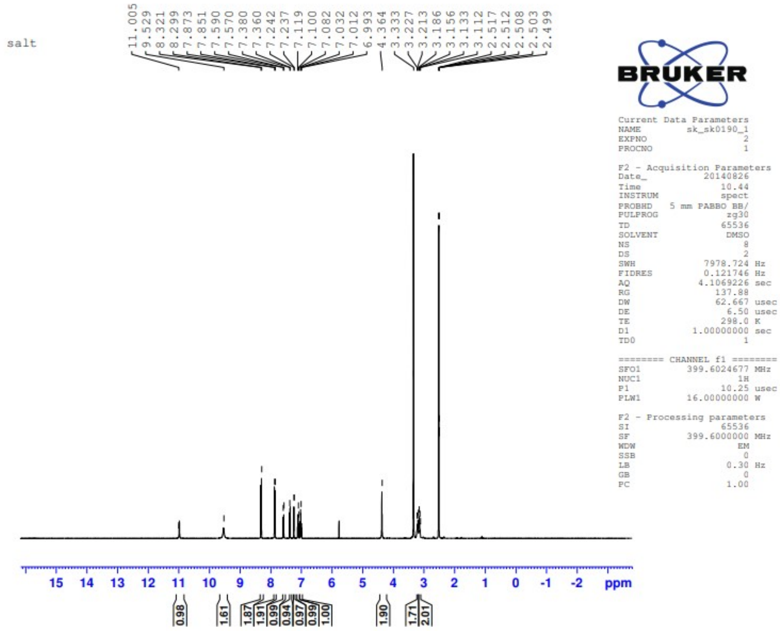

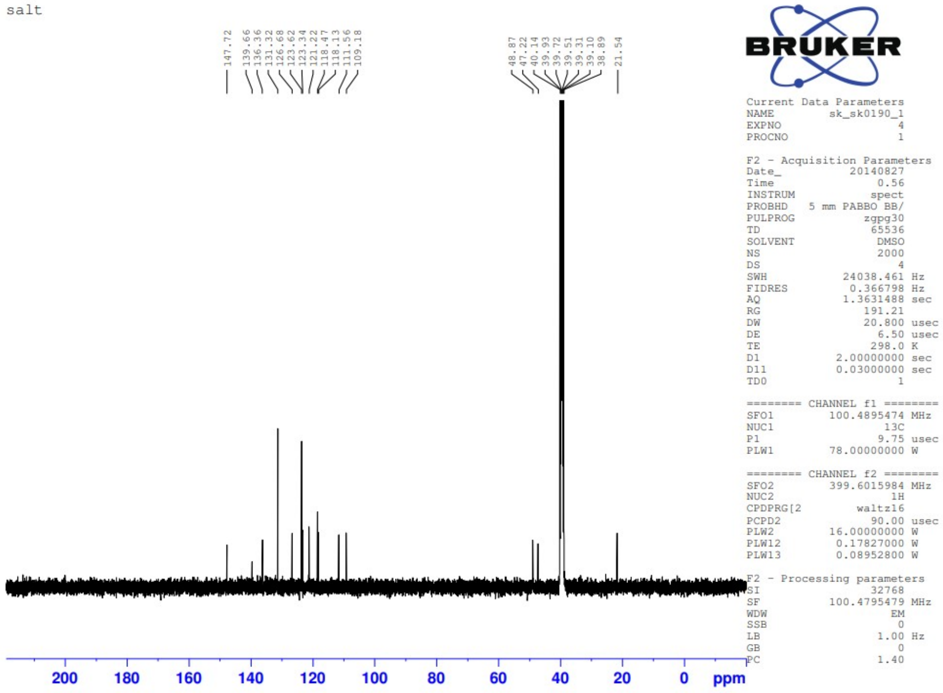

2-(1-methyl-1H-indol-3-yl)-N-(4-nitrobenzyl)ethan-1-amine HCl (11).

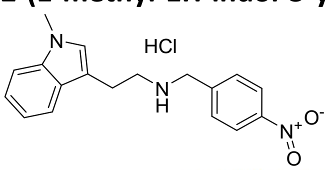

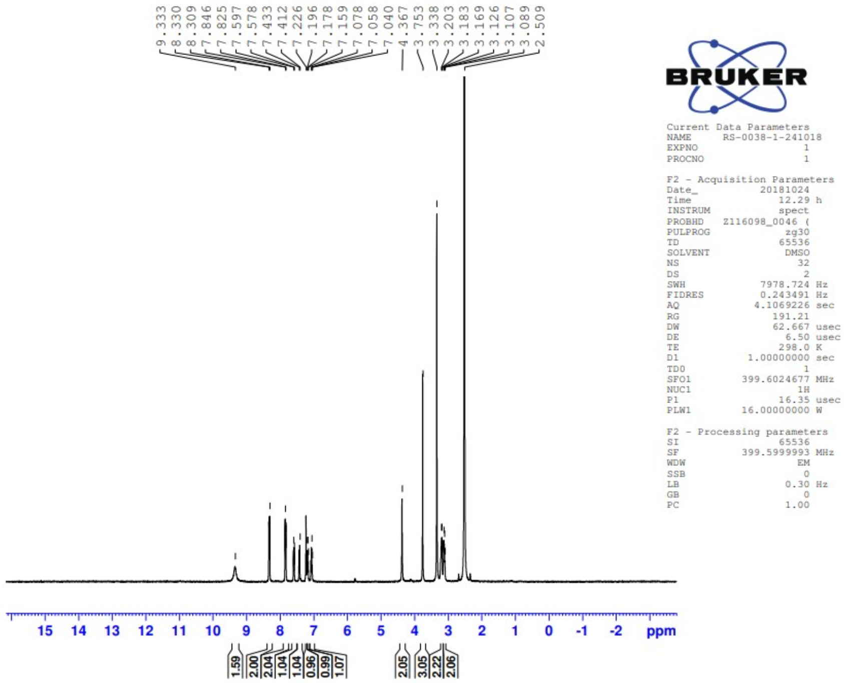

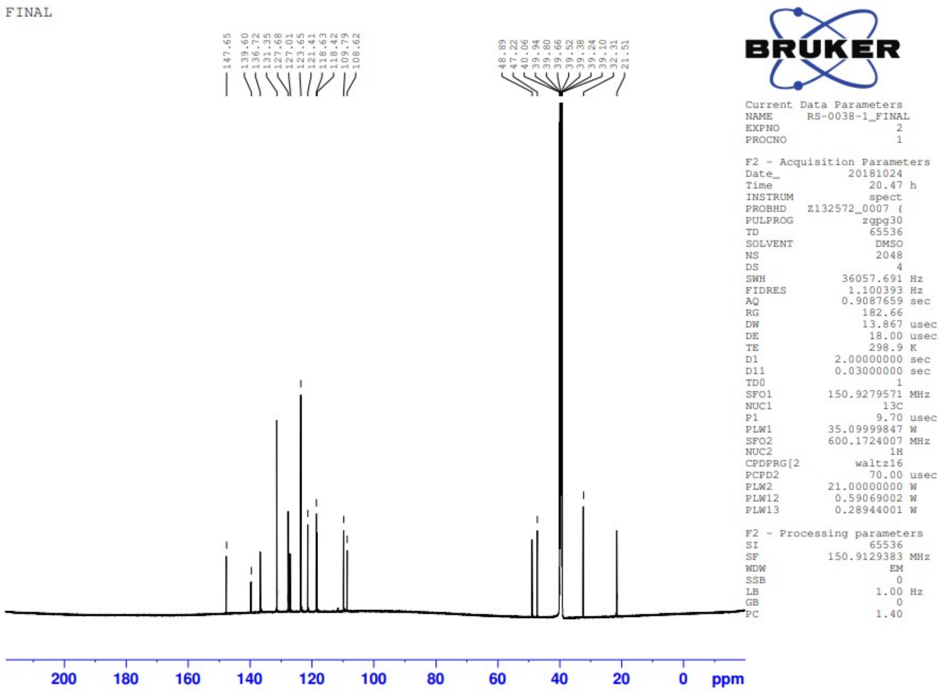

N-(4-chlorobenzyl)-2-(1H-indol-3-yl)ethan-1-amine HCl (12).

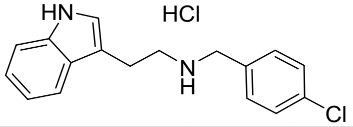

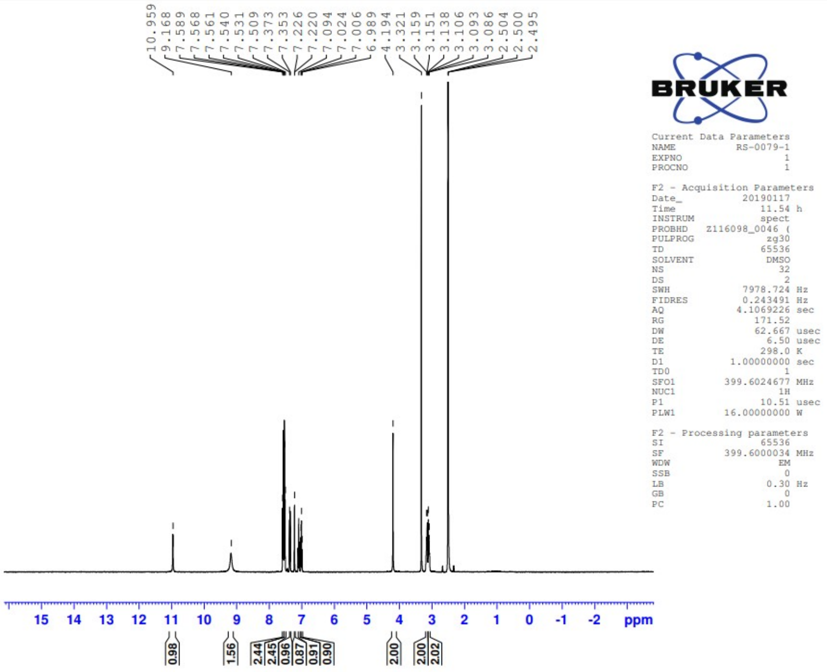

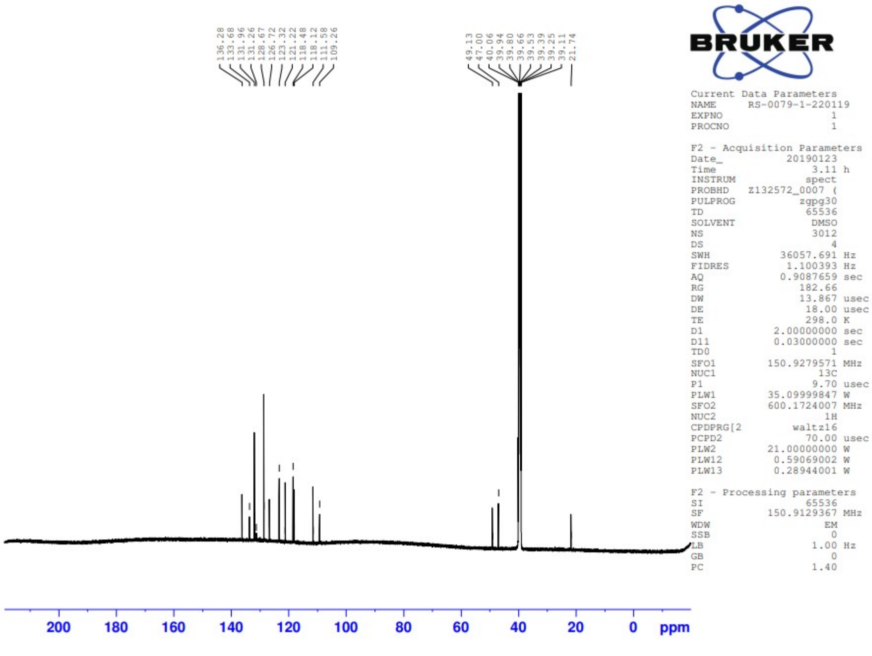

N-(4-chlorobenzyl)-2-(1-methyl-1H-indol-3-yl)ethan-1-amine HCl (13).

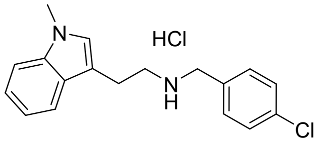

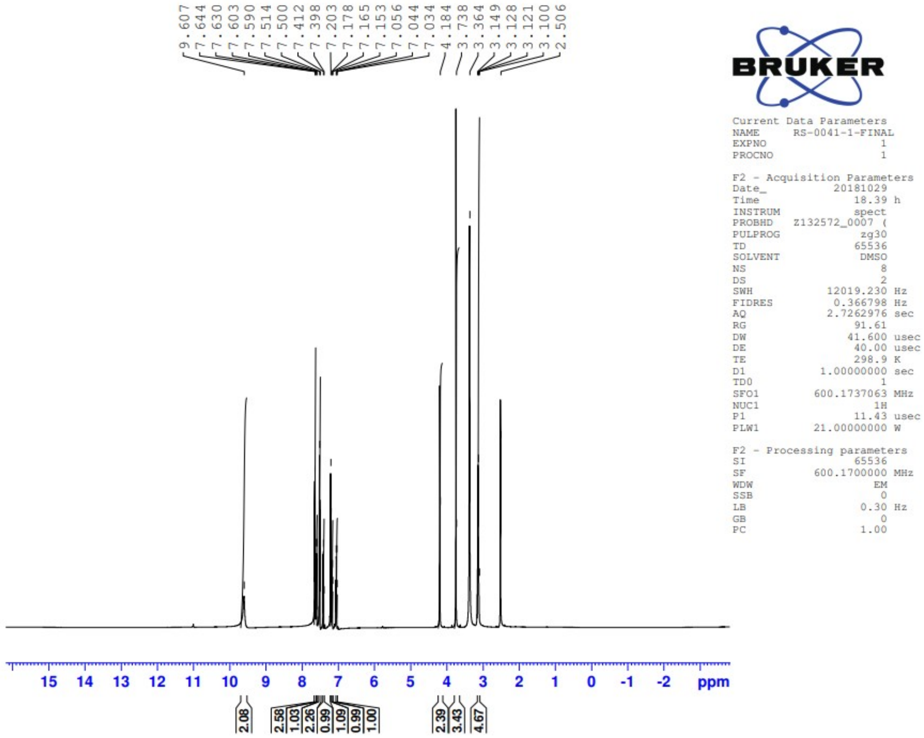

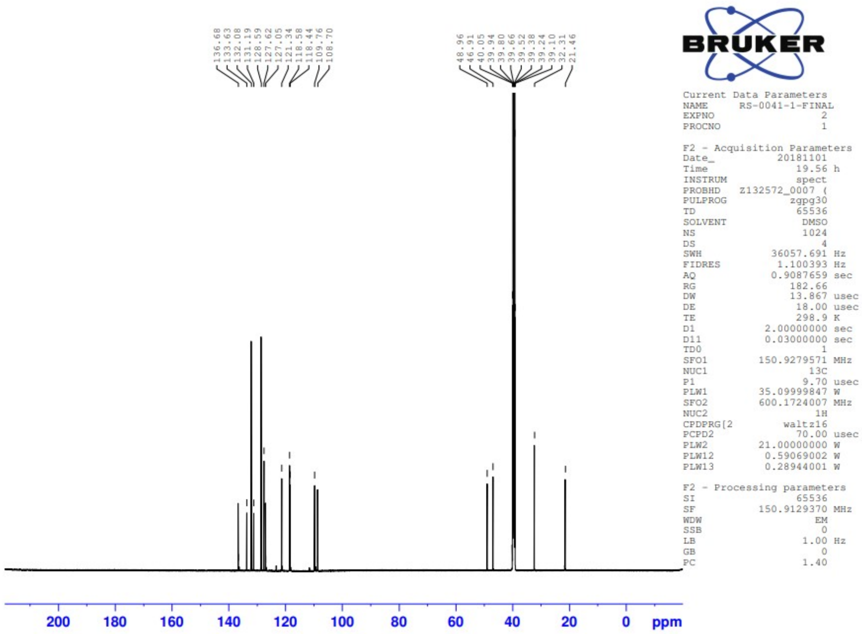

2-(1-methyl-1H-indol-3-yl)-N-(4-methylbenzyl)ethan-1-amine HCl (14).

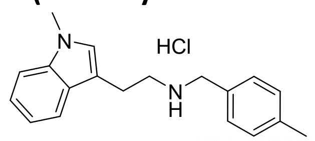

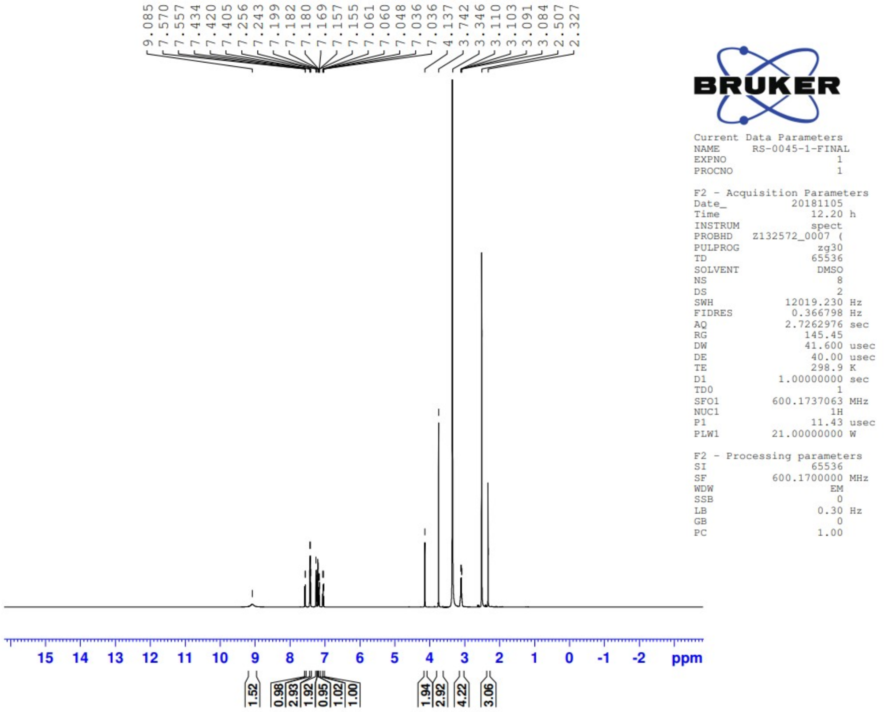

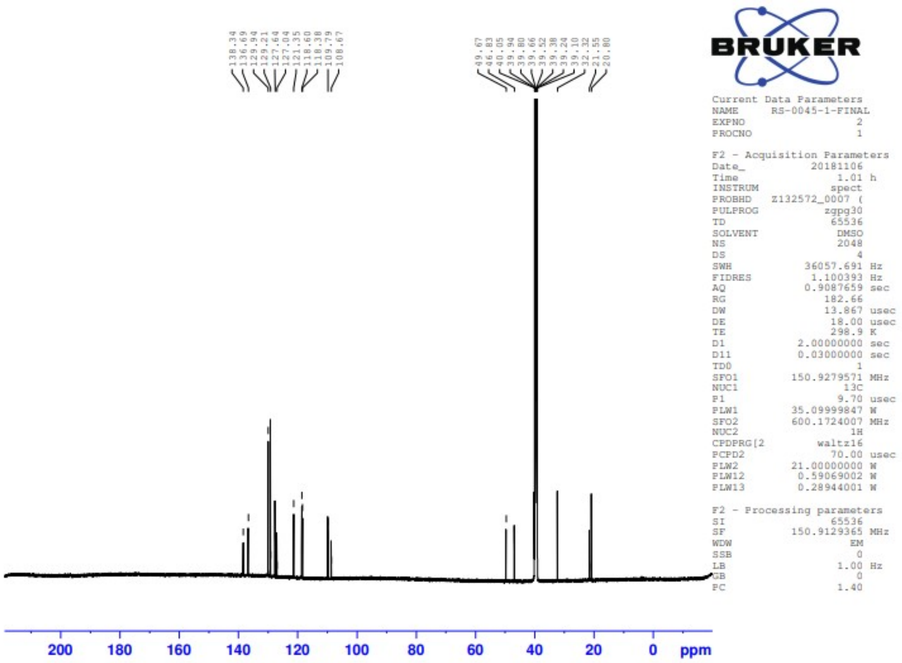

N-(3-chloro-4,5-dimethoxybenzyl)-2-(1H-indol-3-yl)ethan-1-amine (15).

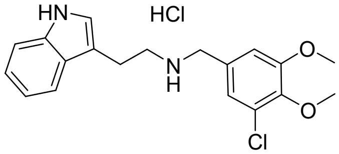

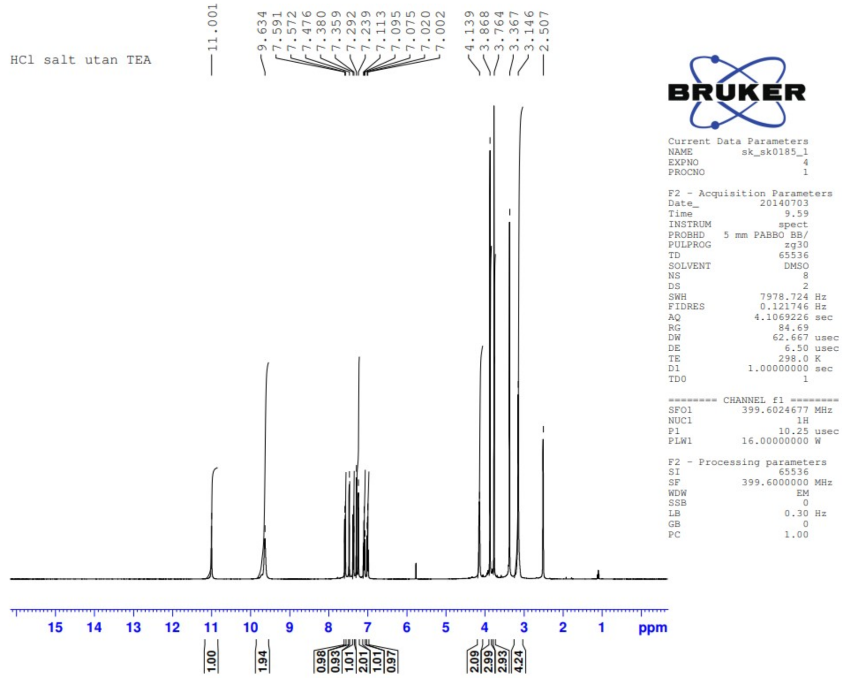

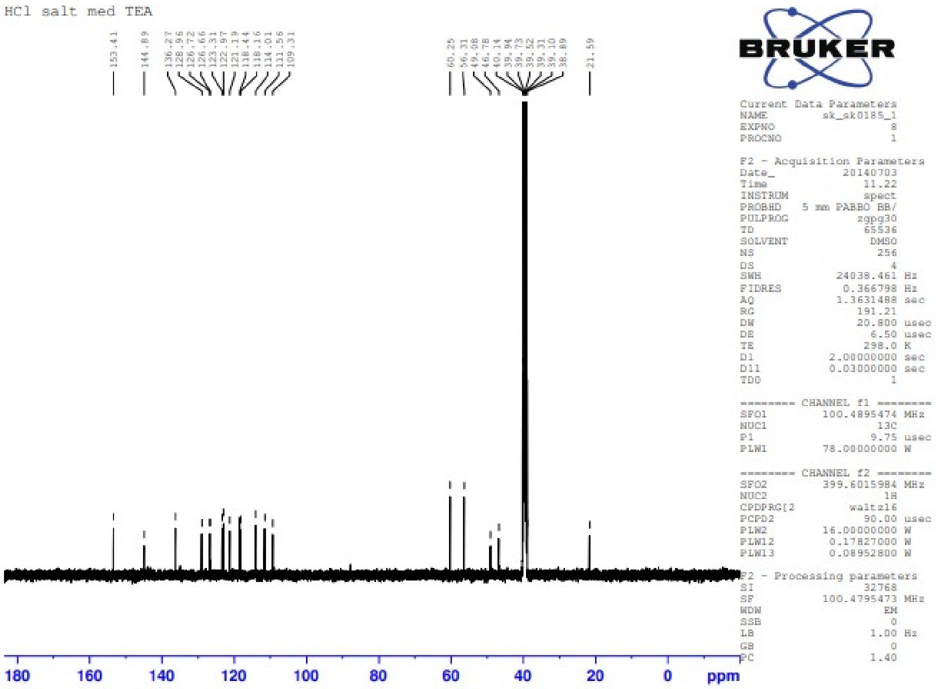

N-(3-chloro-4,5-dimethoxybenzyl)-2-(1-methyl-1H-indol-3-yl)ethan-1-amine HCl (16).

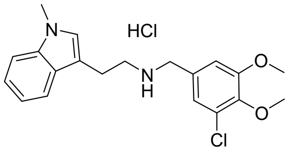

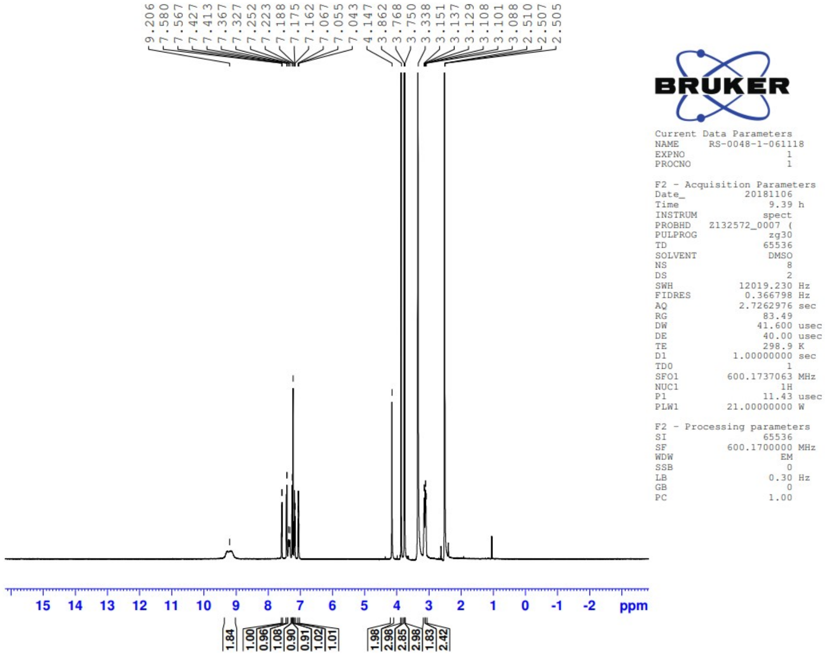

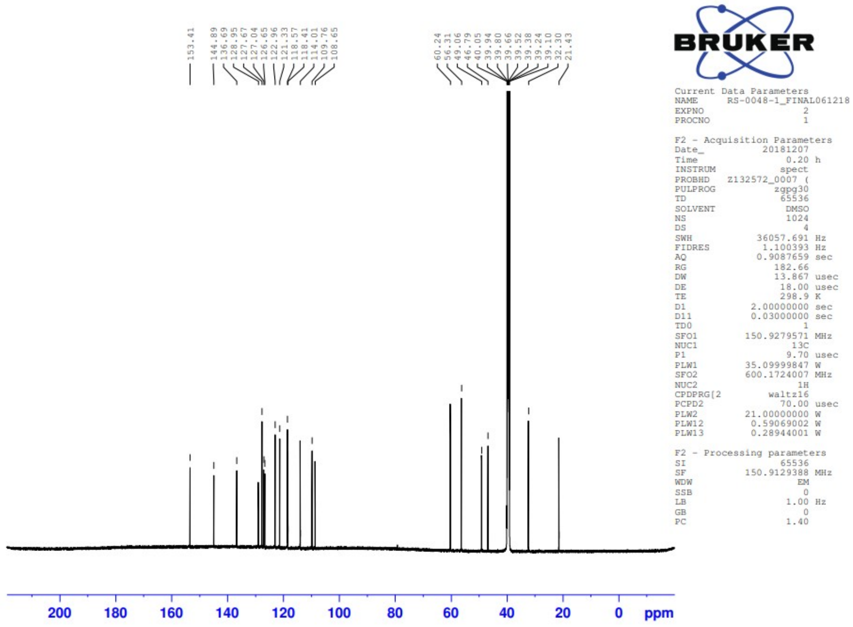

2-(1-methyl-1H-indol-3-yl)-N-(naphthalen-2-ylmethyl)ethan-1-amine HCl (17).

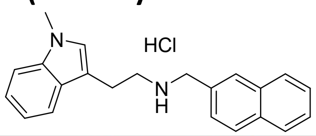

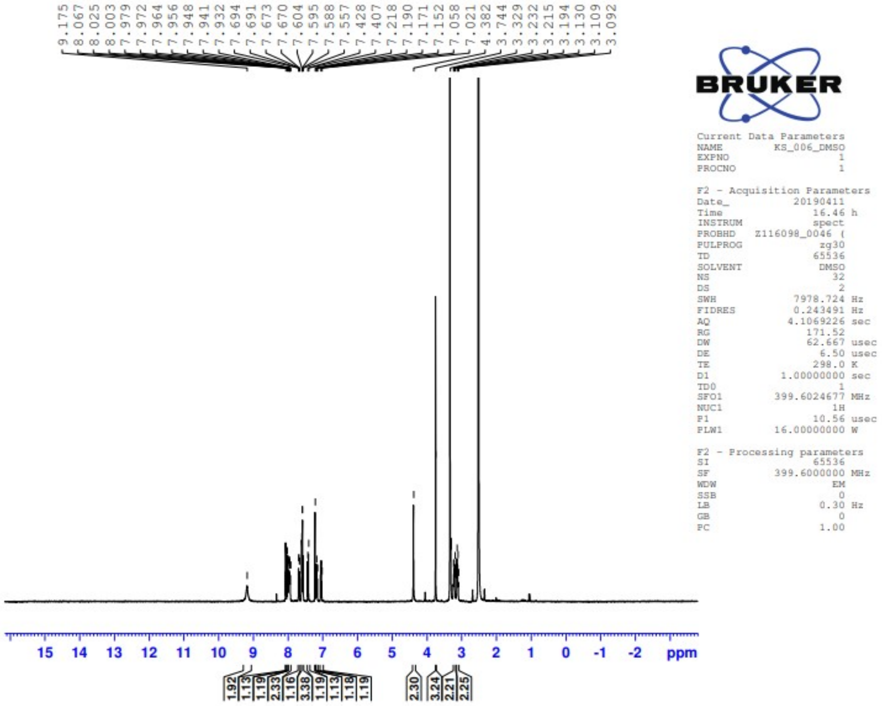

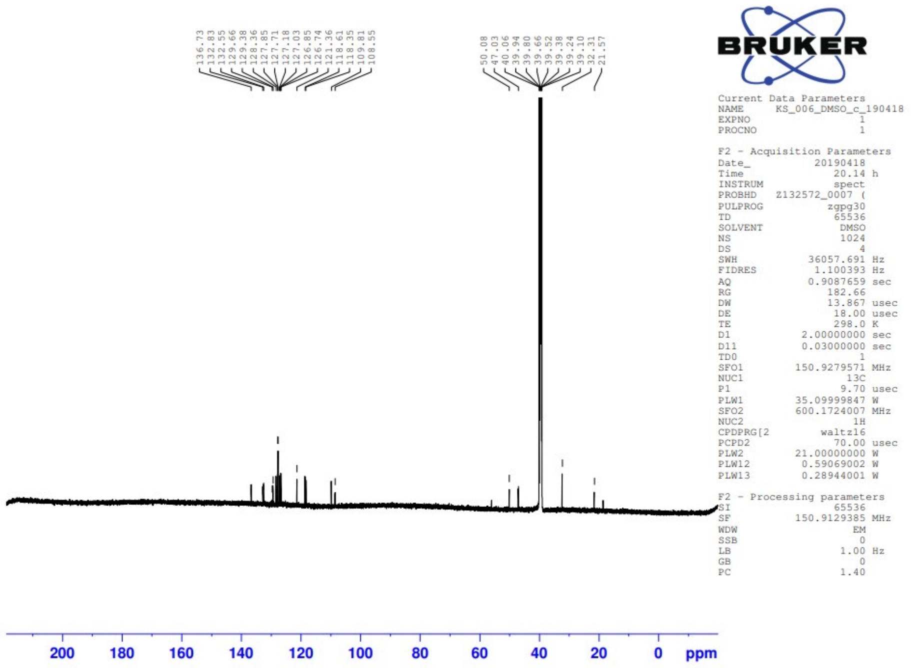

2-(5-chloro-1H-indol-3-yl)-N-(2-methoxybenzyl)ethan-1-amine HCl (18).

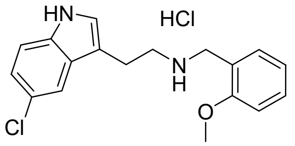

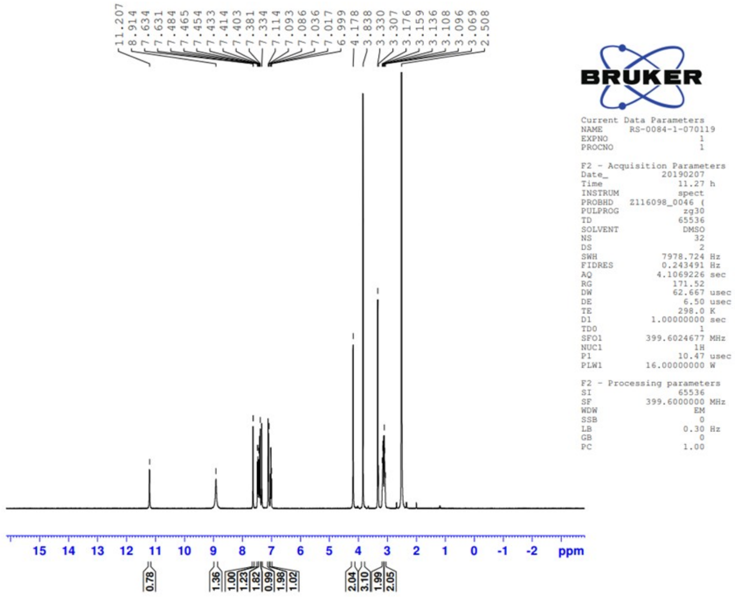

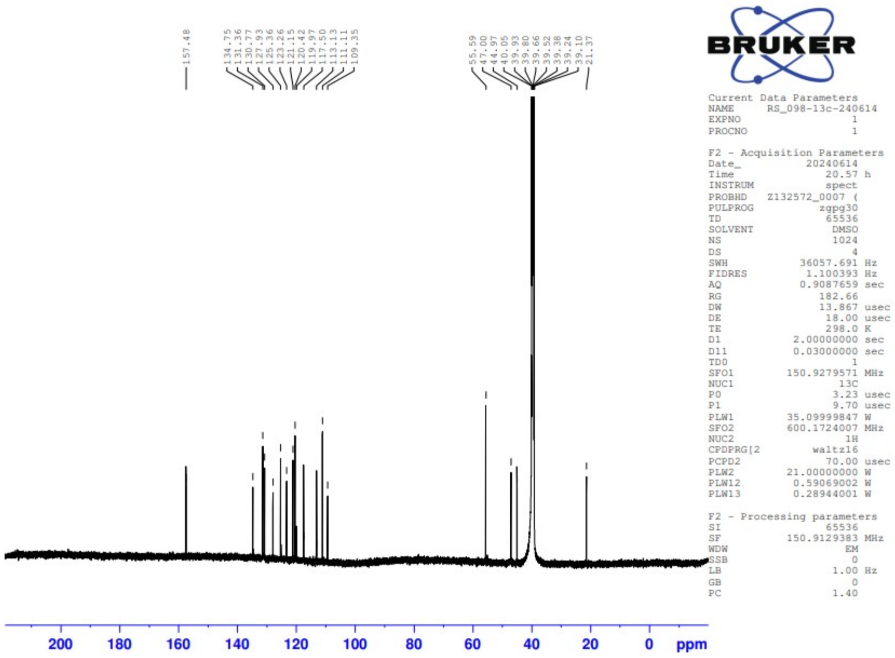

2-(5-chloro-1-methyl-1H-indol-3-yl)-N-(2-methoxybenzyl) ethan-1-amine HCl (19).

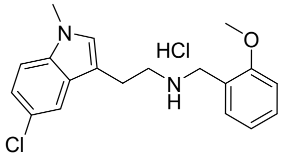

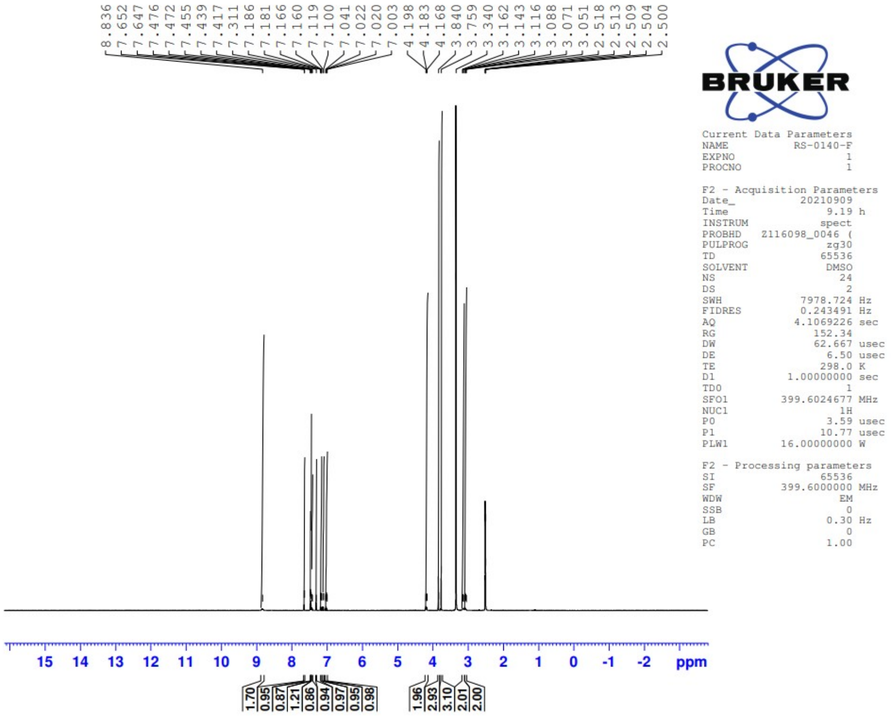

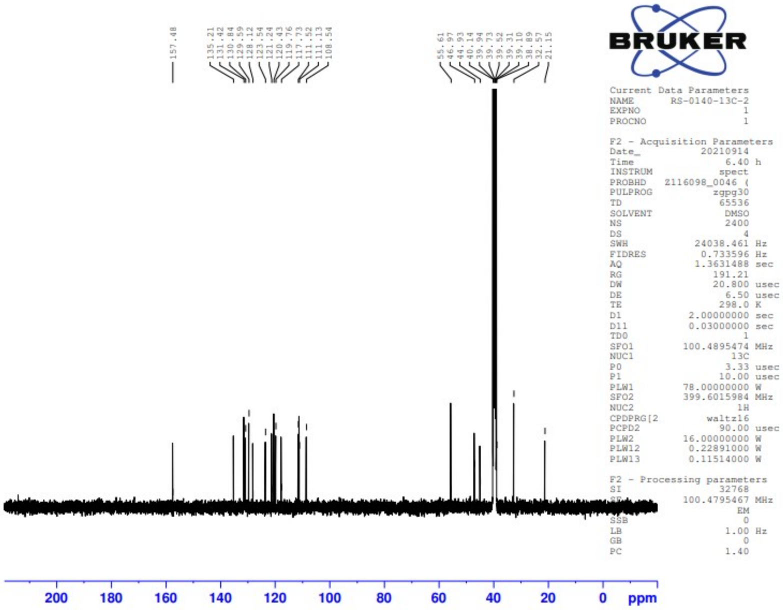

N-(2-methoxybenzyl)-2-(5-methyl-1H-indol-3-yl)ethan-1-amine HCl (20).

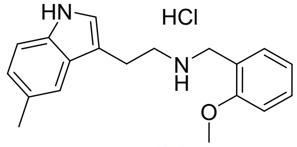

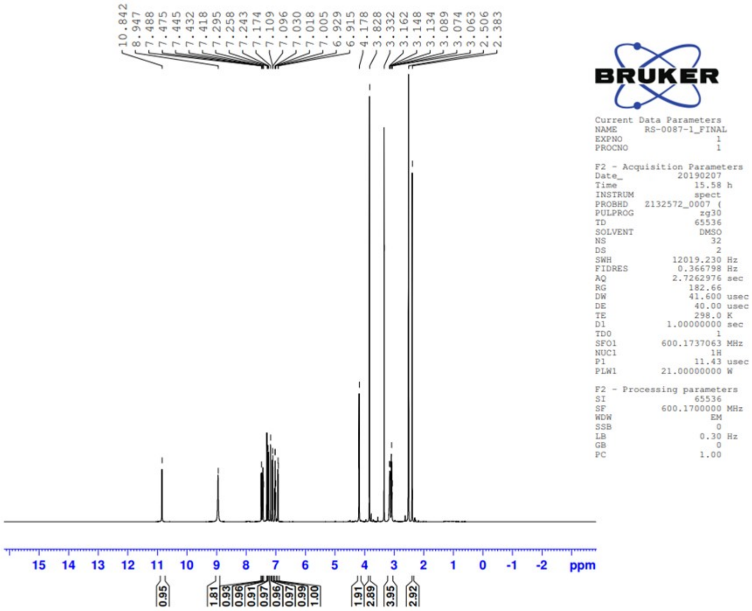

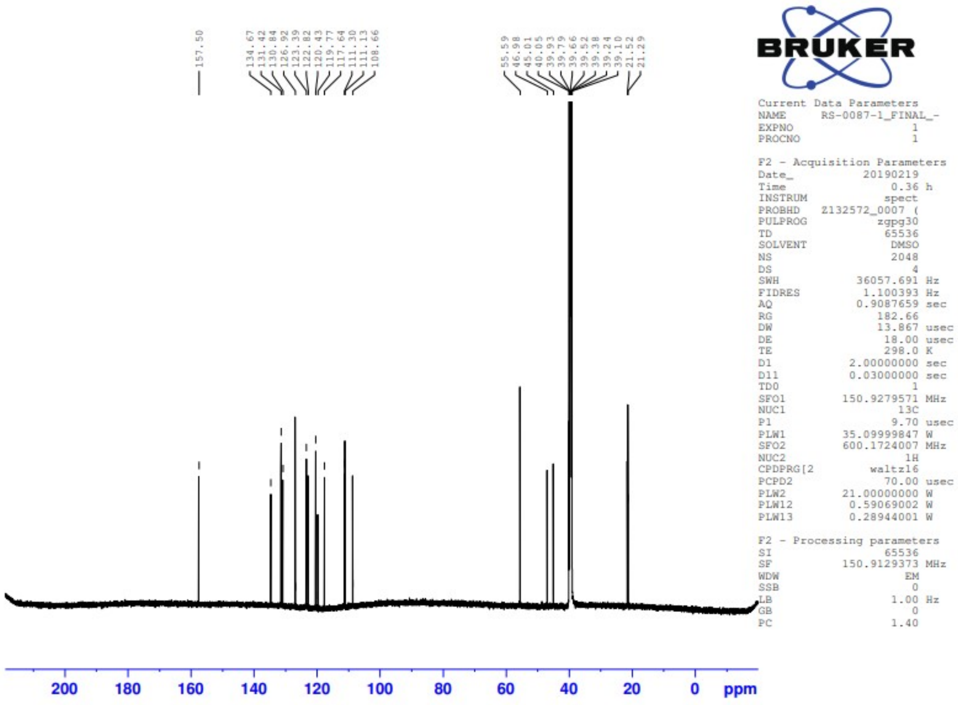

2-(6-fluoro-1H-indol-3-yl)-N-(2-methoxybenzyl)ethan-1-amine HCl (21).

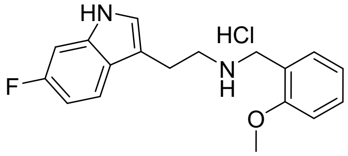

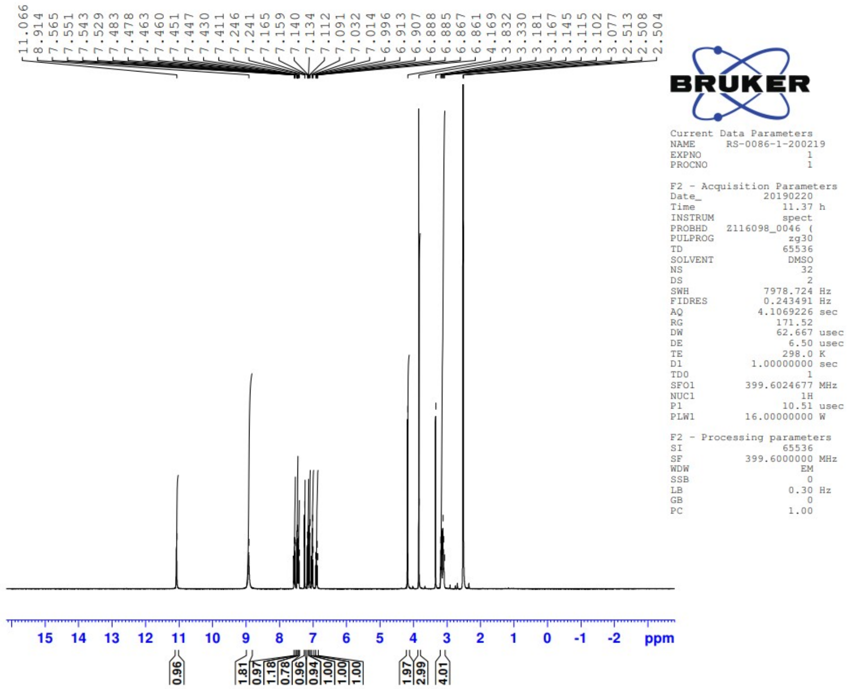

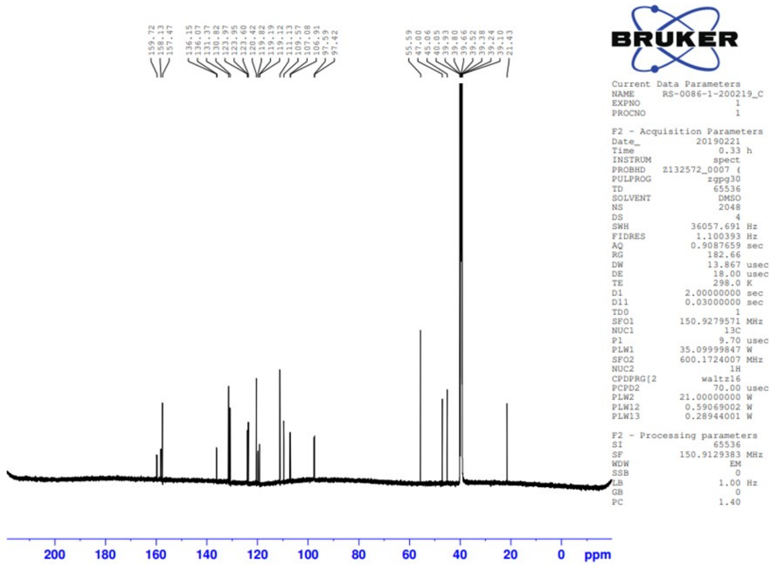

2-(6-fluoro-1-methyl-1H-indol-3-yl)-N-(2-methoxybenzyl) ethan-1-amine HCl (22).

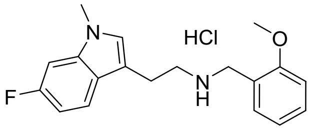

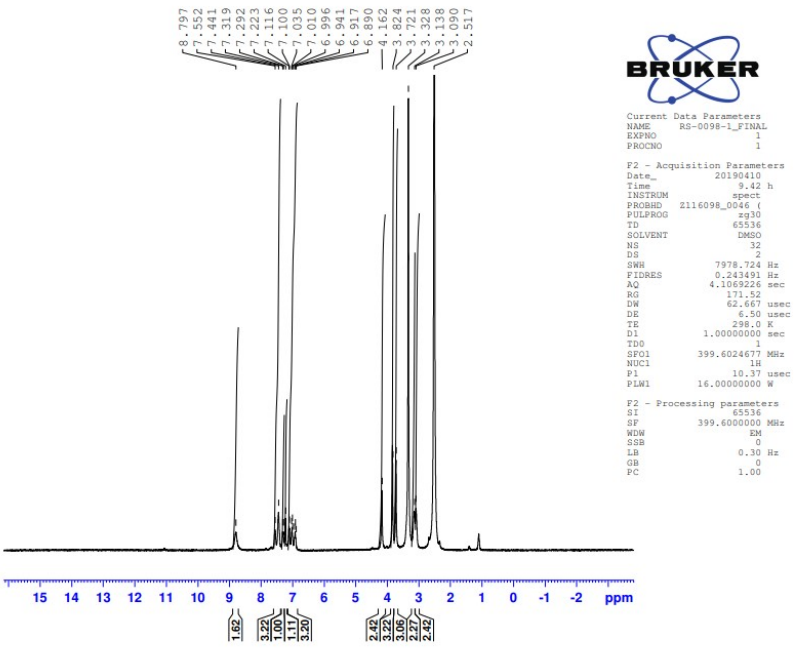

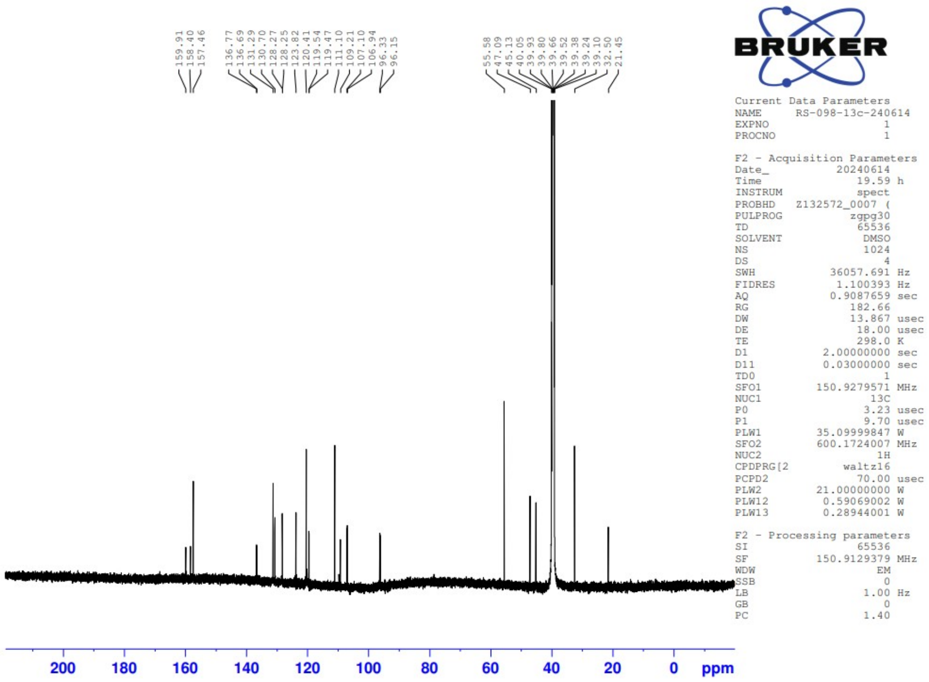

2-(5-methoxy-1H-indol-3-yl)-N-(2-methoxybenzyl)ethan-1-amine HCl (23).

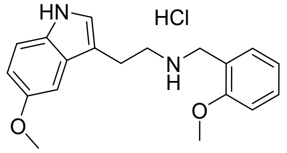

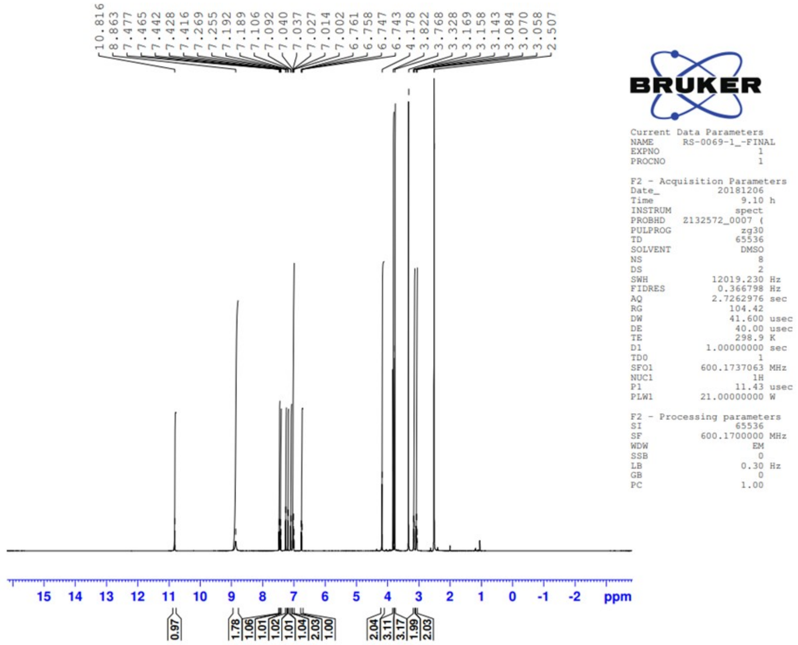

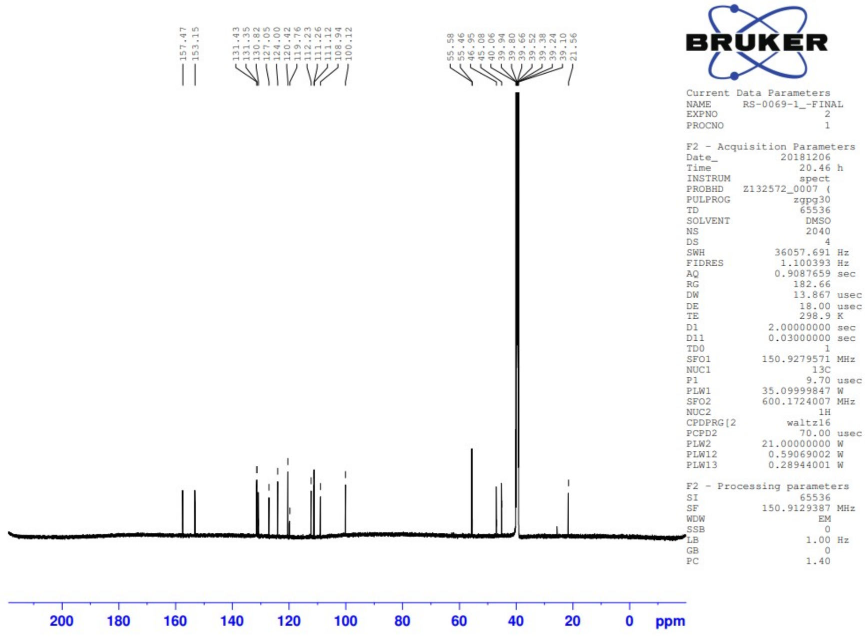

2-(5-methoxy-1-methyl-1H-indol-3-yl)-N-(2-methoxybenzyl) ethan-1-amine HCl (24).

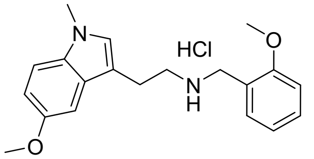

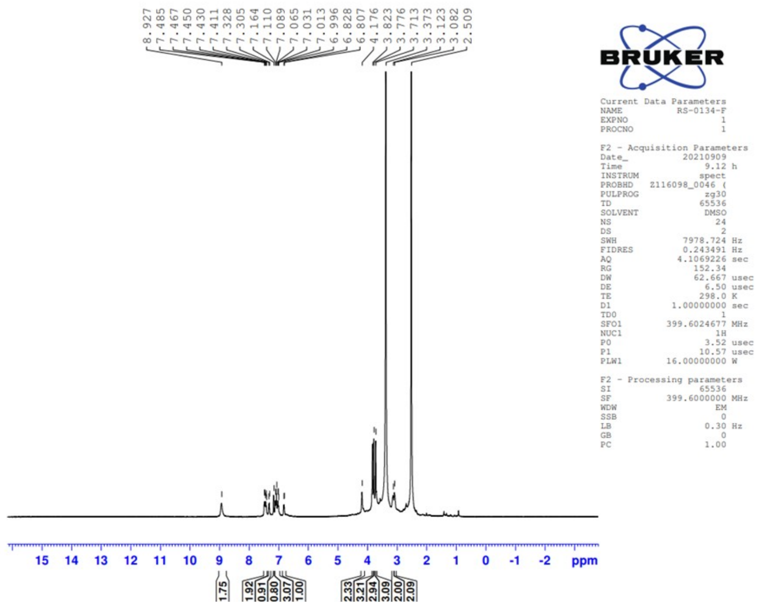

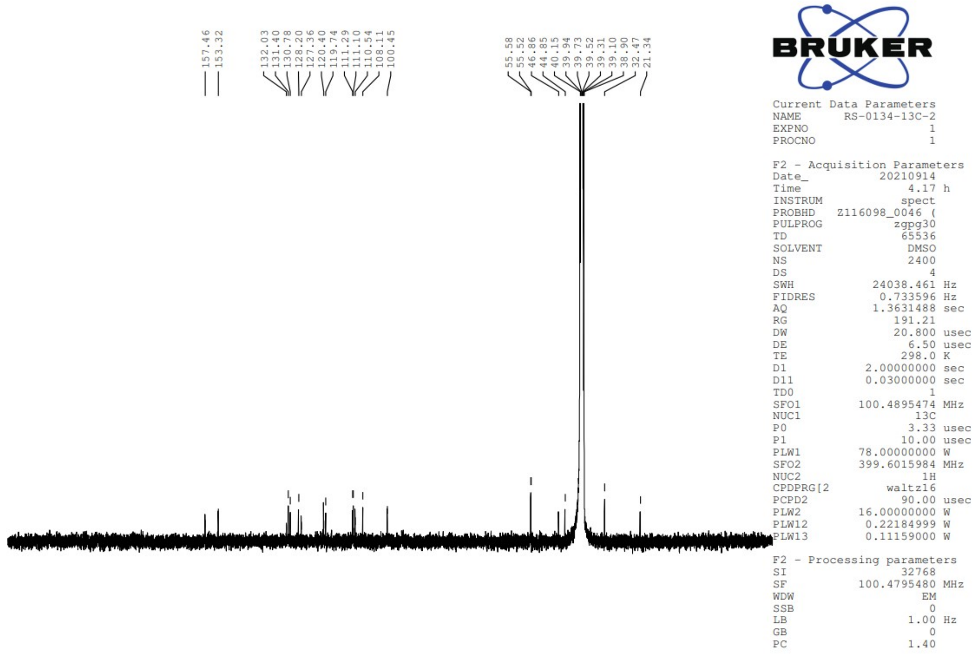

3-(2-((2-methoxybenzyl)amino)ethyl)-1H-indol-5-ol HCl (25).

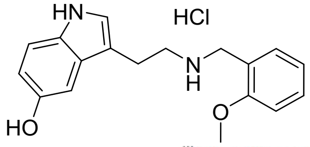

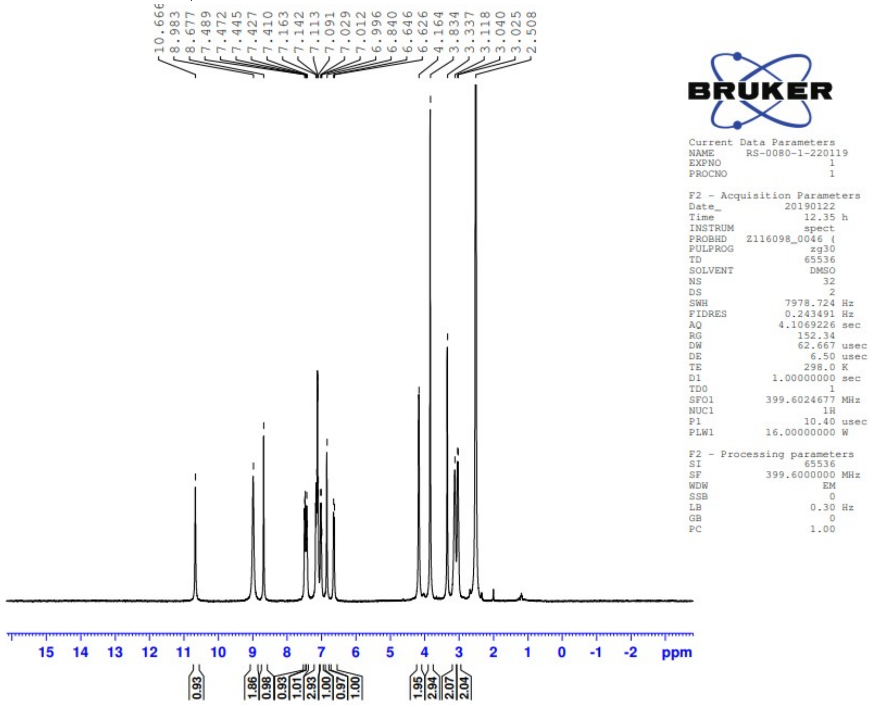

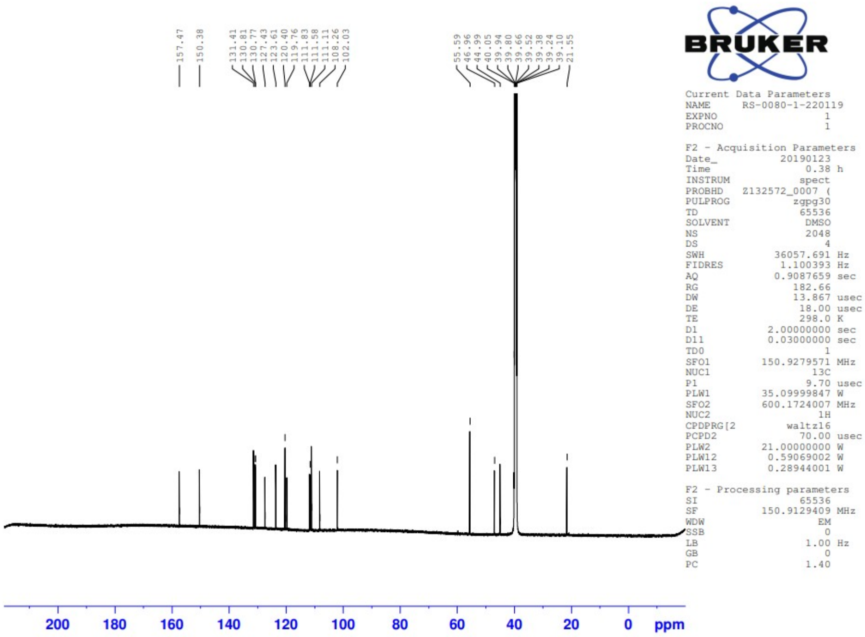

3-(2-((2-methoxybenzyl)amino)ethyl)-1-methyl-1H-indol-5-ol HCl (26).

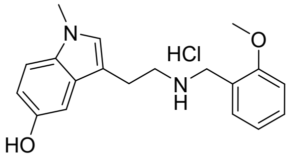

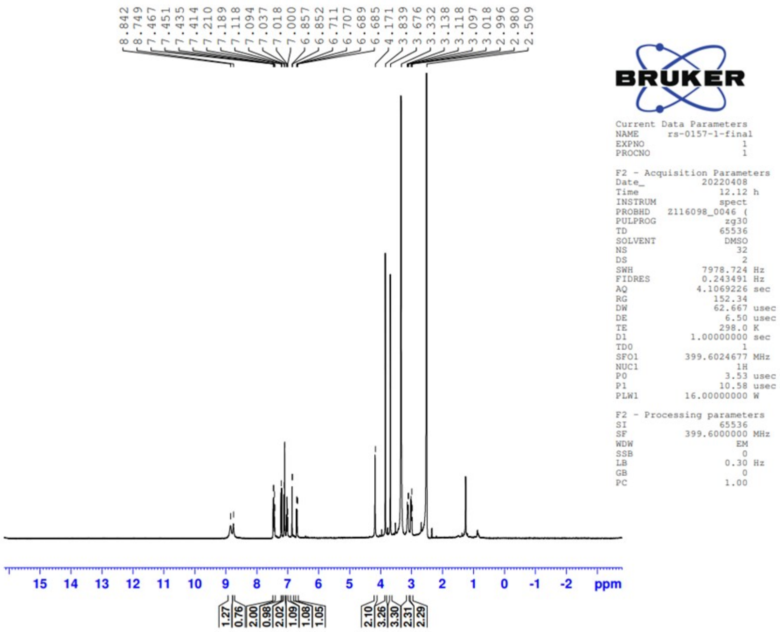

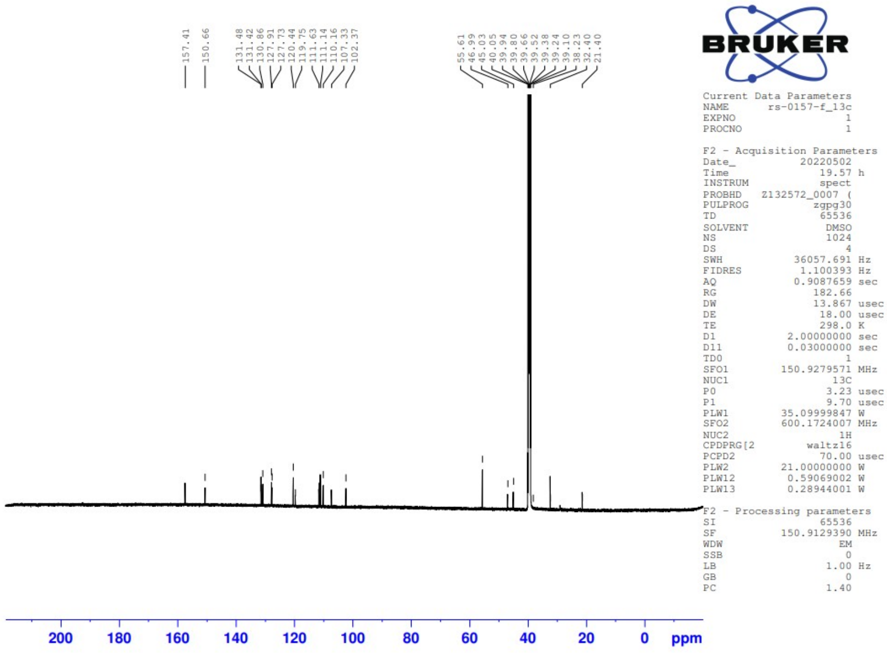

N-benzyl-N-methyl-2-(1-methyl-1H-indol-3-yl)ethan-1-amine HCl (29).

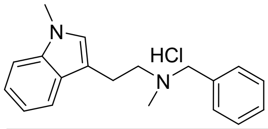

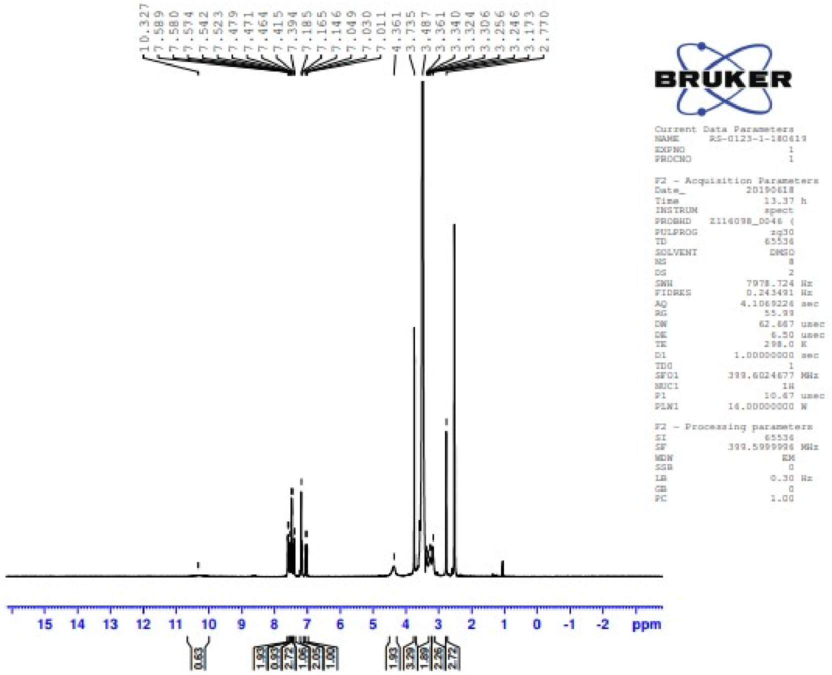

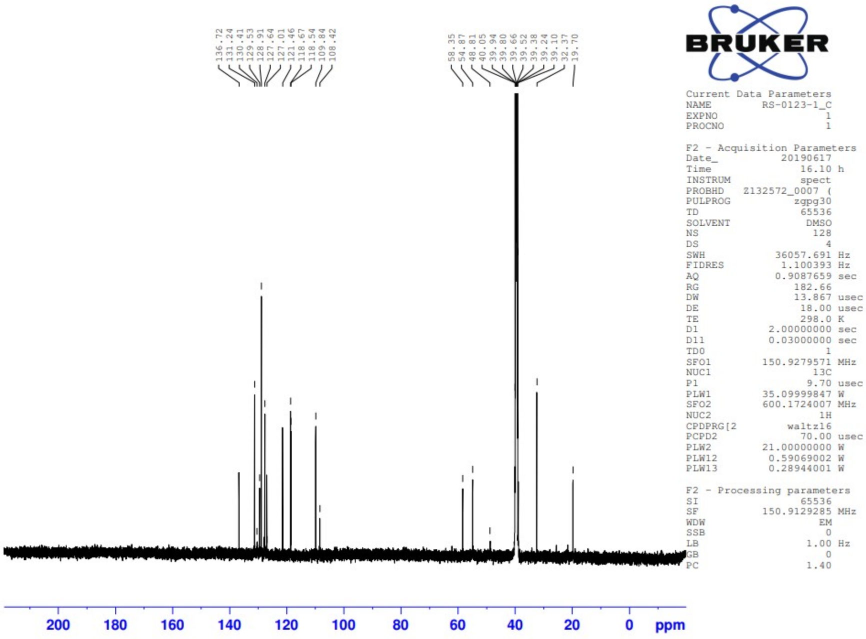

N-benzyl-N,N-dimethyl-2-(1-methyl-1H-indol-3-yl)ethan-1-aminium (30).

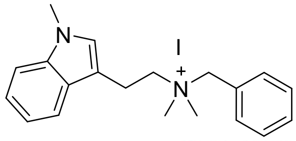

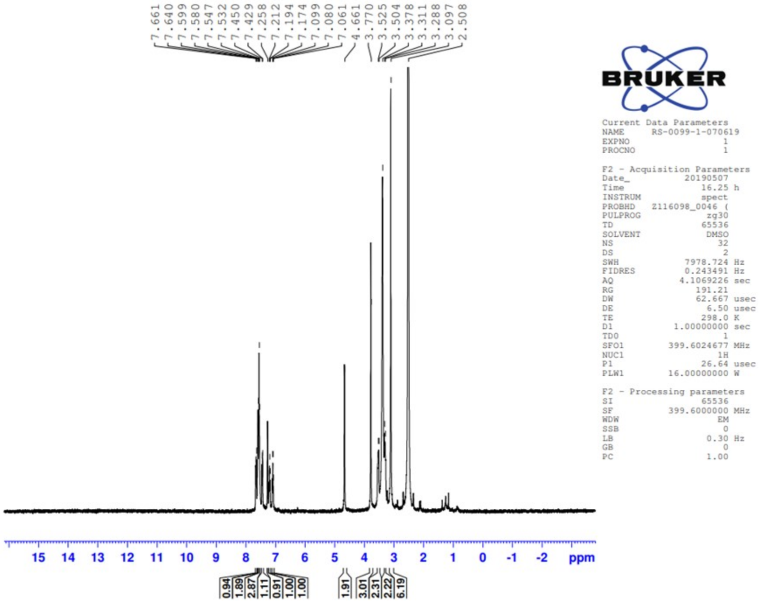

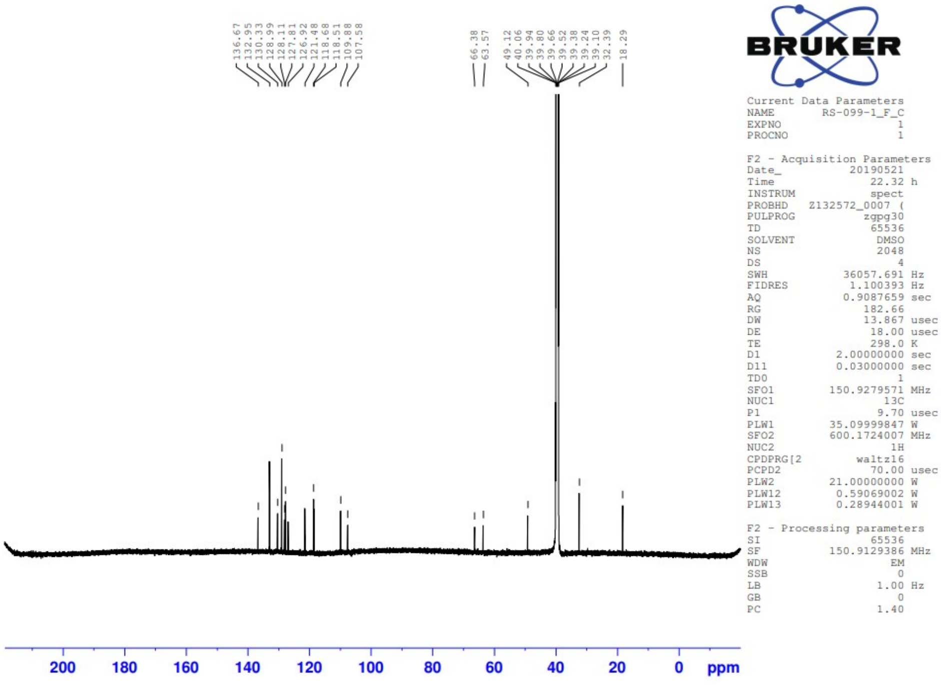

1-(2-methoxyphenyl)-6-methyl-2,3,4,9-tetrahydro-1H-pyrido[3,4-b]indole (31).

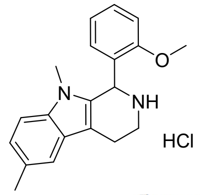

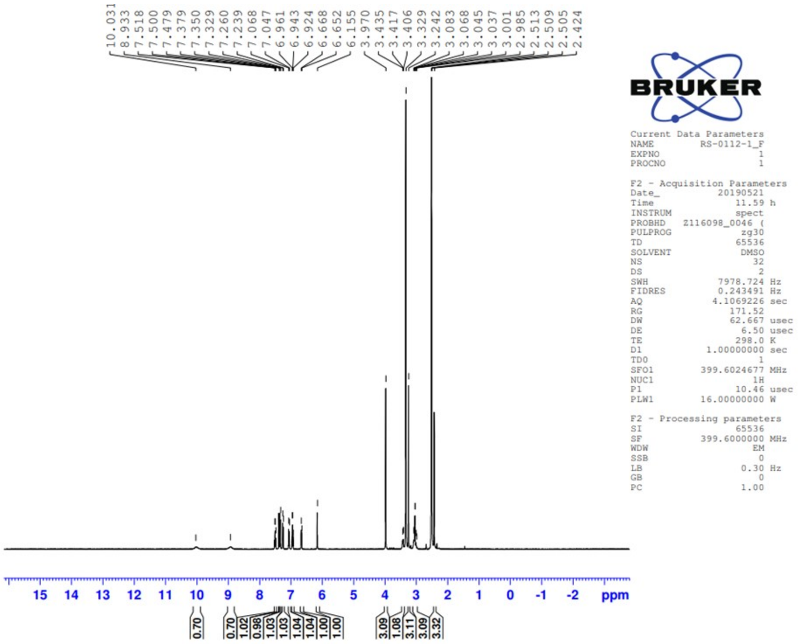

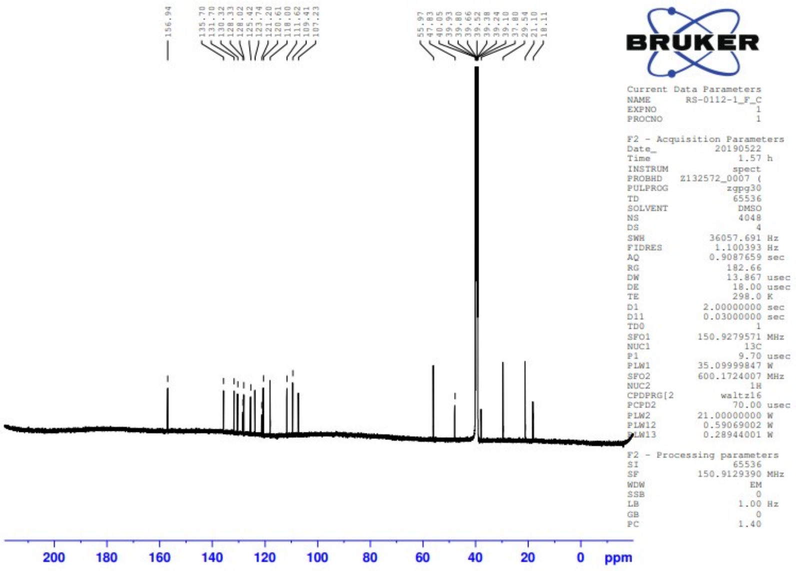

7-fluoro-1-phenyl-2,3,4,9-tetrahydro-1H-pyrido[3,4-b]indole HCl (32).

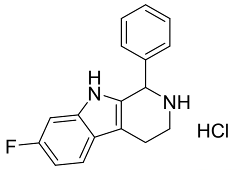

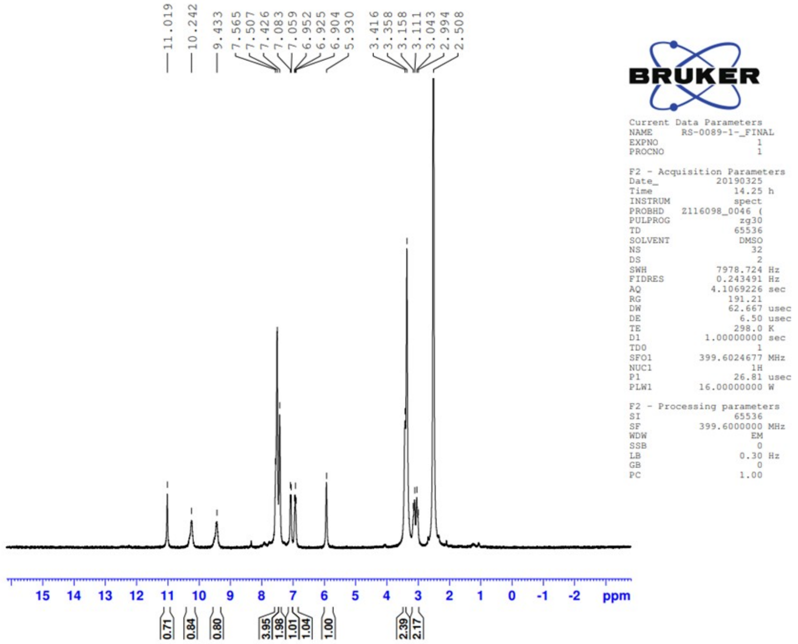

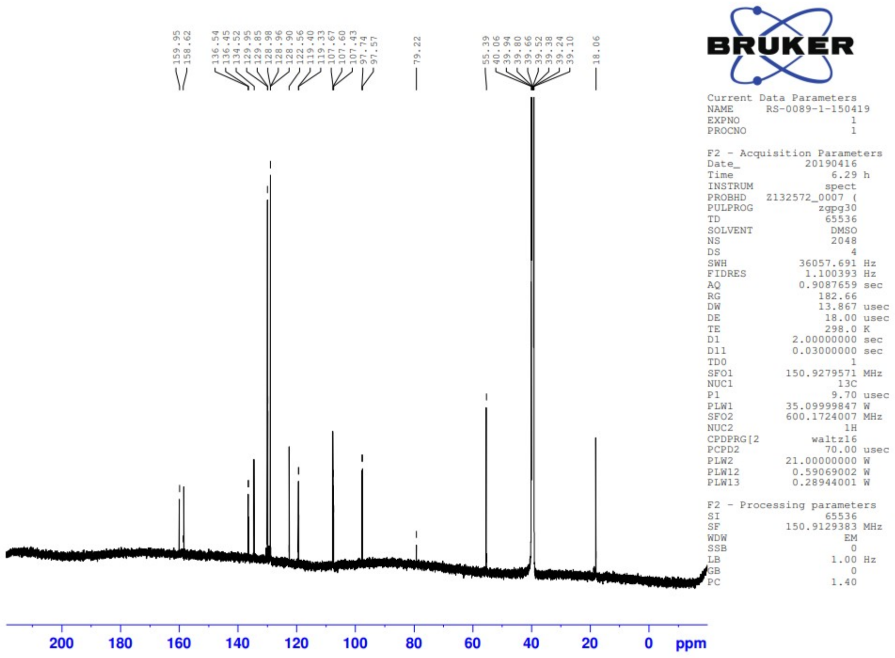

7-fluoro-1-(2-methoxyphenyl)-2,3,4,9-tetrahydro-1H-pyrido[3,4-b]indole (33).

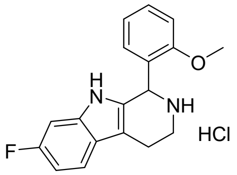

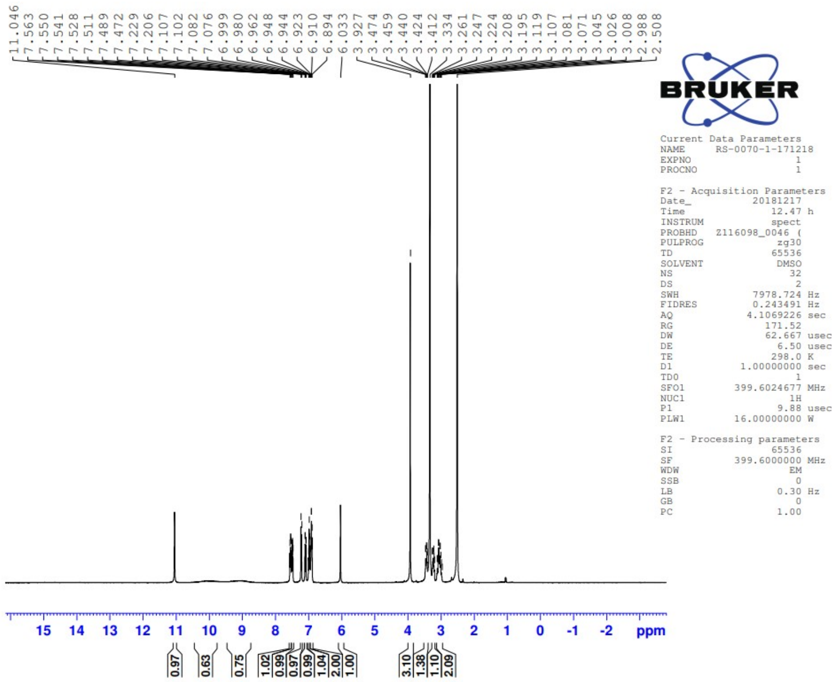

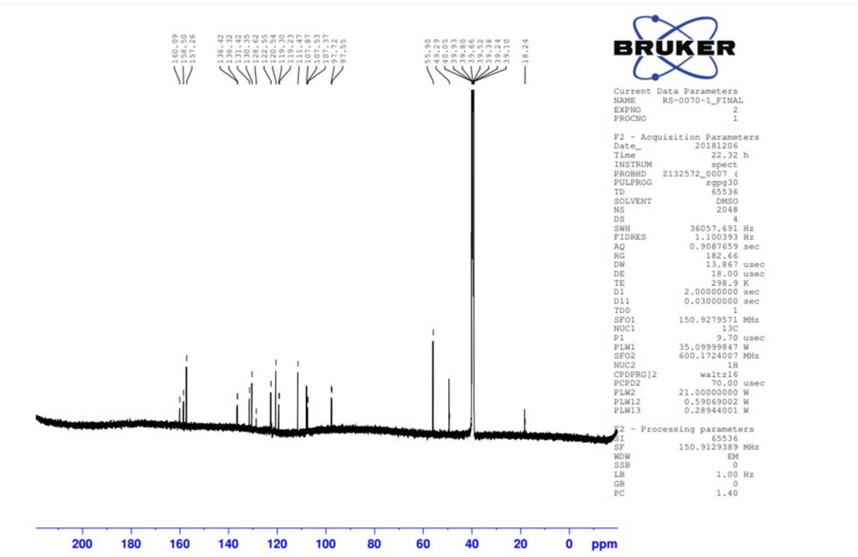

Supplement: MD-017-D5MD00797F-s001 [file MD-017-D5MD00797F-s001.pdf]
